# Revealing the stygobiotic and crenobiotic molluscan biodiversity hotspot in Caucasus: Part I. The phylogeny of stygobiotic Sadlerianinae Szarowska, 2006 (Mollusca, Gastropoda, Hydrobiidae) from Georgia with descriptions of five new genera and twenty-one new species

**DOI:** 10.3897/zookeys.955.51983

**Published:** 2020-08-05

**Authors:** Jozef Grego, Levan Mumladze, Andrzej Falniowski, Artur Osikowski, Aleksandra Rysiewska, Dimitry M. Palatov, Sebastian Hofman

**Affiliations:** 1 Horná Mičiná 219, 97401, Banská Bystrica, Slovakia; 2 Institute of Zoology, Ilia State University, Kakutsa Cholokashvili Ave 3/5, Tbilisi 0162, Georgia; 3 Department of Malacology, Institute of Zoology and Biomedical Research, Jagiellonian University, ul. Gronostajowa 9, 30-387, Kraków, Poland; 4 Department of Animal Reproduction, Anatomy and Genomics, University of Agriculture in Krakow, al. Mickiewicza 24/28, 30-059, Kraków, Poland; 5 Department of Hydrobiology, Biological Faculty, Moscow State University, 1-12 Leninskie Gory, 119991, Moscow, Russian Federation; 6 Department of Comparative Anatomy, Institute of Zoology and Biomedical Research, Jagiellonian University, ul. Gronostajowa 9, 30-387, Kraków, Poland

**Keywords:** cave, biodiversity, freshwater, interstitial, mtDNA, molecular taxonomy, spring, subterranean

## Abstract

The position of the southwestern Caucasus as a stygobiotic Mollusca hotspot is confirmed. Molecular data of stygobiotic gastropods revealed the diversity of subfamily Sadlerianinae Szarowska, 2006, inhabiting the subterranean environment of Georgia. In addition to the well-known endemic genera *Pontohoratia* Vinarski, Palatov & Glöer, 2014 and *Motsametia* Vinarski, Palatov & Glöer, 2014, five more genera were identified in northwestern Georgia as new to the science: *Kartvelobia***gen. nov.**, *Imeretiopsis***gen. nov.**, *Caucasopsis***gen. nov.**, *Caucasogeyeria***gen. nov.**, and *Hausdorfenia***gen. nov.** Additionally, 21 new species were found to inhabit the studied area (Samegrelo, Imereti, Racha regions in Georgia).

## Introduction

The southwestern Great Caucasus hosts a remarkable biodiversity of stygobiont and crenobiont molluscan species. The stygobiont fauna of the region was brought to the attention of the scientific community by Shadin (1932) from Rion Cave (= Iazoni = Tskal-Tsitela Cave) near Kutaisi with the description of *Horatia borutzkii* (= *Motsametia borutzkii* (Shadin, 1932)) and *Pisidium subterraneum* (= *Euglesa subterranea* (Shadin, 1932)). Later Shadin (1952) described the *Pisidum cavaticum* (= *Euglesa cavatica* (Shadin, 1952)) from Uschelna Cave at the upper Khosta River in Abkhazia. Tzvetkov (1940) had highlighted the presumably high stygobiont diversity of the region. Based on the field collection of Birstein (1959–1960) and Lyovuschkin (1961), 14 new stygobiont taxa were described by Starobogatov (1962) from western Great Caucasus: *Horatia birsteini* (= *Pontohoratia birsteini*); *Horatia sokolovi* (= *Pontohoratia birsteini*); *Horatia ljovuschkini* (= *Pontohoratia birsteini*); *Paladilhiopsis shadini*; *Paldilhiopsis subovata*; *Paladilhiopsis pulcherrima*; *Paladilhiopsis orientalis*; *Paladilhiopsis schakuranica*; *Paladilhopsis aculeus*; *Belgrandiella caucasica* (= *Tschernomorica caucasica*); *Belgrandiella abchasica* (= *Tschernomorica caucasica*); “*Geyeria*” (= *Plagigeyeria*) *valvataeformis*; “*Geyeria*” (= *Plagigeyeria*) *horatieformis*; *Pisidum ljovuschkini* (= *Euglesa ljovuschkini* (Starobogatov, 1962)). Type specimens of Yaroslav Igorevich Starobogatov with his species from the southwestern Caucasus deposited in ZIN St. Petersburg were figured by Sitnikova et al. in 2017. The figured “*Paladilhiopsis*” *subovata* and “*P.*” *orientalis* holotypes are represented only by fragments of broken shells, while the original descriptions depict drawings of complete shells. It is likely the fragile subfossil shells could have been damaged later, after the descriptions. For decades the stygobiont Mollusca of the Caucasus were not in the focus of malacologists. Only Schütt and Şeşen (1993) described a new species of *Belgrandiella nemethi* (= *Tschernomorica caucasica* (Starobogatov, 1962)), based on dry material collected by Lázló Németh near the village of Rostsvet in the Khosta valley. Vinarski et al. (2014) revised the species assigned to *Horatia*. Statistical conchological analysis revealed that three of the Starobogatov “*Horatia*” species were conspecific, and anatomical investigations supported the erection of the new genus *Pontohoratia* Vinarski, Palatov & Glöer, 2014. Shadin´s *Horatia borutzkii* species was transferred to the new genus *Motsametia* Vinarski, Palatov & Glöer, 2014, and a new species, *Pontohoratia smyri* Vinarski, Palatov & Glöer, 2014, was described from Novoafonskaya Cave. The Caucasian stygobiont species were included by Bole and Velkovrh (1986) in their “Mollusca from Continental Subterranean Aquatic Habitats”, listed by Kantor et al. (2010), by Barjadze et al. (2015), and by Vinarski and Kantor (2016). Chertoprud et al. (2016) summarised the species detected during their research in the southeast Caucasus. Nevertheless, it was clear from the above works and from recent studies of the Balkan fauna (Szarowska 2006; Falniowski at al. 2008; Beran et al. 2015, 2016; Rysiewska et al. 2017; Grego et al. 2018; Hofman et al. 2018; Osikowski et al. 2018; Grego et al. 2019), that the Caucasian species were assigned to respective Balkan genera (*Belgrandiella*, *Paladilhiopsis*, *Plagigeyeria*) based only on the few morphological features of the shell, and it is not likely that they are closely related to the geographically distant morphotypes. Molecular investigations of genus *Belgrandiella* from Georgia (Grego et al. 2017) revealed its close relationship to the genus *Agrafia* Szarowska & Falniowski, 2011 from Greece. Later Vinarski and Palatov (2019) transferred the Caucasus and Crimean members of *Belgrandiella* to a new genus, *Tschernomorica* Vinarski & Palatov, 2019, based on their anatomical differences from *Belgrandiella* Wagner, 1927 and *Agrafia*. Two new species of *Tschernomorica* from Caucasus were established: *T. inconspicua* (Vinarski & Palatov, 2019) and *T. lindholmi* Vinarski & Palatov, 2019.

In the present work we provide the results of our recent field work in the karst massifs of the southwestern Great Caucasus which revealed a remarkable diversity of the subterranean mollusc fauna. Based on morphological and genetic investigations, we here describe 21 new species in five new genera belonging to subfamily Sadlerianinae Szarowska, 2006 (Gastropoda, Hydrobiidae) and provide diagnostic features and distribution data.

## Materials and methods

**Figure 1.**
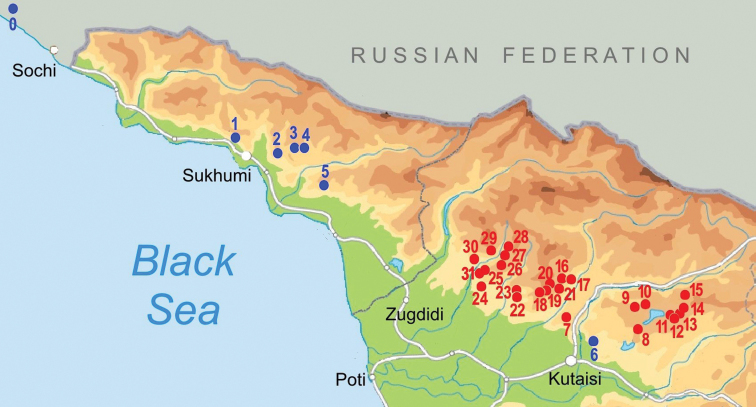
Map of stygobiotic Sadlerianinae distribution in the southwestern Caucasus: historical localities (blue dots) and new studied localities (red dots) **0** Krasnoalexandrovskaya Cave **1** New Athos Cave **2** Tsebedinska Cave **3** Srednaya Sakuranskaya Cave **4** Nizhnaya Sakuranskaya Cave **5** Abrskila Cave **6** Tskhal-Tsiteli Cave **7** Prometheus Cave **8** Nakerala Spring **9** small spring near Gorgoleti 3 **10** Tsivtskala 2 Spring near power station **11** Kidobana Cave **12** Cholaba Spring **13** Shakishore Cave **14** Dolabistavi Cave **15** Krikhula Spring **16** Kinchkha Cave and Spring and Kinchkha Waterfall **17** Kinchkhaperdi Spring **18** Upskhero Spring **19** Nakhriduri Spring 1 and 2 **20** Turchu Gamosadivari Cave **21** Turchusmtha Spring **22** Motena Cave **23** Pirveli Balda Spring **24** Nazodelavo Cave **25** Kachara Cave **26** Shisha Spring **27** Mapeli Cave **28** Shurubumu Springs **29** Kanti, Mapeli Spring **30** Letsurtsume Cave **31** Garakha, Savekuo Cavern.

**Figure 2.**
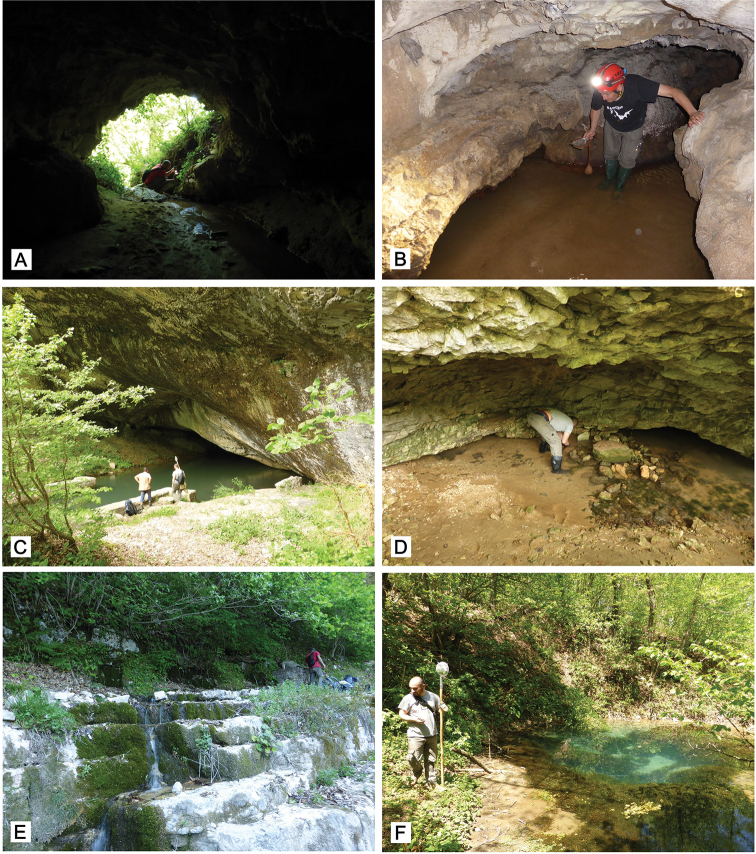
Photographs of studied localities in Georgia **A** Imereti, Kutaisi, Iazoni (Tskhal-Tsiteli) Cave, entrance **B** Tskalsithela Cave, cave stream **C** Racha, Nikorsminda, Shareula River Spring from Shareula Cave **D** Racha, Tsivtskala 2 Spring at left side of the Shareula River near power station **E** Imereti, Satsiskvilo, spring near Turchusmtha **F** Imereti, Zeda Gordi, Upskhero Spring at Turchu Gamosadivari Basin near Nakhriduri. Photograph M. Olšavský.

**Figure 3.**
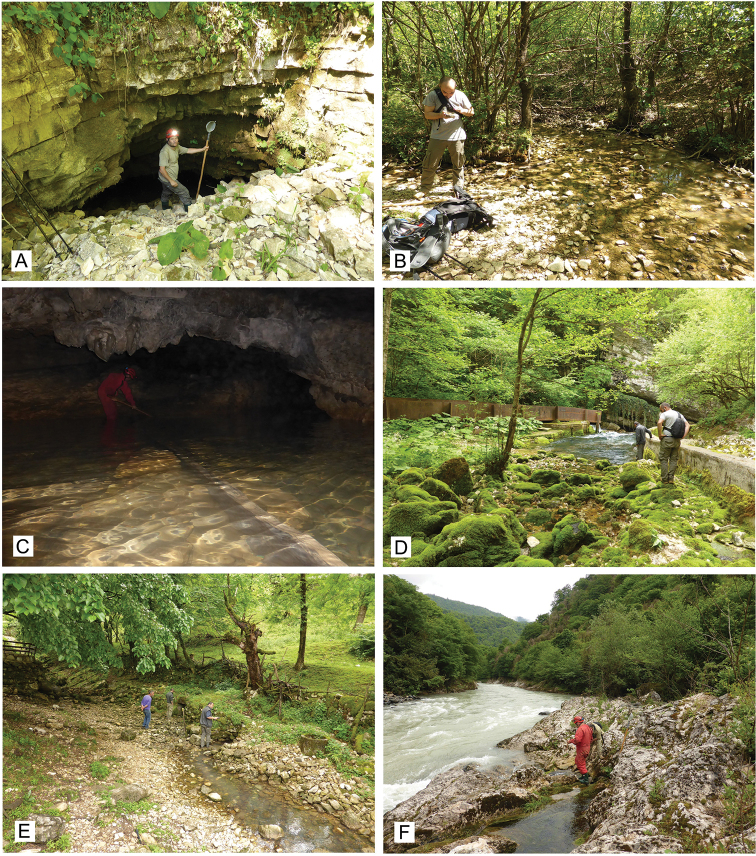
Photographs of studied localities in Georgia **A** Imereti, Zeda Gordi, Turchu Gamosadivari Cave spring **B** Imereti, Zeda Gordi, spring in Nakhriduri **C** Racha, Skhartali, Shakishore Cave **D** Racha, Shua Skhava, Krikhula Spring **E** Samegrelo, Pirveli Balda, spring in village **F** Samegrelo, Mukhuri, Shurubumu 1, spring at left bank of Khobistskali River. Photograph M. Olšavský.

**Figure 4.**
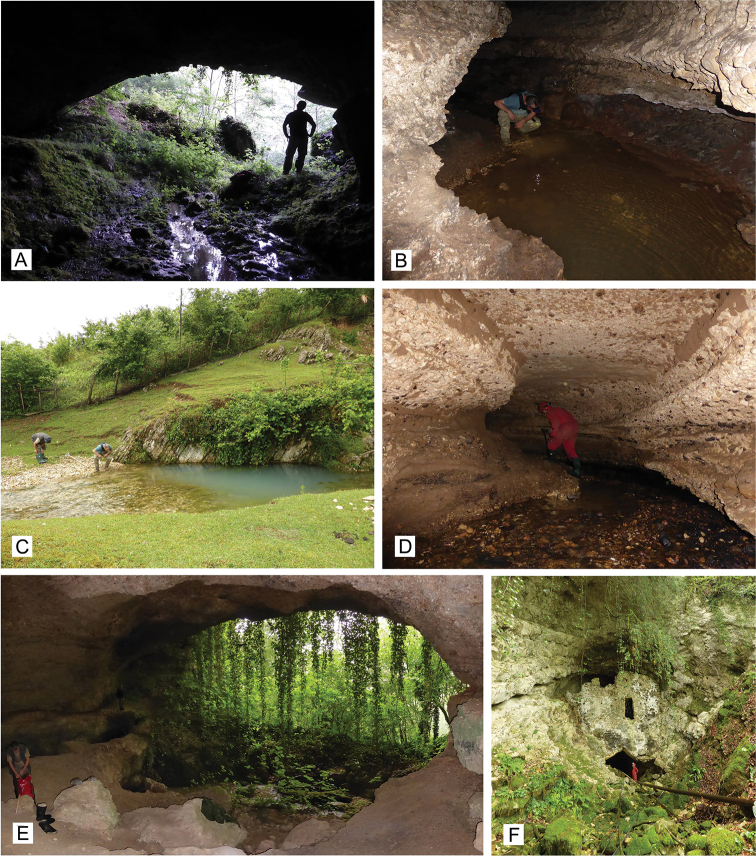
Photographs of studied localities in Georgia **A** Samegrelo, Chkhorotsku, Letsurtrume, Lesurtsume Cave entrance **B** Lesurtsume Cave stream **C** Samegrelo, Mukhuri, Shisha Spring **D** Samegrelo, Chkhorotsku, Kachara Cave **E** Samegrelo, Chkhorotsku, Nazodelavo Cave **F** Samegrelo, Pirveli Balda, Motena Cave. Photograph M. Olšavský.

The studied material was collected during field trips to the Samegrelo, Imereti, and Racha provinces of Georgia in 2018 and 2019 (Fig. 1). Different caves, spring outflows and karstic springs were sampled (Figs 1–4). Microhabitat preference and sampling methods were used as described by Grego et al. (2017). Samples of fine sand were freshly wet screened under a stereomicroscope to retrieve live animals. Then the samples were dried and screened again for shells that might have been overlooked during the wet screening. Frontal, ventral, and lateral view images of the shells were made by a Nikon SMZ25 microscope equipped with a Nikon D200 camera and an AF-S Micro NIKKOR 60 mm lens at the Vienna Natural History Museum (NHMW), Austria. ImageJ image analysis software was used to measure the specimens (Rueden et al. 2017). The shell morphology features were followed after Davis et al. (1992) and Hershler and Ponder (1998).

Snails for molecular analysis were fixed in 80% ethanol, changed twice, and later stored in 80% ethanol. DNA was extracted from whole specimens; tissues were hydrated in TE buffer (3 × 10 min); then total genomic DNA was extracted with the Sherlock extraction kit (A&A Biotechnology), and the final product was dissolved in 20 μl of tris-EDTA (TE) buffer. The extracted DNA was stored at −80 °C at the Department of Malacology, Institute of Zoology and Biomedical Research, Jagiellonian University in Kraków (Poland).

Mitochondrial cytochrome oxidase subunit I (COI) and nuclear histone 3 (H3) loci were sequenced. Details of PCR conditions, primers used, and sequencing were given in Szarowska et al. (2016). Sequences were initially aligned in the MUSCLE (Edgar 2004) Programme in MEGA 7 (Kumar et al. 2016) and then checked in Bioedit 7.1.3.0 (Hall 1999). Uncorrected p-distances were calculated in MEGA 7. The estimation of the proportion of invariant sites and the saturation test for entire data sets (Xia 2000; Xia et al. 2003) were performed using DAMBE (Xia 2013). In the phylogenetic analysis, additional sequences from GenBank were used as reference (Table 1). The data were analysed using approaches based on Bayesian Inference (BI) and Maximum Likelihood (ML). In the BI analysis, the GTR + I + Γ model of nucleotide substitution was applied. Model was selected using MrModelTest 2.3 (Nylander 2004). The analyses were run using MrBayes v. 3.2.3 (Ronquist et al. 2012) with default of most priors. Two simultaneous analyses were performed, each with 10,000,000 generations, with one cold chain and three heated chains, starting from random trees and sampling the trees every 1,000 generations. The first 25% of the trees were discarded as burn-in. The analyses were summarised as a 50% majority-rule tree. Convergence was checked in Tracer v. 1.5 (Rambaut and Drummond 2009). The Maximum Likelihood analysis was conducted in RAxML v. 8.2.12 (Stamatakis 2014) using the ‘RAxML-HPC v.8 on XSEDE (8.2.12) tool via the CIPRES Science Gateway (Miller et al. 2010). We applied the GTR model which is the only nucleotide substitution model implemented in RaxML, whose parameters were estimated by RaxML (Stamatakis 2014). Two species delimitation methods were performed: Poisson Tree Processes (PTP) (Zhang et al. 2013) and Automatic Barcode Gap Discovery (ABGD). The PTP approach was run using the web server https://species.h-its.org/ptp/, with 100,000 MCMC generations, 100 thinning and 0.1 burn-in. We used RAxML output phylogenetic tree. The ABGD approach using the web server (https://bioinfo.mnhn.fr/abi/public/abgd/abgdweb.html) and the default parameters.

The conservation status evaluation of each newly described species is based on the categories and criteria of IUCN and recommendations provided by Cardoso et al. (2011).

**Table 1.** Taxa used for phylogenetic analyses with their GenBank accession numbers and references.

Species	COI/H3 GB numbers	References
*Agrafia wiktori* Szarowska & Falniowski, 2011	JF906762/MG543158	Szarowska and Glöer 2011/Grego et al. 2017
*Belgrandiella kusceri* (Wagner, 1914)	KT218511/MG551366	Falniowski and Beran 2015/Osikowski et al. 2018
*Bithynia tentaculata* (Linnaeus, 1758)	AF367643/–	Wilke et al. 2001
*Bythinella austriaca* (von Frauenfeld, 1857)	JQ639858/–	Falniowski et al. 2012b
*Bythiospeum acicula* (Hartmann, 1821)	KU341350/ MK609536	Richling et al. 2016/ Falniowski et al. 2019
*Ecrobia maritima* (Milaschewitsch, 1916)	KX355830/MG551322	Osikowski et al. 2016/Grego et al. 2017
*Emmericia expansilabris* Bourguignat, 1880	KC810060/–	Szarowska and Falniowski 2013
*Hauffenia michleri* Kuščer, 1932	KY087865/KY087878	Rysiewska et al. 2017
*Heleobia maltzani* (Westerlund, 1886)	KM213723/ MK609534	Szarowska et al. 2014/ Falniowski et al. 2019
Iglica cf. gracilis (Clessin, 1882)	MH720988/MH721003	Hofman et al. 2018
*Iglica hellenica* Falniowski & Sarbu, 2015	KT825581/MH721007	Falniowski and Sarbu 2015/ Hofman et al. 2018
*Islamia zermanica* (Radoman, 1973)	KU662362/MG551320	Beran et al. 2016/Grego et al. 2017
*Littorina littorea* (Linnaeus, 1758)	KF644330/KP113574	Layton et al. 2014/ Neretina 2014, unpublished
*Marstoniopsis insubrica* (Küster, 1853)	AF322408/–	Falniowski and Wilke 2001
Moitessieria cf. puteana Coutagne, 1883	AF367635/MH721012	Wilke et al. 2001/Hofman et al. 2018
*Montenegrospeum bogici* (Pešić & Glöer, 2012)	KM875510/MG880218	Falniowski et al. 2014/Grego et al. 2018
*Paladilhiopsis grobbeni* Kuscer, 1928	MH720991/MH721014	Hofman et al. 2018
*Paladilhiopsis turrita* (Kuščer, 1933)	MH720992/MH721015	Hofman et al. 2018
*Pontobelgrandiella* sp. Radoman, 1978	KU497024/MG551321	Rysiewska et al. 2016/Grego et al. 2017

Abbreviations:

**AH** Aperture height;

**AOO** Area of occupancy;

**AW** Aperture width;

**BH** Height of the body whorl;

**BW** Width of the body whorl;

**CA** Aperture declination angle: Angle of aperture-elongation axis vs. the columella;

**EOO** Extent of occurrence;

**H** Shell height;

**HNHM**Hungarian Natural History Museum, Budapest, Hungary;

**ISU** Ilia State University, Tbilisi, Georgia;

**LT** Type locality;

**MNHN**Muséum national d’Histoire naturelle, Paris, France;

**NHMW** Naturhistorisches Museum Wien, Austria;

**NHMUK**Natural History Museum London, UK;

**NMBE**Naturhistorisches Museum Bern, Switzerland;

**SBMNH**SBMNH">Santa Barbara Museum of Natural History, California, USA;

**SMF**Senckenberg Museum, Frankfurt, Germany;

**W** Shell width;

**ZIN**Zoological Institute of Russian Academy of Science, St Petersburg, Russia;

**ZMH**Zoological Museum University Hamburg.

## Results

### Molecular phylogeny

**Figure 5.**
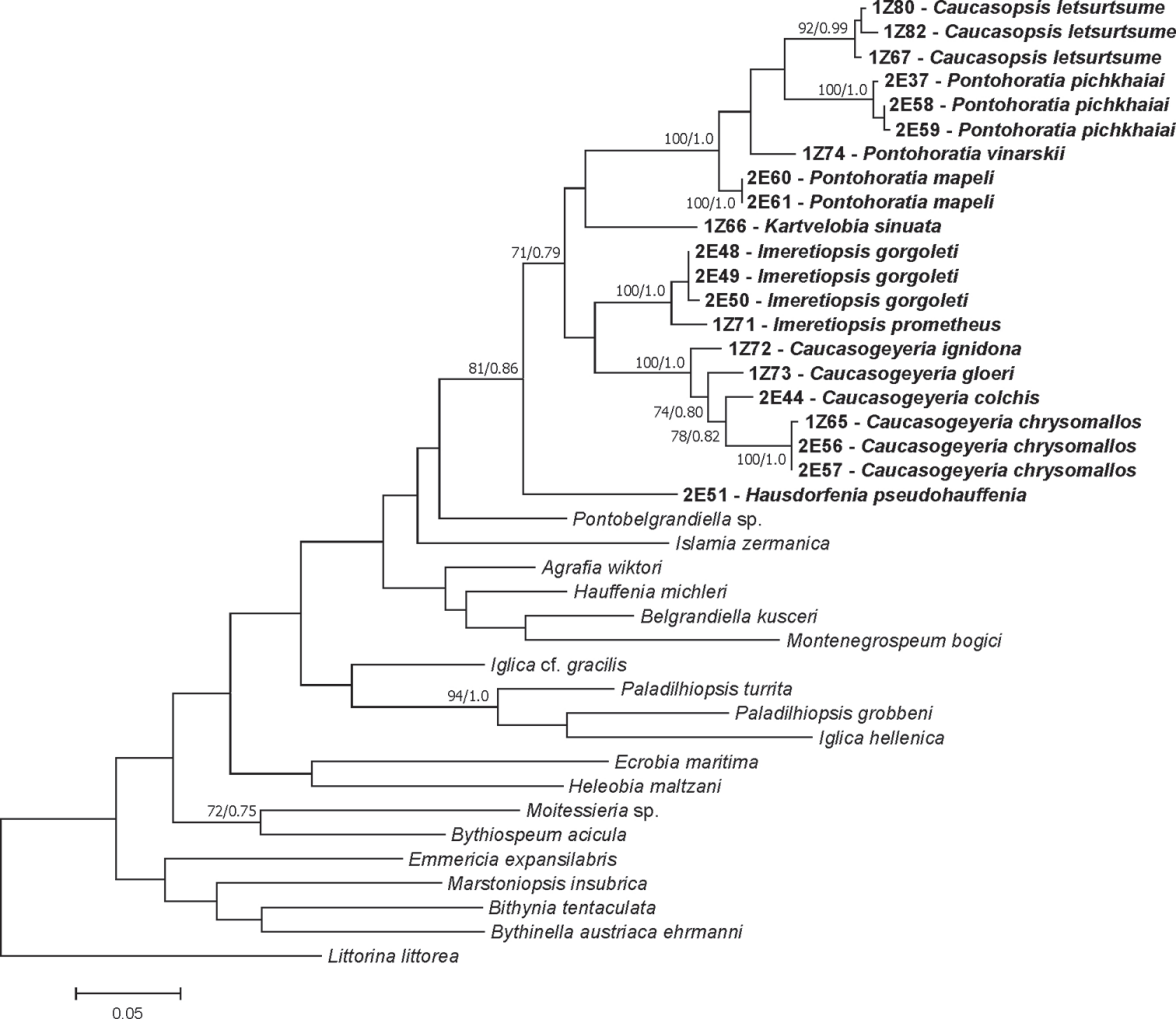
Maximum Likelihood tree inferred from mitochondrial COI. Bootstrap supports and Bayesian probabilities are given. Newly obtained sequences in bold.

**Figure 6.**
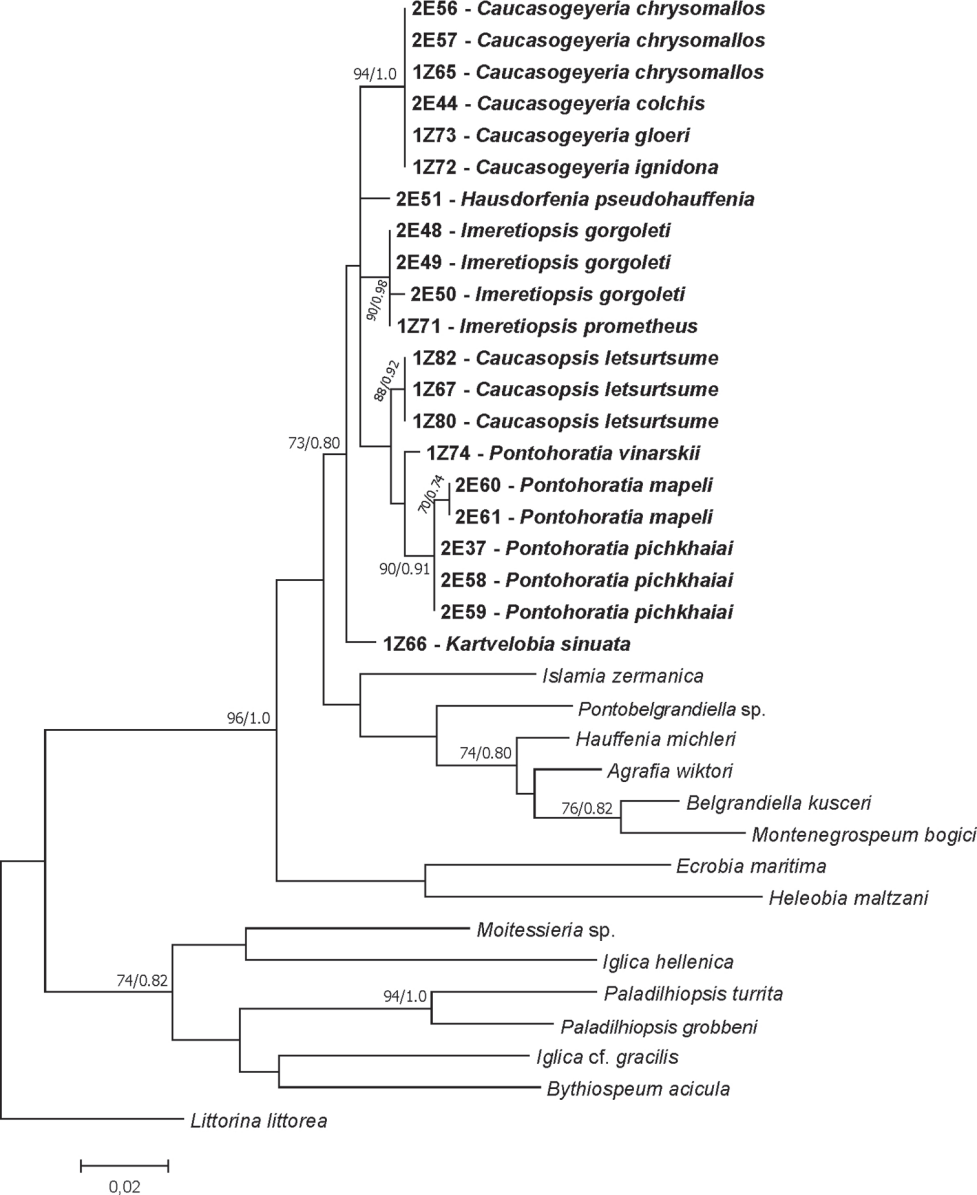
Maximum Likelihood tree inferred from nuclear H3. Bootstrap supports and Bayesian probabilities are given. Newly obtained sequences in bold.

We obtained 21 new sequences of COI (409 bp, GenBank Accession Numbers MT406082–MT406102) (Fig. 5), and 21 new sequences of H3 (309 bp, GenBank Accession Numbers MT410508–MT410528) (Fig. 6). The tests by Xia et al. (2003) for COI and H3 revealed no saturation. Phylograms were constructed for COI, H3 and for combined COI+H3 dataset. In all analyses, the topologies of the resulting phylograms were identical in both the ML and BI.

**Figure 7.**
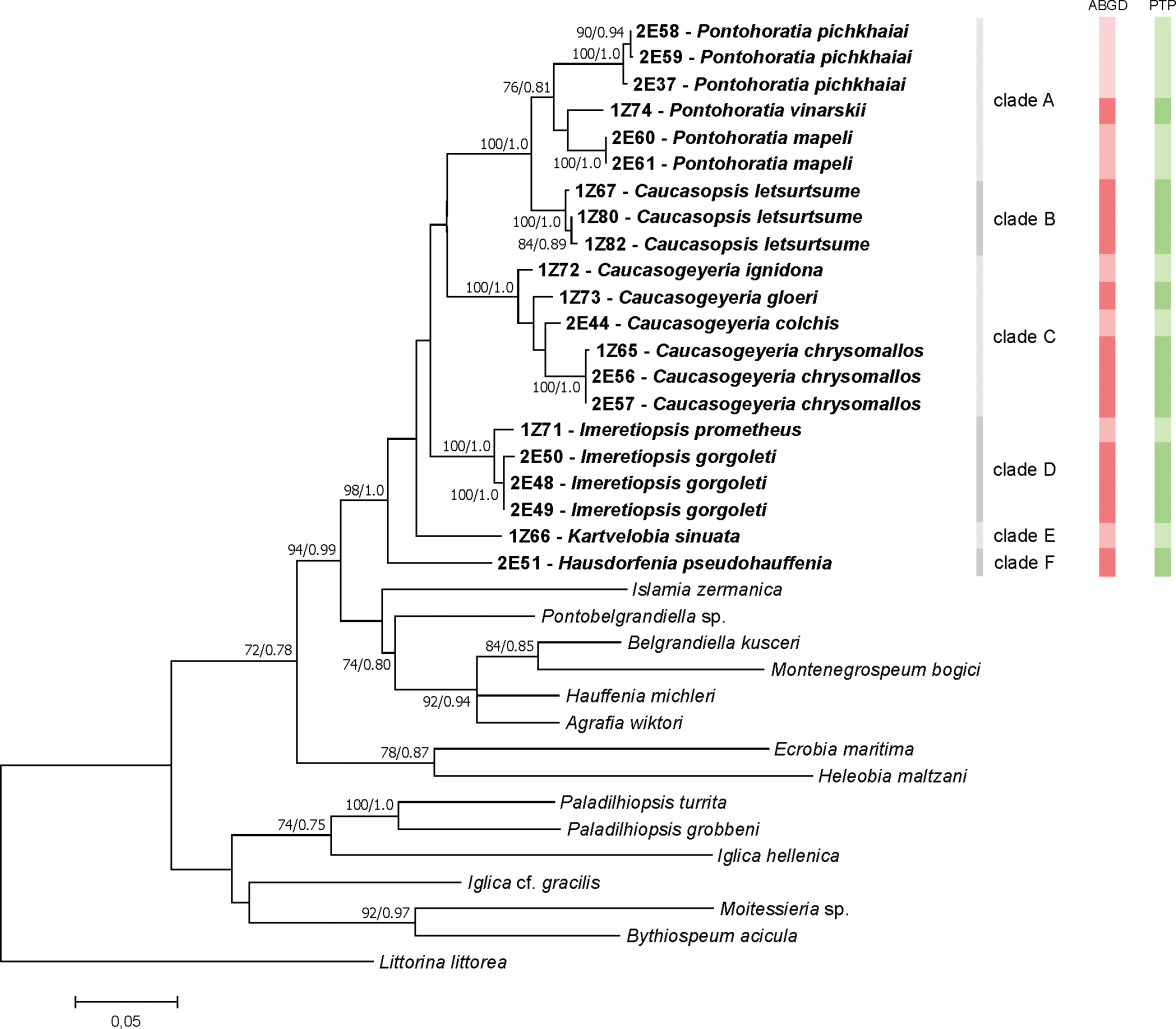
Maximum Likelihood tree inferred from COI + H3. Bootstrap supports and Bayesian probabilities are given. Red and green bars indicate results from the ABGD and PTP methods, respectively. Newly obtained sequences in bold.

In all three cases (COI, H3, and COI+H3), the newly obtained sequences formed well-supported distinct lineage, with closest relation to the subfamily Sadlerianinae. For COI most phylogeny relationships were unresolved, since the low bootstrap supports are typical for deep nodes inferred with COI. Fortunately, the tree for COI+H3 gave a clear phylogeny of the new species. This ‘georgian’ clade consisted of six subclades (Figs 5–7), representing most probably six distinct genera. The inter-genus p-distances for COI and H3, and intra-genus for COI are given in Table 2. Intra-genus p-distances for H3 are not shown due to very low or lack of variation. For both studied loci, clades A (*Pontohoratia*) and B (*Caucasopsis*) were most similar (p-distance 0.074 and 0.014 for COI and H3, respectively: Table 2). The other subclades also formed distinct lineages, but the relationships among them are not clear due to low bootstrap and BP supports. The p-distances among these four clades (C – *Caucasogeyeria*, D – *Imeretiopsis*, E – *Kartvelobia*, and F – *Hausdorfenia*) varied from 0.098 to 0.132 (for COI) and from 0.014 to 0.020 (for H3).

**Table 2.** P-distances between main clades for COI (below diagonal) and H3 (above diagonal); intra-genus p-distances for COI are also shown if present (in bold).

	* Pontohoratia *	* Caucasopsis *	* Caucasogeyeria *	* Imeretiopsis *	* Kartvelobia *	* Hausdorfenia *
* Pontohoratia *	**0.055**	0.014	0.027	0.025	0.027	0.024
* Caucasopsis *	0.074	**0.005**	0.020	0.018	0.020	0.017
* Caucasogeyeria *	0.130	0.107	**0.035**	0.018	0.020	0.017
* Imeretiopsis *	0.119	0.113	0.099	**0.014**	0.018	0.014
* Kartvelobia *	0.130	0.117	0.113	0.098	–	0.017
* Hausdorfenia *	0.148	0.137	0.132	0.121	0.112	–

**Table 3.** Measurement comparisons of *Kartvelobia sinuata* sp. nov. from different localities.

*Genus species*	H	W	BH	BW	AH	AW	CA	H/W	AH / AW	W / BW	H/BH	H/AH	W / AW	H/(W- BW)
	mm	mm	mm	mm	mm	mm	deg.							
*Kartvelobia sinuata* sp. nov. **Holotype**LT	1.67	0.96	1.16	0.81	0.76	0.61	38	1.74	1.25	1.19	1.43	2.20	1.58	11.00
*Kartvelobia sinuata* sp. nov. **Paratype**LT	1.62	0.91	1.16	0.76	0.68	0.61	36	1.78	1.13	1.20	1.39	2.37	1.50	10.67
	1.77	0.96	1.16	0.86	0.71	0.56	35	1.84	1.27	1.12	1.52	2.50	1.73	17.50
	1.72	1.01	1.11	0.81	0.76	0.61	36	1.70	1.25	1.25	1.55	2.27	1.67	8.50
	1.82	1.06	1.16	0.91	0.81	0.71	43	1.71	1.14	1.17	1.57	2.25	1.50	12.00
*Kartvelobia sinuata* sp. nov. Okatse Spring	2.07	1.11	1.26	0.86	0.81	0.66	38	1.86	1.23	1.29	1.64	2.56	1.69	8.20
Kartvelobia cf. sinuata sp. nov. Motena Cave	1.87	1.06	1.31	0.86	0.76	0.76	44	1.76	1.00	1.24	1.42	2.47	1.40	9.25
	1.77	0.96	1.21	0.83	0.73	0.63	40	1.84	1.16	1.15	1.46	2.41	1.52	14.00
Kartvelobia cf. sinuata sp. nov. Priveli Balda spring	1.79	1.16	1.09	0.86	0.76	0.66	42	1.54	1.15	1.35	1.65	2.37	1.77	5.92
	1.87	1.01	1.26	0.83	0.81	0.66	45	1.85	1.23	1.21	1.48	2.31	1.54	10.57
*Kartvelobia sinuata* sp. nov. Turchu Gamosadivari	1.62	0.91	1.11	0.81	0.71	0.58	43	1.78	1.22	1.13	1.45	2.29	1.57	16.00
*Kartvelobia sinuata* sp. nov. Nakriduri 1 spring	1.62	0.86	1.06	0.78	0.71	0.58	38	1.88	1.22	1.10	1.52	2.29	1.48	21.33
*Kartvelobia sinuata* sp. nov. Nakriduri 2 spring	1.46	0.78	0.98	0.71	0.66	0.48	35	1.87	1.37	1.11	1.49	2.23	1.63	19.33
*Kartvelobia sinuata* sp. nov. Nakriduri 2 spring	1.36	0.96	0.91	0.81	0.71	0.66	23	1.42	1.08	1.19	1.50	1.93	1.46	9.00

Both species delimitation methods (PTP and ABGD; Fig. 7) distinguished twelve new species described below, including four within *Caucasogeyeria*, three within *Pontohoratia*, and two within *Imeretiopsis*.

## Taxonomic accounts

### Class: Gastropoda Cuvier, 1795

**Clade Littorinimorpha Golikov & Starobogatov, 1975**

**Superfamily Truncatelloidea Gray, 1840**

**Family Hydrobiidae Stimpson, 1865**

**Subfamily Sadlerianinae Szarowska, 2006**

#### Genus Kartvelobia

Taxon classificationAnimaliaLittorinimorphaHydrobiidae

Grego & Mumladze gen. nov.

0BF83733-40C2-5EE4-B298-C7FBCA54668B

http://zoobank.org/21F0C1AB-8EA0-49BF-A84F-592032DD2D85

##### Type species.

*Kartvelobia sinuata* Grego & Mumladze, sp. nov.

##### Species assigned to the genus.

*Kartvelobia sinuata* Grego & Mumladze, sp. nov., *K. kinchkha* Grego & Mumladze, sp. nov., *K. shishaensis* Grego & Mumladze, sp. nov.

##### Diagnosis.

The new genus differs from all known stygobiotic Hydrobiidae and Moitessieriidae by general shell shape with characteristically deeply sinuated labral margin; however, the smaller representatives of the genus can possess only very weak labral sinuation while still having elongate-oval shells with inflated whorls and aperture slightly detached from the body whorl.

##### Etymology.

Name derived from the name of Georgia in local language Sakartevelo (საქართველო), which is frequently used in its short vocative form as Kartvelo (ქართველო). Its gender is feminine.

##### Distribution.

**Figure 8.**
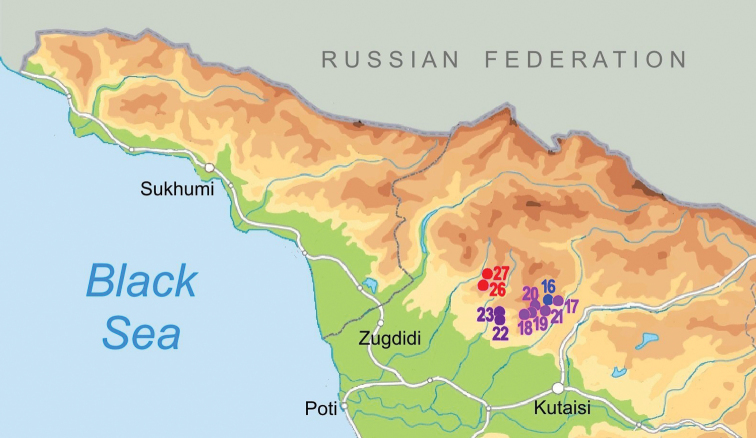
Distribution map of *Kartvelobia* gen. nov. **16***K. kinchkha* sp. nov. (blue dot) **17–21***K. sinuata* sp. nov. (magenta dots) **22, 23**K. cf. sinuata (purple dots) **26, 27***K. shishaensis* sp. nov. (red dots).

The new genus is known from western Imereti region, where it can be found in springs and caves in the Turchu Gamosadivari basin and around the karstic Pakhe Plateau. In the Samegrelo region it is distributed in springs and caves on the eastern slope of Pakhe Plateau and from the springs around Mukhuri village (Fig. 8).

#### Kartvelobia sinuata

Taxon classificationAnimaliaLittorinimorphaHydrobiidae

Grego & Mumladze sp. nov.

E0FF0A34-80B7-5B4E-A2C5-63FDA068149B

http://zoobank.org/86E484BD-58E7-45E4-84FC-DB969532B845

**Plate 1.**
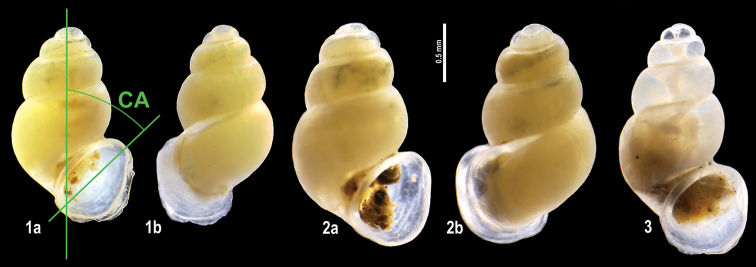
**1–3***Kartvelobia sinuata* sp. nov. Imereti, Satsiskvilo, Turchusmtha, specimens used for molecular and anatomical study. Measurement of aperture declination angle (CA): aperture-elongation axis vs. the columella is depicted in green. The numbers correspond to individuals, and the letters represent the different views of the same individual. Photograph A. Falniowski.

**Figure 9.**
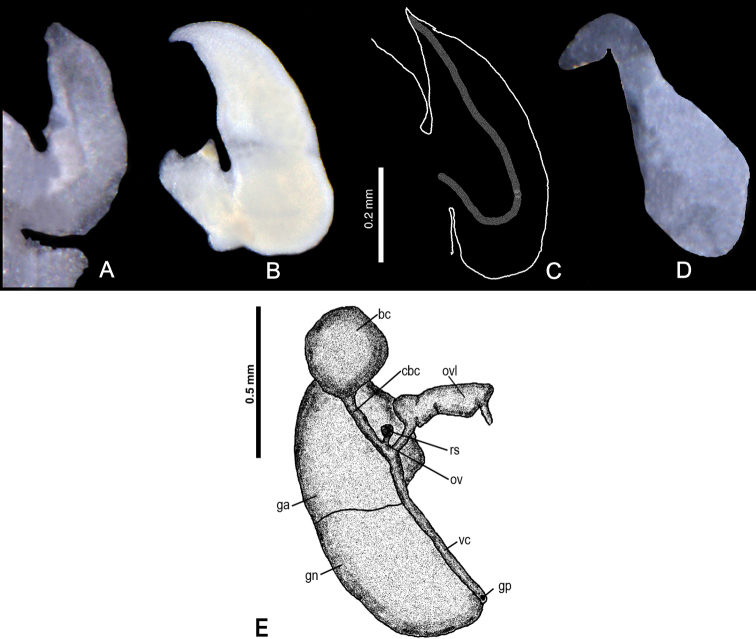
*Kartvelobia sinuata* sp. nov., Imereti, Satsiskvilo, Turchusmtha, morphology of the reproductive organs **A–D** morphology of the penis **E** female renal and pallial section of reproductive organs. Bc – bursa copulatrix; cbc – duct of bursa copulatrix; ga – albuminoid gland; gn – nidamental gland; gp – gonoporus; ov – oviduct; ovl – loop of oviduct; rs – receptaculum seminis; vc – ventral channel. Photograph and drawing A. Falniowski.

Plates 1 (1–3); 2(1–15); 3(1–4); 4(5–7); 5(3); Fig. 9A–E

##### Type locality.

Georgia • Imereti, Satsiskvilo, Turchusmtha (თურჩუსმთა, სოფელი საწისქვილო); 42°29'49"N, 42°32'49"E; 980 m a.s.l.; Small spring at left side of path to the plateau.

##### Material.

***Holotype***: Georgia • 1 adult, dry; type locality; 02 May 2018; J. Grego, L. Mumladze and M. Olšavský leg.; ISU FM-T020-H. ***Paratypes***: Georgia • same as for holotype; ISU FM-T020-P1/500 dry specimens, SBMNH 633041/7 dry, NHMW 113374/10 dry, HNHM 104683/10 dry, NHMUK 20191339/10 dry, NHMB 563971/10 dry, SMF 358930/10 dry, ZMH 140635/10 dry, MNHN-IM-2016-7894, ZIN 1/511-2020/10 dry, coll. JG F0989/500 dry, coll. Glöer/2 dry; ***Other material***: Georgia • Imereti, Nakhriduri, Turchu Gamosadivari Cave; 42°29'13"N, 42°31'20"E; 951 m a.s.l.; 03 May 2018; J. Grego, L. Mumladze and M. Olšavský leg., JG/3 dry. • Imereti, Nakhriduri 3 spring at Turchu Gamosadivari Basin left side; 42°28'41"N, 42°30'45"E; 860 m a.s.l.; 03 May 2018; J. Grego, L. Mumladze and M. Olšavský leg. JG/5 dry. • Imereti, Nakhriduri 2, Turchu Gamosadivari Basin left side spring above small ford; 42°28'39"N, 42°30 43"E; 860 m a.s.l.; 03 May 2018; J. Grego, L. Mumladze and M. Olšavský leg., JG/6 dry. • Imereti, Kinchkhaperdi (კინჩხაფერდი), spring right along the road to Askhi Plateau; 42°30'7"N, 42°33'34"E; 880 m a.s.l.; 02 May 2018; J. Grego, L. Mumladze and M. Olšavský leg. JG/3 dry. • Imereti, Upskhero Spring Lake (უფსკერო ტბა) at Turchu Gamosadivari Basin; 42°27'47"N, 42°30'3"E; 890 m a.s.l.; 03 May 2018; J. Grego, L. Mumladze and M. Olšavský leg. JG/6 dry. • Imereti, Turchusmtha, spring of Okatse above Kinchkha waterfall; 42°29'49"N, 42°32'49"E; 1050 m a.s.l.; 02 May 2018; J. Grego, L. Mumladze and M. Olšavský leg. JG/5 dry.

**Plate 2.**
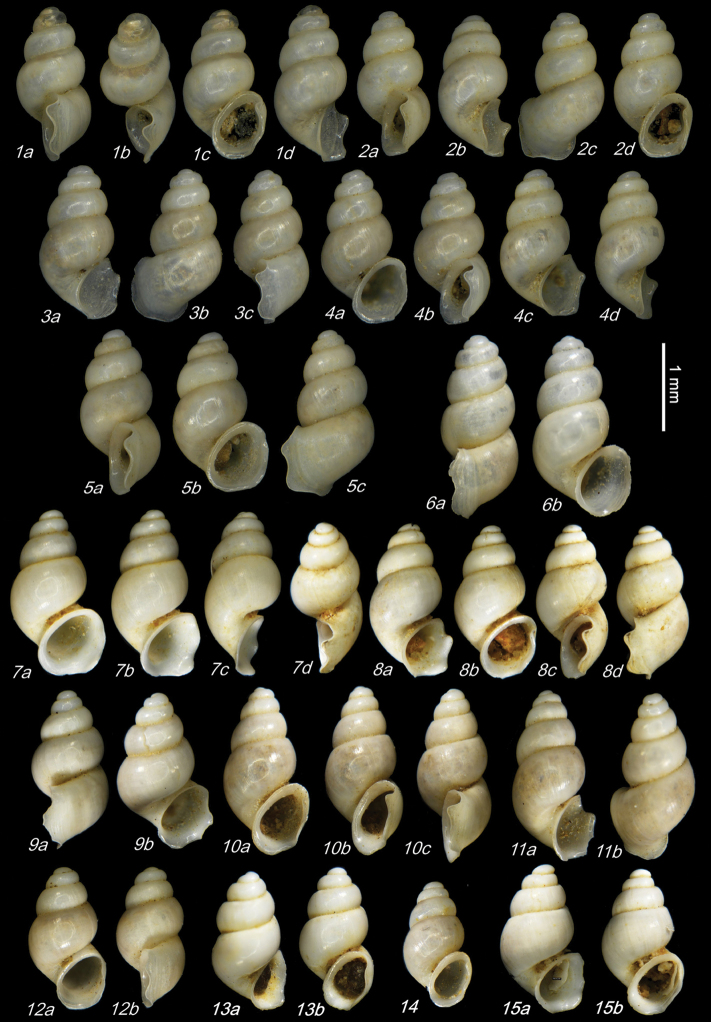
**1–5***Kartvelobia sinuata* sp. nov., Imereti, Satsiskvilo, Turchusmtha **1** holotype **2–5** paratypes **6***K. sinuata* sp. nov., Imereti, Turchusmtha, spring of Okatse above Kinchkha waterfall **7, 8**K. cf. sinuata, Samegrelo, Pirveli Balda, Motena Cave **9–11**K. cf. sinuata, Samegrelo, Pirveli Balda, spring in village **12***K. sinuata* sp. nov., Imereti, Nakhriduri, spring cave Turchu Gamosadivari **13***K. sinuata* sp. nov., Imereti, Nakhriduri spring at Turchu Gamosadivari Basin **14, 15***K. sinuata* sp. nov., Imereti, Nakhriduri side spring at Turchu Gamosadivari Basin. The numbers correspond to individuals, and the letters represent the different views of the same individual. Photograph J. Grego.

**Plate 3.**
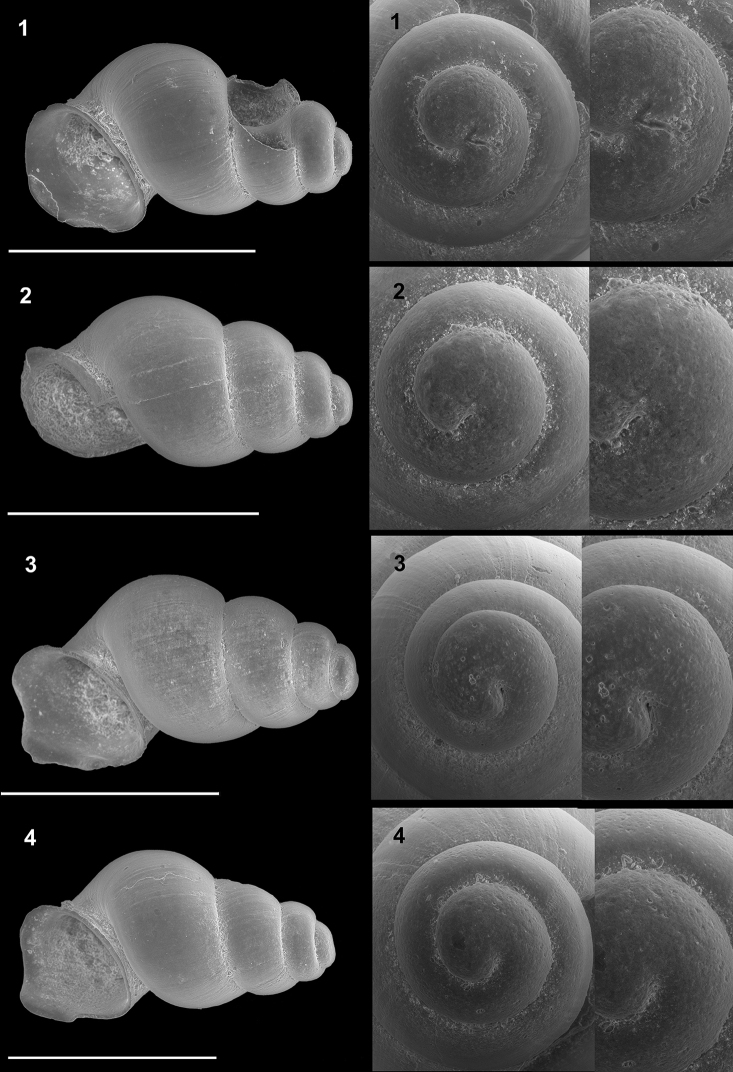
*Kartvelobia sinuata* sp. nov. **1** Imereti, Turchu Gamosadivari Basin, Nakiduri spring, paratype SBMNH 633100 **2***K. sinuata* sp. nov., Imereti, Turchu Gamosadivari Basin, Nakhriduri 5, paratype SBMNH 633103 **3***K. sinuata* sp. nov., Samegrelo, Pirveli Balda, Motena Cave, paratype SBMNH 633043 **4***K. sinuata* sp. nov., Samegrelo, Pirveli Balda, spring in village, paratype SBMNH 633070. SEM SBMNH Vanessa Delnavaz. Scale bars: 1 mm.

#### K. cf. sinuata

Taxon classificationAnimaliaLittorinimorphaHydrobiidae

2A1F5996-98F7-5C50-BEBF-74AE850C81A3

##### Other material.

Georgia • Imereti, Samegrelo, Pirveli Balda, Motena Cave (მოტენას მღვიმე), sandy sediment at terminal sump lake; 42°28'36"N, 42°23'29"E; 485 m a.s.l.; 09 May 2018; J. Grego, L. Mumladze and M. Olšavský leg. • Samegrelo, Pirveli Balda (პირველი ბალდა), spring at village; 42°29'2"N, 42°23'53"E; 295 m a.s.l.; 09 May 2018; J. Grego, L. Mumladze and M. Olšavský leg. • same as preceding; 13 October 2019; J. Grego leg.

##### Diagnosis.

The new species differs from all known stygobiotic gastropods by the characteristically and deeply sinuated labral margin with two to three large tooth-like folds. The two most closely related species, *Kartvelobia kinchkha* sp. nov. and *Kartvelobia shishaensis* sp. nov., have only weakly sinuated labral margin and generally smaller shell. Compared to *K. kinchkha* sp. nov. the protoconch is smoother and to *K. shishaensis* sp. nov. it is more conspicuously pitted. Both of the latter species generally have smaller shells.

##### Description.

***Shell***: shape is ovate-conical, 1.36–2.07 mm high with four whorls separated by a deep suture, a blunt protoconch, and a closed umbilicus. Shell surface whitish, translucent, smooth to glossy, with very faint growth lines. The aperture ovate-ellipsoid with its axis declined from columella by 38° and separated from the body whorl by a gap or groove. Its labral margin characteristically sinuated with a deeply cut broad round shaped adapical sinulus, continuing with a triangular tooth-like structure curved inward, and smoothly followed by two more, similar tooth-like structures down to lower extremity of the shell. The wavy labral margin varies significantly within the species. The lateral profile of the columellar margin more-or-less straight. The protoconch surface very weakly pitted.

***Operculum***: yellowish, translucent, elongate ellipsoid, paucispiral with an excentric nucleus.

***Animal body***: milky whitish coloured, eyeless.

***Holotype measurements***: H-1.67 mm; W-0.96 mm; BH-1.16 mm; BW-0.81 mm; AH-0.76 mm; AW-0.61 mm; CA: 38°.

***Anatomy***: the penis (Fig. 9A–D) simple, broad and massive, proximally bent, with a small outgrowth in the middle of its left side, the vas deferens running straight. The female reproductive organs (Fig. 9E) with a short and broad oviduct loop, small distal receptaculum seminis (at the position of rs_1_ of Radoman: see Szarowska (2006)) and big spherical bursa copulatrix with a long duct.

##### Etymology.

Named after the conspicuously sinuated labral margin.

##### Habitat.

The empty shells of the new stygobiotic species were found in the sandy sediments of several cave streams or karst spring heads. Few live individuals were found in a small concrete basin built on a small permanent spring emerging from a fissure in the thick limestone beds. The individuals of this hypogean species were washed out from its stygobiont habitat and accumulated in the small artificial basin.

##### Distribution.

This species is known from the Pakhe karstic plateau NW of Kinchkhaperdi and Satsiskvilo (south of the Askhi Plateau) in the caves and springs emerging from cliffs at its foot and slopes, as well from the springs and caves at Turchu Gamosadivari Basin situated at the top of the plateau the Turchu Gamosadivari River sink at the western edge of the basin, and appearing again in First Toba Cave and in Arsen Okrojanashvili Cave. A more conical form of the new species with slightly different labral margin (K. cf. sinuata is known from the southernmost tip of the Pakhe Plateau massif, from the springs in village Pirveli Balda and from Motena Cave. A local form with minute shell, inflated whorls is found around Kinchkhaperdi below the NW foot of the plateau. The taxonomic status of both forms should be clarified.

##### Conservation status.

The number of known locations is 11 and EOO is ca. 70 km^2^. The AOO is represented by only several underground karst conduits with much smaller total area compared to EOO. Each karst conduit is supplied by surface water through swallow holes, where stochastic events, as human driven pollution or habitat destruction, could lead to rapid species decline or extinction. Therefore, it is assessed as Vulnerable (VU) D2.

##### Remarks.

The labral sinuation intensity can vary by specimen, especially juvenile individuals have only weakly developed sinuation.

#### Kartvelobia kinchkha

Taxon classificationAnimaliaLittorinimorphaHydrobiidae

Grego & Mumladze sp. nov.

47059666-6554-5CD2-9DB4-A566EE90E5FA

http://zoobank.org/25A99522-9ACE-4F14-B78C-01E73755595D

**Plate 4.**
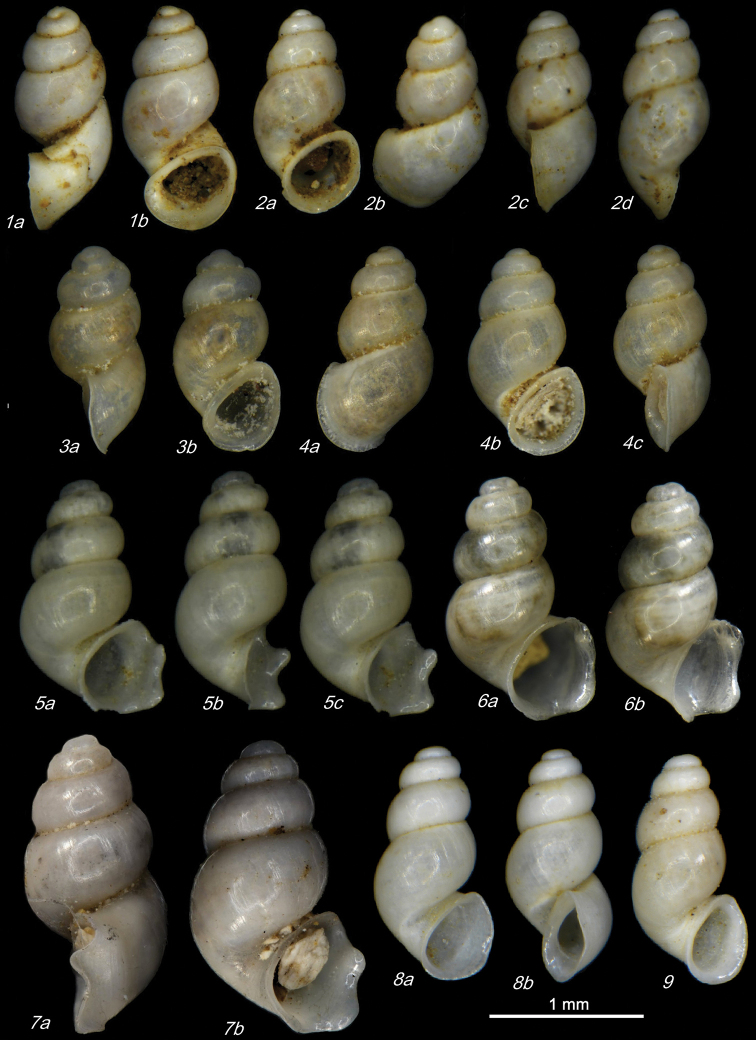
**1–4***Kartvelobia shishaensis* sp. nov., Georgia, Samegrelo, Mukhuri, Shisha Spring **1** holotype **2** paratype **3, 4***K. kinchkha* sp. nov., Imereti, Kinchkha, small travertine waterfall at right bank of Okatse below the large waterfall: **3** holotype **4** paratype **5, 6***K. sinuata* sp. nov., Imereti, Kinchkhaperdi, spring along road to Askhi Plateau, dwarf population **7***K. sinuata* sp. nov., Imereti, Satsiskvilo, Turchusmtha, paratype – typical form **8, 9**K. cf. shishaensis sp. nov., Georgia, Samegrelo, Mukhuri, Mapeli Cave. The numbers correspond to individuals, and the letters represent the different views of the same individual. Photograph J. Grego.

Plates 4 (3, 4); 5(1)

##### Type locality.

Georgia • Imereti, Kinchkhaperdi, Kinchkha; 42°29'42"N, 42°33'01"E; 855 m a.s.l.; a small spring above right edge of the lower waterfall.

##### Material.

***Holotype***: Georgia • 1 adult, dry; type locality; 02 May 2018; J. Grego, L. Mumladze and M. Olšavský leg.; ISU FM-T018-H. ***Paratypes***: Georgia • same as for holotype; ISU FM-T018-P1/1 dry, SBMNH 633106/1 dry, coll. JG F0987/1 dry.

**Plate 5.**
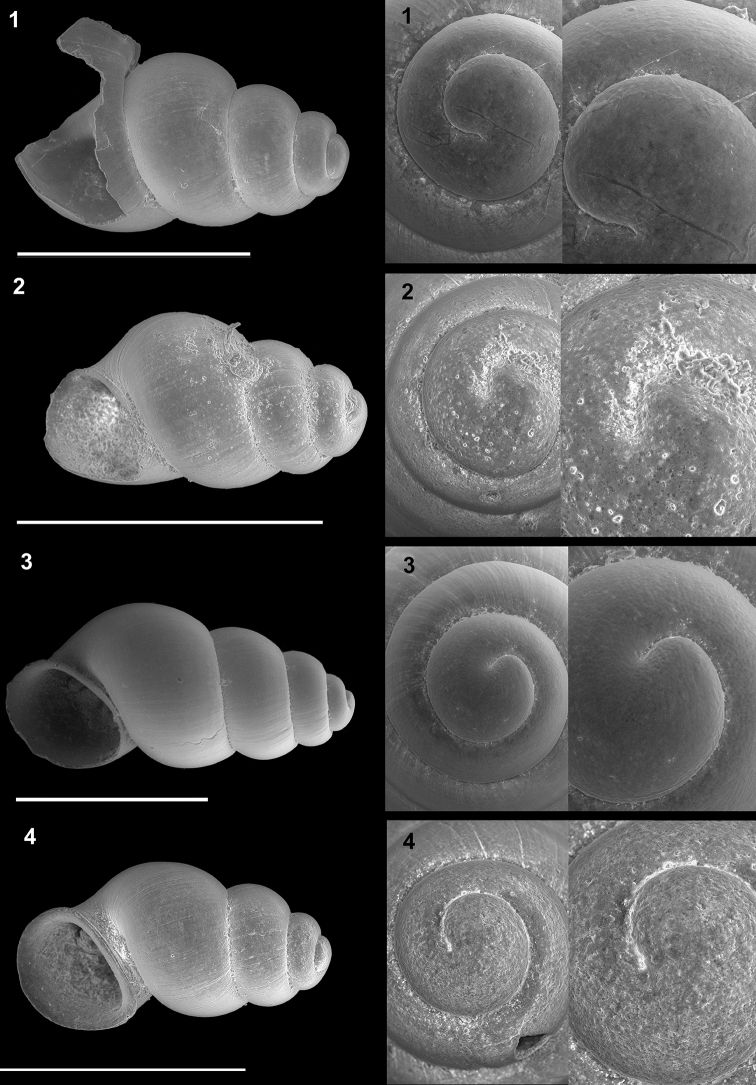
**1***Kartvelobia kinchkha* sp. nov., Georgia, Imereti, Kinschkhaperdi, paratype SBMNH 633106 **2***K. shishaensis* sp. nov., Samegrelo, Mukhuri, Shisha Spring, paratype SBMNH 633084 **3***K. sinuata* sp. nov., Imereti, Satsiskvilo, Turchusmtha Spring, SBMNH 633041 **4***K. shishaensis* sp. nov., Samegrelo, Mukhuri, Mapeli Cave, SBMNH 635905. Scale bars: 1 mm. SEM SBMNH Vanessa Delnavaz.

##### Diagnosis.

The new species differs from *Kartvelobia sinuata* sp. nov. by its less weakly-sinuated labral margin without tooth-like folds, by smaller shell size, smooth protoconch surface and the different shape of the aperture. From the similar sized *K. shishaensis* sp. nov. it differs by its more smoothly sinuated labral margin, by proportionally larger body whorl, by smoother protoconch surface and by more inflated whorls.

##### Description.

***Shell***: the minute shell (1.29–1.32 mm high) with 3½ whorls and a blunt apex, with elongate-oval shape, inflated whorls, weak suture and closed umbilicus. Shell surface smooth to glossy, whitish translucent. The aperture of an irregular tear-shaped with pronounced upper-right tip and separated from the body whorl by a deep groove. The lateral profile of labral margin characteristically weakly sinuated and anteriorly elongated. The labral columellar profile almost straight, only slightly curved. Protoconch with a smooth surface.

***Operculum***: not known.

***Animal body***: not known.

***Holotype measurements***: H-1.32 mm; W-0.71 mm; BH-0.95 mm; BW-0.62 mm; AH-0.58 mm; AW-0.52 mm; CA: 45°.

***Anatomy***: not known.

##### Etymology.

Name derived from the tallest Georgian waterfall Kinchkha (კინჩხას ჩანჩქერი) near Kinchkhaperdi. Type locality is situated between the two lower cascades of the waterfall.

##### Habitat.

Stygobiotic species. The habitat represents small permanent water springs, where the water leaks out from fissures in the large limestone beds. The water emerging from fissures could be supplied from the springs and water-episaturated zones above the Kinchkha waterfall. The very narrow fissures likely lead to evolution of the more minute shell shape of the species. Some of the small springs are captured as tap water for the nearby cabins.

##### Distribution.

Only known from the type locality.

##### Conservation status.

Number of known locations (1) fewer than 5 and AOO smaller than 20 km^2^. There is no reason to suppose that AOO, EOO, number of locations, number of subpopulations or the number or mature individuals are declining or extremely fluctuating. However, due to very small AOO it is assessed as Vulnerable (VU) D2.

#### Kartvelobia shishaensis

Taxon classificationAnimaliaLittorinimorphaHydrobiidae

Grego & Mumladze sp. nov.

A372AE2D-1BD9-5AFC-9B3B-C9240F13178B

http://zoobank.org/A96A5432-AC26-460A-BA9F-AF01A8B63BDF

Plates 4 (1, 2, 8, 9); 5(2, 4)

##### Type locality.

Georgia • Samegrelo, Mukhuri, Shisha Spring; 42°37'47"N, 42°11'26"E; 250 m a.s.l.; sediment from the spring lake bottom.

##### Material.

***Holotype***: Georgia • 1 adult, dry; type locality; 42°37'47"N, 42°11'26"E; 250 m a.s.l.; 10 May 2018; J. Grego, L. Mumladze and M. Olšavský leg.; ISU FM-T019-H. ***Paratypes***: Georgia • same as for holotype; ISU FM-T019-P1/3 dry, SBMNH 633084/2 dry, coll. JG F1043/3 dry; • same as for holotype; 12 October; 2019; J. Grego, L. Mumladze and G. Bananashvili leg.; NHMW 113373 ISU-T019-P2/4 dry, coll JG F1056/4 dry; Georgia • Samegrelo, Mukhuri, Mapeli Cave (მაპელის მღვიმე); 42°38'22"N, 42°11'39"E; 330 m a.s.l.; 12 October 2019; J. Grego, L. Mumladze and G. Bananashvili leg.

##### Diagnosis.

The new species differs from the *Kartvelobia sinuata* sp. nov. by its very weakly sinuated almost straight labral margin, minute shell size, more pitted protoconch and different shape of the aperture. From the similar sized *K. kinchkha* sp. nov. differs by its less sinuated labral margin, by less inflated whorls and by the pitted protoconch surface. Measurement comparison of *Kartvelobia* species is given in Table 4.

**Table 4.** Measurement comparison of species from genus *Kartvelobia* gen. nov.

*Genus species*	H	W	BH	BW	AH	AW	CA	H/W	AH / AW	W / BW	H/BH	H/AH	W / AW	H/(W- BW)
	mm	mm	mm	mm	mm	mm	deg.							
*Kartvelobia shishaensis* sp. nov. **Holotype**LT	1.45	0.74	1.02	0.62	0.55	0.58	51	1.96	0.95	1.20	1.42	2.61	1.26	11.75
*Kartvelobia shishaensis* sp. nov. **Paratype**LT	1.32	0.68	0.95	0.62	0.54	0.52	48	1.95	1.03	1.10	1.39	2.46	1.29	21.50
*Kartvelobia kinchkha* sp. nov. **Holotype**LT	1.32	0.71	0.95	0.62	0.58	0.52	45	1.87	1.12	1.15	1.39	2.26	1.35	14.33
*Kartvelobia kinchkha* sp. nov. **Paratype**LT	1.29	0.77	0.92	0.65	0.58	0.49	46	1.68	1.19	1.19	1.40	2.21	1.56	10.48
*Kartvelobia sinuata* sp. nov. **dwarf** Kinchkaperdi	1.51	0.95	0.95	0.71	0.62	0.55	25	1.58	1.11	1.35	1.58	2.45	1.72	6.13
	1.60	0.98	0.98	0.71	0.66	0.55	31	1.63	1.19	1.39	1.63	2.42	1.78	5.78
*Kartvelobia sinuata* sp. nov. **Paratype**LT	1.91	1.17	1.29	0.92	0.80	0.65	27	1.63	1.23	1.27	1.48	2.38	1.80	7.75
*Kartvelobia shishaensis* sp. nov. Mapeli Cave	1.51	0.77	0.95	0.66	0.55	0.46	42	1.96	1.20	1.16	1.58	2.72	1.67	14.00
	1.48	0.74	0.92	0.62	0.58	0.42	34	2.00	1.41	1.20	1.60	2.53	1.78	12.00

##### Description.

***Shell***: minute, 1.32–1.45 mm high, elongated-oval shell with four whorls, semi-blunt apex and smooth whitish glossy surface; slightly inflated whorls separated by weak suture. Aperture irregularly tear-shaped, slightly expanded and detached from the body whorls by a distant grove or gap. Lateral profile of labral margin almost straight with very inconspicuous sinuation; columellar labral profile straight. Protoconch surface pitted.

***Operculum***: not known.

***Animal body***: not known.

***Holotype measurements***: H-1.45 mm; W-0.74 mm; BH-1.02 mm; BW-0.62 mm; AH-0.55 mm; AW-0.58 mm; CA: 51°.

***Anatomy***: not known.

##### Etymology.

Name after the type locality: the karst spring Shisha at southeast end of village Mukhuri.

##### Habitat.

Stygobiotic species. The empty shells of the species were washed out through the small spring lake after large water flow induced by heavy rains in May 2018. The deep spring Lake Shisha drains karstic waters from the nearby limestone massif, but likely gets a portion of its water directly from the surface through a nearby sinkhole (more opalescent water observed shortly after the heavy rain). The condition of the shells (few worn shells and many fragments) suggests its stygobiont habitat deeper than the spring head.

##### Distribution.

Only known from the type locality and from nearby Mapeli Cave in Mukhuri.

##### Conservation status.

The number of known locations (2) is no more than 5 and EOO is smaller than 20 km^2^. There is no reason to suppose that AOO, EOO, number of locations, number of subpopulations or the number or mature individuals are declining however due to its extremely small EOO we assessed as Vulnerable (VU) D2.

##### Remarks.

The second population from Mapeli Cave generally has a more elongate and conical shell with more inflated whorls. Its taxonomic position needs to be further investigated.

#### Genus Imeretiopsis

Taxon classificationAnimaliaLittorinimorphaHydrobiidae

Grego & Mumladze gen. nov.

934DA699-9F06-5F95-888E-844AC31383DD

http://zoobank.org/6DA03D4C-31F4-412B-8D68-30E9EAC228E4

##### Type species.

*Imeretiopsis prometheus* Grego & Palatov, sp. nov.

##### Species assigned to the genus.

*I. prometheus* Grego & Palatov, sp. nov., *I. gorgoleti* Grego & Mumladze, sp. nov., *I. nakeralaensis* Grego & Mumladze, sp. nov., *I. cameroni* Grego & Mumladze, sp. nov., *I. iazoni* Grego & Mumladze, sp. nov.

##### Diagnosis.

The general shell morphology of the new genus is similar to some stygobiotic genera from the Balkans (*Paladilhiopsis* Pavlović, 1913; *Iglica* A. J. Wagner, 1910), Middle Europe (*Bythiospeum* Bourguignat, 1882) and Southeast Asia (*Pseudoiglica* Grego, 2018). The main conchological difference distinguishing the new genus from *Caucasopsis* gen. nov., is the sinuated labral profile. The penis long, without the filament characteristic of *Caucasopsis*, but with two broad outgrowths on its left side.

##### Etymology.

Name is derived from the Imereti (იმერეთი) region, where the type locality and the known distribution of the genus are located. The suffix –*iopsis* refers to the resemblance to the shells of the Balkan genus *Paladilhiopsis* Pavlović, 1913. Its gender is feminine.

##### Distribution.

**Figure 10.**
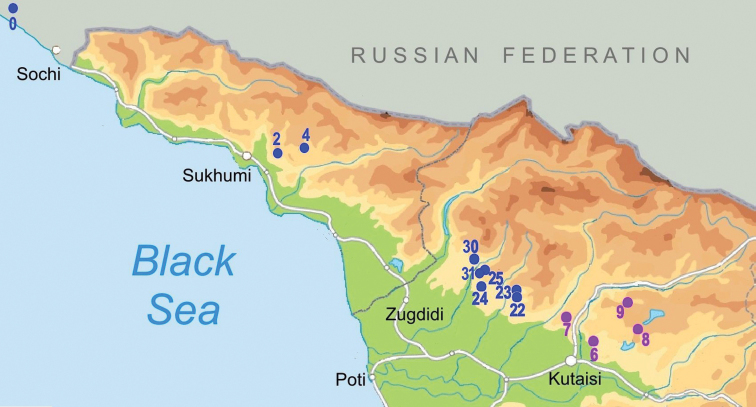
Distribution map of *Imeretiopsis* gen. nov.: (magenta dots) and *Caucasopsis* gen. nov. (blue dots) **6***I. cameroni* sp. nov. and *I. iazoni* sp. nov. **7***Imeretiopsis prometheus* sp. nov. **8***I. nakeralaensis* sp. nov. **9***I. gorgoleti* sp. nov. **0***Caucasopsis orientalis* (Starobogatov, 1962), *C. subovata* (Starobogatov, 1962) and *C. pulcherrima* (Starobogatov, 1962) **3***C. shadini* (Starobogatov, 1962) **4***C. aculeus* (Starobogatov, 1962) and *C. schakuranica* (Starobogatov, 1962) **22, 23***C. egrisi* sp. nov. **24***C. olsavskyi* sp. nov. **25, 30, 31***C. letsurtsume* sp. nov.

The genus *Imeretiopsis* gen. nov. is known from the Imereti and West Racha regions of Georgia (Fig. 10).

#### Imeretiopsis prometheus

Taxon classificationAnimaliaLittorinimorphaHydrobiidae

Grego & Palatov sp. nov.

D5DCFC9B-5836-5627-B4FB-771A06C5DCE7

http://zoobank.org/70A971CF-7229-4C46-93CC-8D31DDC65E07

**Plate 6.**
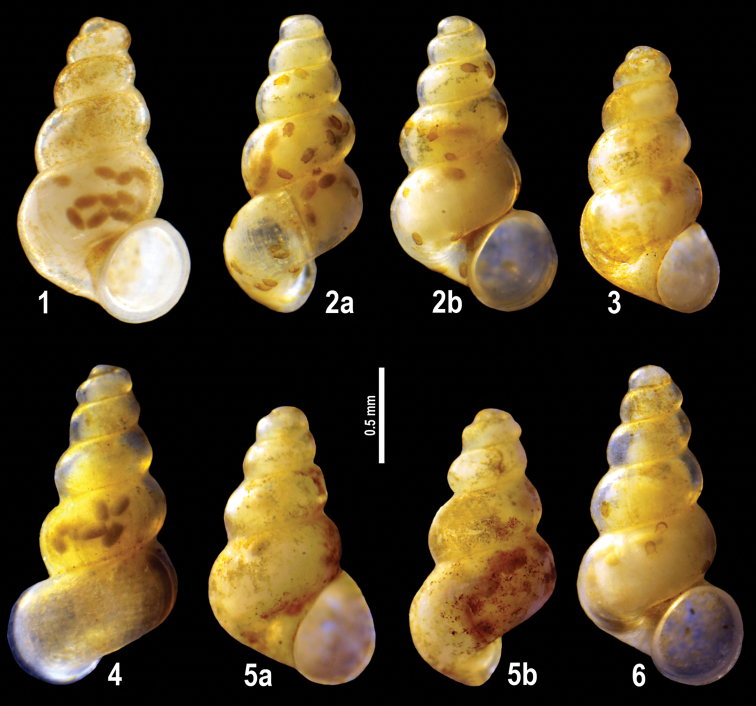
**1–6***Imeretiopsis prometheus* sp. nov., Imereti, Kumistavi, Prometheus Cave, specimens used for molecular and anatomical studies. The numbers correspond to individuals, and the letters represent the different views of the same individual. Photograph A. Falniowski.

Plates 6 (1–6); 7(1); 9(7)

##### Type locality.

Georgia • Imereti, Kumistavi, Prometheus Cave (პრომეთეს მღვიმე); 42°22'33"N, 42°36'2"E; 175 m a.s.l.; bottom of cave stream.

##### Material.

***Holotype***: Georgia • 1 adult, dry; type locality; 01 May 2018; J. Grego, L. Mumladze and M. Olšavský leg.; ISU FM-T017-H. ***Paratypes***: Georgia • same as for holotype; ISU FM-T017-P1/21 dry, SBMNH 633098/4 dry, NHMW 113372/1 dry, HNHM 104682/1 dry, NHMUK 20191338/1 dry, NHMB 563970/1 dry, SMF 358929/1 dry, ZMH140634/1 dry, NHMW 113372/1 dry, ZIN 1/508-2020/1 dry, coll. JG F/21 dry and 2 wet, coll. Glöer/1 dry.

##### Diagnosis.

The species differs from all the related morphotypes from the Caucasus by the more conical-elongate shell with typical triangular shell shape, by the more oval aperture situated more right of the columellar axis (to viewer; shell in apertural pose, apex up). *I. cameroni* sp. nov. has a much narrower elongated shell shape with a more elongated aperture and less inflated whorls with closed umbilicus, and *I. nakeralaensis* sp. nov. has more robust, oval shell with proportionally smaller aperture and narrower umbilicus.

##### Description.

***Shell***: elongate-conical, 1.42–1.66 mm high with five whorls, blunt protoconch, inflated whorls, deep suture and proportionally larger body whorl. Umbilicus narrow, slit like. Shell surface glossy, translucent with horny-yellowish periostracum, occasionally covered by rusty-brown inorganic incrustations. Aperture irregularly ovate, slightly expanded, separated from body whorl by a grove and by broadening adapical apertural gap. Lateral labral profile weakly sinuated, columellar profile straight. Protoconch strongly pitted.

***Operculum***: yellowish, translucent, elongate ellipsoid, paucispiral with excentric nucleus.

***Animal body***: eyeless, milky whitish coloured with light brown pellets.

***Holotype measurements***: H-1.66 mm; W-0.53 mm; BH-0.87 mm; BW-0.72 mm; AH-0.60 mm; AW-0.47 mm; CA: 33°.

##### Etymology.

Name is derived from the type locality inside Prometheus Cave (პრომეთეს მღვიმე). The cave was named after Prometheus, the Titan of Greek mythology, who created mankind from clay, stealing the fire from gods and providing it to humanity. As punishment, he was eternally bound to a rock at Caucasus Mountains, where each day an eagle was sent to feed on his liver.

##### Habitat.

Stygobiotic species. Empty shells of the new species were found among the sandy sediments inside the cave stream of Prometheus Cave. Live individuals were found attached at the slimy surface of boulders and gravel at the bottom of underground streambed. The rock surface was covered by dark brown- black slimy microbial mats likely serving as a food substrate. More specimens were found in flowing stream than semi-stagnant water.

##### Distribution.

Only known from the type locality.

##### Conservation status.

The number of known localities (1) is no more than 5 and EOO is smaller than 20 km^2^. There is no reason to suppose that AOO, EOO, number of locations, number of subpopulations or the number or mature individuals are declining however due to its extremely small EOO we assessed as Vulnerable (VU) D2.

**Plate 7.**
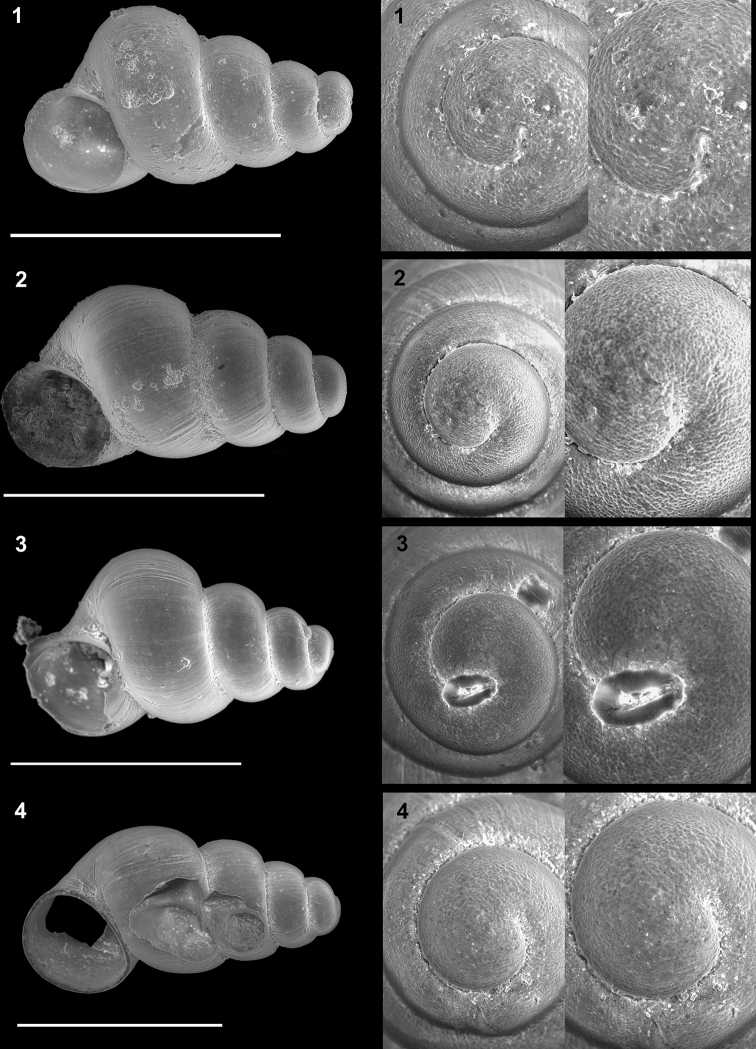
**1***Imeretiopsis prometheus* sp. nov., Imereti, Kumistavi, Prometheus Cave, paratype SBMNH 633098 **2***I. nakeralaensis* sp. nov., Imereti, Tikbuli, spring below Nakerala Pass, paratype SBMNH 633110 **3***I. gorgoleti* sp. nov., Racha, Gorgoleti, above road to Tsakhi, paratype SBMNH 635910 **4***Caucasopsis egrisi* sp. nov., Pirveli Blada, spring in village, paratype SBMNH 639559. Scale bars: 1 mm. SEM SBMNH Vanessa Delnavaz.

#### Imeretiopsis gorgoleti

Taxon classificationAnimaliaLittorinimorphaHydrobiidae

Grego & Mumladze sp. nov.

6082F8E2-7597-5B29-9F24-A32B5E91899A

http://zoobank.org/9326015A-DFD6-4CE8-BFE4-4A35FBFC981C

**Figure 11.**
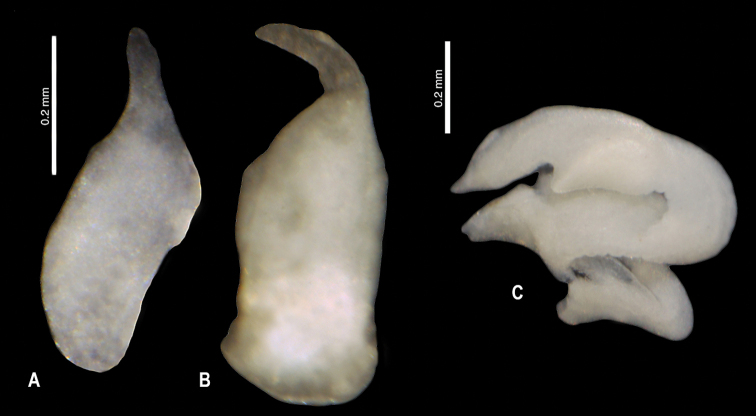
Morphology of the penis **A, B***Caucasopsis letsurtsume* sp. nov., Samegrelo: **A** ventrally **B** dorsally **C***Imeretiopsis gorgoleti* sp. nov. Photograph A. Falniowski and A. Rysiewska.

Plates 7 (3); 8(1–4, 9–14); Fig. 11C

##### Type locality.

Georgia • Racha, Gorgoleti; 42°31'03"N, 42°54'59"E; 620 m a.s.l.; small cave spring on the right bank of the Shareula River between Gorgoleti and Tsakhi villages.

**Plate 8.**
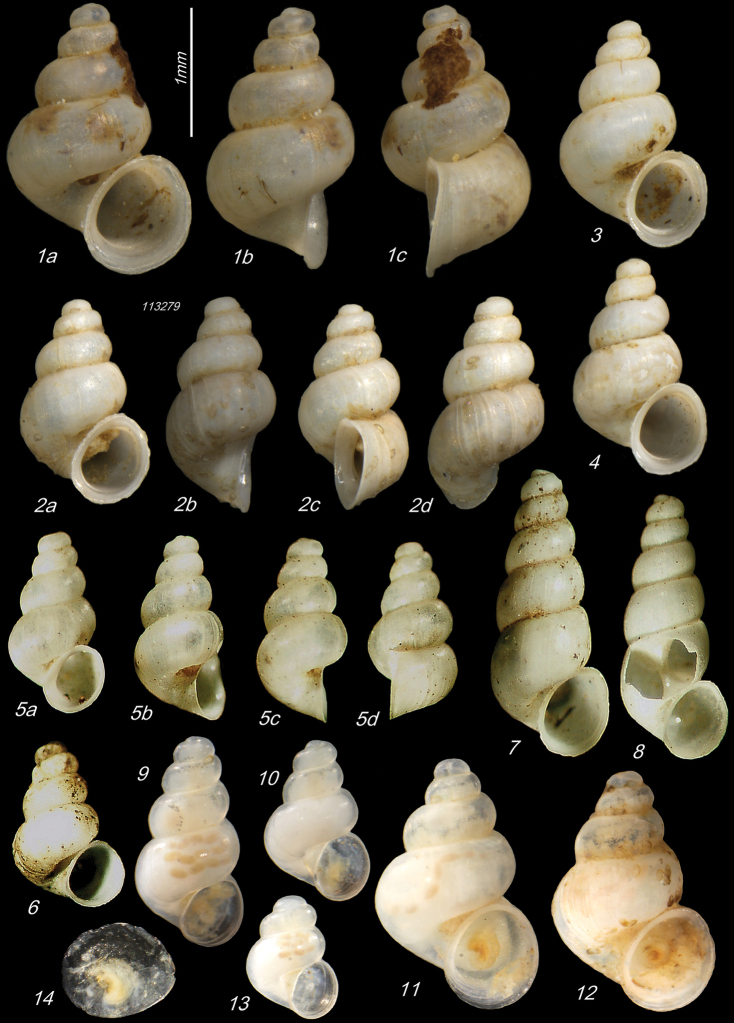
**1–4***Imeretiopsis gorgoleti* sp. nov., Racha, Gorgoleti, small spring above the road to Tsakhi: **1** holotype **2** paratype NHMW113279 **3, 4** paratypes **5, 6***I. iazoni* sp. nov., Imereti, Kutaisi, Iazoni Cave: **5** holotype **6** paratype **7, 8***I. cameroni* sp. nov., Imereti, Kutaisi, Iazoni Cave, paratypes **9–13***I. gorgoleti* sp. nov., specimens used for molecular and anatomical studies **14***I. gorgoleti* sp. nov., operculum. The numbers correspond to individuals, and the letters represent the different views of the same individual. Photograph Sara Schnedl, NHMW, and A. Rysiewska.

##### Material.

***Holotype***: Georgia • 1 adult, dry; type locality; 13 October 2019; J. Grego, L. Mumladze and G. Bananashvili; ISU FM-T013-H. ***Paratypes***: same as for holotype; ISU FM-T013-P1/49 dry and 83 wet, SBMNH 635910/7 dry, NHMW 113279/2 dry, HNHM 104681/2 dry, NHMUK 20191337/2 dry, NHMB 563968/2 dry, SMF 358928/2 dry, ZMH 140633/2 dry, MNHN-IM-2016-7897, ZIN 1/509-2020/2 dry, coll. JG F1430/49 dry and 84 wet.

##### Diagnosis.

The new species differs from all the related species of the region by its more robust shape, more open umbilicus and more expanded rounded aperture. The most similar shell morphology can be seen in *I. iazoni* sp. nov., however, *I. gorgoleti* sp. nov. has a much larger and robust shell with a more open umbilicus and more expanded aperture. From the members of the genus *Caucasopsis* gen. nov. as the *C. tsurtsume* sp. nov. it differs by its less sinuated labral margin and by a more regular apertural form.

##### Description.

***Shell***: height ranges from 1.52 to 2.18 mm, conical to ovate-conical shell, with 4½ whorls, blunt protoconch, rather inflated whorls and deep suture. Umbilicus widely open. Shell surface glossy, milky-translucent with very faint almost invisible axial growth lines. Aperture subcircular and expanded. Lateral labral profile weakly sinuated adapically toward the body whorl; columellar labrum has a weak sinuation near columella. Protoconch surface densely covered by large regular weak pits.

***Operculum***: translucent, milky whitish, paucispiral with excentric nucleus.

***Animal body***: animal white, eyeless with light brown pellets and randomly spread dark grey diffused fibre-like streaked blotches on mantle visible through the translucent shell from body whorl up to the early whorls.

***Holotype measurements***: H-2.18 mm; W-1.44 mm; BH-1.35 mm; BW-1.15 mm; AH-0.94 mm; AW-0.82 mm; CA: 38°.

***Anatomy***: the penis (Fig. 11C) bent, cylindrical, distally with no filament but broadly conical, in its median part a characteristically shaped double outgrowth, proximally broad and distally blunt.

##### Etymology.

Name derived from Gorgoleti village (Racha region) (სოფელი გორგოლეთ), which is the closest village to the type locality.

##### Habitat.

Stygobiotic species. Many live specimens were found on tree roots submerged in small cave ponds. The phreatic rhizosphere habitat provides enough food either directly through root exudation (Canarini et al. 2019), by direct feeding on root tissue or feeding on microbial slime covering the submerged roots, as well as on the decaying roots.

##### Distribution.

Only known from the type locality.

##### Conservation status.

The number of known localities (2) is no more than 5 and EOO is smaller than 20 km^2^. There is no reason to suppose that AOO, EOO, number of locations, number of subpopulations or the number or mature individuals are declining however due to its extremely small EOO we assessed as Vulnerable (VU) D2.

##### Remarks.

The phreatic rhizosphere habitat for gastropods was known to us from Central and South-eastern Europe. There it hosts mostly valvatiform shelled stygobiotic gastropods; however, the rich food source it provides can attract various gastropod species. We suppose the slightly sinuated aperture (labral and columellar margin) of *Imeretiopsis* could help the animals in attaching to cylindrical shape of the fine roots.

#### Imeretiopsis nakeralaensis

Taxon classificationAnimaliaLittorinimorphaHydrobiidae

Grego & Mumladze sp. nov.

1B2BA0EC-1C64-52D7-9A3D-1249E5451D1B

http://zoobank.org/38835E75-8986-46B0-9040-F427F5E12A57

Plate 9 (9)

##### Type locality.

Georgia • Imereti, Tkibuli, Tkibuli-Nikortsminda road to Nakerala Pass (ნაქერალას უღელტეხილი); 42°23'00"N, 42°00'45"E; 980 m a.s.l.; spring above left side of road with small travertine waterfall and a small spring cavern entrance.

**Plate 9.**
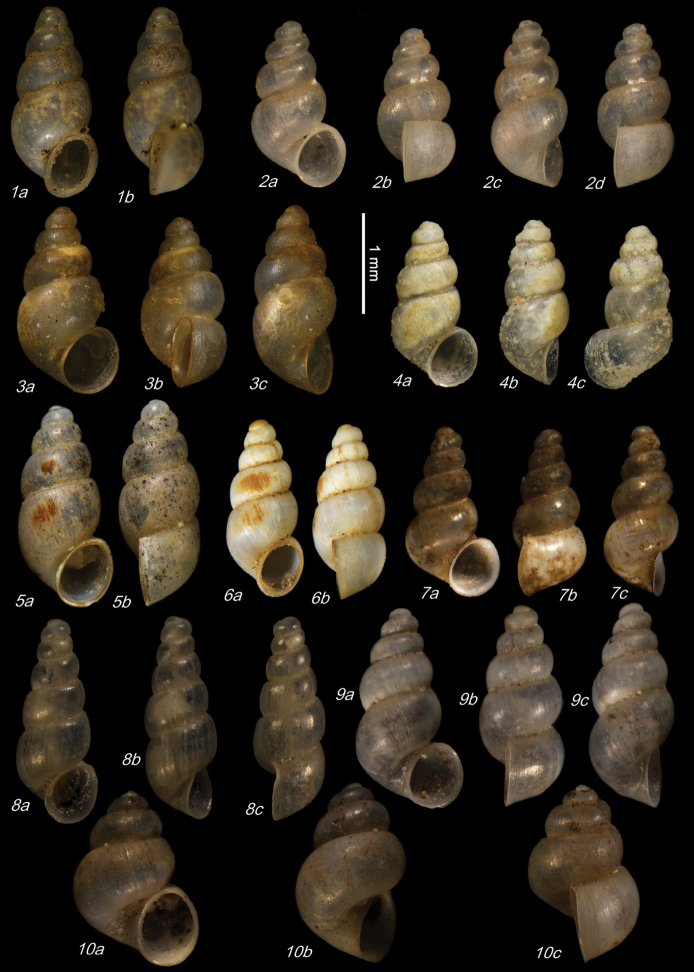
**1***Caucasopsis olsavskyi* sp. nov., Samegrelo, Chkhorotsku, Nazodelavo Cave, holotype **2***C. letsurtsume* sp. nov., Samegrelo, Chkhorotsku, Letsurtsume, Letsurtsume Cave, holotype **3***C. letsurtsume* sp. nov., Samegrelo, Chkhorotsku, Kachara Cave **4***C. egrisi* sp. nov., Samegrelo, Pirveli Balda, Motena Cave form **5, 6***C. egrisi* sp. nov., Samegrelo, Pirveli Balda, spring in village: **5** holotype **6** paratype **7***Imeretiopsis prometheus* sp. nov., Imereti, Kumistavi, Prometheus Cave, holotype **8***I. cameroni* sp. nov., Imereti, Kutaisi, Iazoni (Tskhal-Tsiteli) Cave Spring, holotype **9***I. nakeralaensis* sp. nov., Imereti, Tikibuli, spring above Tikibuli-Nikortsminda road to Nakerala pass, holotype **10***C. letsurtsume* sp. nov., Samegrelo, Chkhorotsku, Letsurtsume, Letsurtsume Cave, robust morphotype. The numbers correspond to individuals, and the letters represent the different views of the same individual. Photograph J. Grego.

##### Material.

***Holotype***: Georgia • 1 adult, dry; type locality; 04 May 2018; J. Grego, L. Mumladze and M. Olšavský leg.; ISU FM-T015-H. ***Paratypes***: Georgia • same as for holotype; ISU FM-T015-P1/10 dry, SBMNH 633110/2 dry, NHMW 113371/1 dry, NHMB 563969/2 dry, coll. JG F1009/10 dry, coll. Glöer/1 dry.

##### Diagnosis.

The new species differs from all the morphotypes with related shell shape in the region by its more robust oval shape, by the position of the aperture more right of the columella (to viewer; shell in apertural pose, apex up), and by the more open umbilicus. *Caucasogeyeria shakuranica* (Starobogatov, 1962) from Abkhazia has similar but narrower shell shape with less inflated whorls and a proportionally smaller body whorl. *Caucasogeyeria letsurtsume* sp. nov. has a smaller shell with more inflated whorls and more open umbilicus.

##### Description.

***Shell***: 2 mm high, elongate ovate-conical with pronounced protoconch, five tumid whorls and moderately deep suture. Shell surface whitish, translucent-glossy, covered by faint axial growth lines. Umbilicus open. Proportionally small aperture irregular, almost round, not expanded, with straight lateral and columellar labral profiles lacking sinuation. Protoconch surface densely and coarsely pitted.

***Operculum***: not known.

***Animal body***: not known.

***Holotype measurements***: H-2.00 mm; W-1.09 mm; BH-1.02 mm; BW-0.85 mm; AH-0.64 mm; AW-0.53 mm; CA: 29°.

***Anatomy***: not known.

##### Etymology.

Name after the Nakerala Pass 1218 m alt. situated above the type locality north of Tikbuli along the road to Ambrolauri.

##### Habitat.

Stygobiotic species. The empty shells of the species were found at the foot of small travertine cascade formed by a small stream emerging from the very narrow cave spring (small entrance covered by moss and ivy). Only a few shells were found in sparse sediments accumulated near the cave walls. The shells were washed out from its subterranean habitat by the very small permanent stream.

##### Distribution.

Only known from the type locality.

##### Conservation status.

The number of known locations (1) is no more than 5 and EOO is smaller than 20 km^2^. There is no reason to suppose that AOO, EOO, number of locations, number of subpopulations or the number or mature individuals are declining however due to its extremely small EOO we assessed as Vulnerable (VU) D2.

##### Remarks.

The assignment of the new species to the genus *Imeretiopsis* gen. nov. is only provisional, based on the resemblance if its shell to that of the type species. However, molecular data will be essential to confirm generic placement.

#### Imeretiopsis cameroni

Taxon classificationAnimaliaLittorinimorphaHydrobiidae

Grego & Mumladze sp. nov.

29048650-A832-5244-9510-B797A1CB57AF

http://zoobank.org/0E981A62-FAEF-4440-9B5F-636EA97EFBAB

Plates 8 (7, 8); 9(8)

##### Type locality.

Georgia • Imereti, Kutaisi, Iazoni (Tskhal-Tsiteli) Cave spring (იაზონის იგივე წყალ-წითელას მღვიმე), right bank of Tskalsitela River; 146 m alt., 42°16'18"N, 42°44'2"E; 145 m a.s.l.; sandy sediment inside the cave.

##### Material.

***Holotype***: Georgia • 1 adult, dry; type locality; 01 May 2018; J. Grego, L. Mumladze and M. Olšavský leg.; ISU FM-T016-H. ***Paratypes***: Georgia • same as for holotype; 12 October 2019; J. Grego, L. Mumladze and G. Bananashvili leg.; 4 ISU FM-T016-P1/1 dry, JG F1406/1 dry, ZIN 1dry.

##### Diagnosis.

The new species differs conspicuously from all the similar species of the region by its more slender shell with more conspicuous axial growth lines, closed umbilicus and narrower aperture. *Caucasogeyeria orientalis* (Starobogatov, 1962) has a similar, but more robust, oval shell shape with a different shape of the aperture.

##### Description.

***Shell***: elongate narrow-turreted, 2.00–2.29 mm high shell with 5½ tumid whorls, weak suture and flat blunt apex. The early whorls rather inflated, and the inflation of whorls regularly decreasing abapically, apex almost flat. Shell surface glossy, whitish translucent with faint regularly spaced distant rib-like growth lines. Umbilicus closed. Aperture not expanded, elongate-oval with weakly sinuated labral profile and flat columellar profile. Protoconch pitted.

***Operculum***: light yellow, horny, elongate ellipsoid, paucispiral with excentric nucleus.

***Animal body***: not known.

***Holotype measurements***: H-2.00 mm; W-0.81 mm; BH-0.96 mm; BW-0.72 mm; AH-0.62 mm; AW-0.49 mm; CA: 35°

***Anatomy***: not known.

##### Etymology.

Named after Robert A. D. Cameron from Sheffield University, who significantly contributed to the malacological knowledge of Eurasia including Caucasus region.

##### Habitat.

Stygobiotic species. The fresh empty shells, some with opercula, were found in the sandy sediment of the cave stream. The condition of the shells indicates its habitat in the deep cave zone.

##### Distribution.

Only known from the type locality.

##### Conservation status.

The species is known from a single location and EOO is smaller than 10 km^2^. There is also indication of stochastic human driven habitat pollution and a very scare occurrence of dead mature individuals indicating a very weak surviving population. Therefore, it is assessed as Critically endangered (EN) B2.

##### Remarks.

The assignment of the new species to the genus *Imeretiopsis* gen. nov. is only provisional, based on the shell habitus, e.g., the similarly sinuated lateral labral profile. Molecular data will be necessary to confirm the taxonomic position of the species. The type locality, Iazoni Cave was formed in Cretaceous limestone with a high content of quartz sand grains. The sand accumulated in thick sedimentary layers after the carbonate dissolution inside the cave. A few kilograms of the cave sand had to be screened to find a single specimen. The cave drains water from a populated area SE of Kutaisi, and the sediments indicated a contamination by micro plastic and perhaps occasionally by chemicals from municipal waste. This can pose a direct danger to the important cave fauna including *Motsametia borutzkii* (Shadin, 1932), *Euglesa subterranea* (Shadin, 1932) and cave shrimps *Xiphocaridinella kutaissiana* Sadowski, 1930, *Niphargus borutzkyi* Birstein, 1933 and *Asellus monticola fontinalis* Birstein, 1936 reported from the type locality.

#### Imeretiopsis iazoni

Taxon classificationAnimaliaLittorinimorphaHydrobiidae

Grego & Mumladze sp. nov.

28ACD379-9F45-5760-9D11-D2E37785C07B

http://zoobank.org/39F50FD0-D0AC-4DD5-9298-90CFE998515F

Plate 8 (5–6)

##### Type locality.

Georgia • Imereti, Kuatisi, Iazoni (Tskhal-Tsiteli) Cave Spring (იაზონის იგივე წყალ-წითელას მღვიმე), right bank of Tskalsitela River Canyon; 42°16'18"N, 42°44'02"E; 145 m a.s.l.; sandy sediment inside the cave.

##### Material.

***Holotype***: Georgia • 1 adult, dry; type locality; J. Grego, L. Mumladze and G. Bananashvili leg.; ISU FM-T014-H. ***Paratypes***: same as for holotype, ISU FM-T014-P1/1 dry, coll. JG F1409/1dry.

##### Diagnosis.

The species differs from the most closely related *Imeretiopsis gorgoleti* sp. nov. by its much smaller, less inflated shells with proportionally smaller and less expanded aperture and by the smaller umbilicus. From the other stygobiotic gastropods of the region with similar shell shape it differs by its smaller shell with the sinuated lateral labral profile. From the sympatric *I. cameroni* sp. nov. it differs by the much smaller shell, more inflated whorls, flatter apex and more open umbilicus. Measurement comparison *Imeretiopsis* species is given in Table 5.

**Table 5.** Measurement comparison of species from genus *Imeretiopsis* gen. nov.

***Genus species***	**H**	**W**	**BH**	**BW**	**AH**	**AW**	**CA**	**H/W**	**AH / AW**	**W / BW**	**H/BH**	**H/AH**	**W / AW**	**H/(W- BW)**
	**mm**	**mm**	**mm**	**mm**	**mm**	**mm**	**deg.**							
*Imeretiopsis gorgoleti* sp. nov. **Holotype**LT	2.18	1.44	1.35	1.15	0.9 4	0.82	38	1.51	1.15	1.25	1.61	2.32	1.76	7.52
*Imeretiopsis gorgoleti* sp. nov. **Paratype**LT	1.64	1.03	1.09	0.85	0.71	0.62	35	1.59	1.15	1.21	1.50	2.31	1.66	9.11
	1.85	1.02	1.18	0.94	0.76	0.62	30	1.82	1.23	1.08	1.57	2.43	1.64	23.72
*Imeretiopsis iazoni* sp. nov. **Holotype**LT	1.47	0.74	0.85	0.68	0.50	0.47	35	1.99	1.06	1.09	1.73	2.94	1.57	24.50
*Imeretiopsis iazoni* sp. nov. **Paratype**LT	1.35	0.88	0.82	0.68	0.50	0.41	32	1.53	1.22	1.29	1.65	2.70	2.15	6.75
*Imeretiopsis cameroni* sp. nov. **Paratype**LT	2.29	0.94	1.15	0.79	0.71	0.53	34	2.44	1.34	1.19	1.99	3.23	1.77	15.27
	2.12	0.85	1.06	0.71	0.62	0.50	32	2.49	1.24	1.20	2.00	3.42	1.70	15.14
*Imeretiopsis cameroni* sp. nov. **Holotype**LT	2.00	0.81	0.96	0.72	0.62	0.49	35	2.47	1.26	1.12	2.09	3.24	1.65	23.50
*Imeretiopsis prometheus* sp. nov. **Holotype**LT	1.66	0.53	0.87	0.72	0.60	0.47	33	3.12	1.27	0.74	1.90	2.79	1.14	-8.67
*Imeretiopsis prometheus* sp. nov. **Topotype**LT	1.63	0.85	0.88	0.75	0.55	0.48	32	1.91	1.16	1.13	1.86	2.95	1.79	16.25
	1.55	1.63	0.90	0.70	0.53	0.45	31	0.95	1.17	2.32	1.72	2.95	3.61	1.68
	1.43	0.75	0.75	0.63	0.48	0.38	32	1.90	1.27	1.20	1.90	3.00	2.00	11.40
	1.48	0.88	0.85	0.70	0.58	0.45	30	1.69	1.28	1.25	1.74	2.57	1.94	8.43
	1.70	0.88	0.88	0.73	0.53	0.46	34	1.94	1.14	1.21	1.94	3.24	1.89	11.33
*Imeretiopsis nakeralaensis* sp. nov. **Holotype**LT	2.00	1.09	1.02	0.85	0.64	0.53	29	1.84	1.20	1.28	1.96	3.13	2.04	8.55

##### Description.

***Shell***: rather small, 1.38–1.47 mm high, elongate-conical with four whorls, blunt and flat apex, inflated whorls and deep suture. Umbilicus narrow, almost closed. Shell surface glossy, milky white with irregular growth lines, randomly forming faint, rib-like structures. Aperture irregularly oval, slightly depressed from columellar side and slightly expanded. Lateral labral profile very weakly sinuated, columellar profile rather straight.

***Operculum***: not known.

***Animal body***: not known.

***Holotype measurements***: H-1.47 mm; W-0.74 mm; BH-0.85 mm; BW-0.68 mm; AH-0.50 mm; AW-0.47 mm; CA: 35°.

***Anatomy***: not known.

##### Etymology.

Named after the type locality, Iazoni Cave (იაზონის მღვიმე) (= Tskal-Tsiteli = Rioni Cave (= წყალწითელას = რიონის მღვიმე)) in Kutaisi.

##### Habitat.

Stygobiotic species. See habitat of *Imeretiopsis cameroni* sp. nov.

##### Distribution.

Only known from the type locality.

##### Conservation status.

The number of known locations (1) is no more than 5 and EOO is smaller than 20 km^2^. There is no reason to suppose that AOO, EOO, number of locations, number of subpopulations or the number or mature individuals are declining however due to its extremely small EOO we assessed as Vulnerable (VU) D2.

##### Remarks.

The assignment to the genus *Imeretiopsis* gen. nov. is only provisional due to sinuated aperture margins and resemblance to *I. gorgoleti*. sp. nov.; molecular data will be needed to determine its true taxonomic status. The type locality has indications of occasional pollution, and most of the stygobiotic Mollusca endemic to the cave (*M. borutzkii* (Shadin, 1932), *Euglesa subterranea* (Shadin, 1932) and *Imeretiopsis cameroni* sp. nov.) have shown declining populations. The new species is scarcer than all of the sympatric species.

#### Genus Caucasopsis

Taxon classificationAnimaliaLittorinimorphaHydrobiidae

Grego & Mumladze gen. nov.

65E19D73-6DF6-5BBC-8652-527192705777

http://zoobank.org/7FAE27A6-16B9-4946-A5E8-4CAD2DCF6C33

##### Type species.

*Caucasopsis letsurtsume* Grego & Mumladze, sp. nov.

##### Species assigned to the genus.

*Paladilhiopsis shadini* Starobogatov, 1962, *Paldilhiopsis subovata* Starobogatov, 1962; *Paladilhiopsis pulcherrima* Starobogatov, 1962; *Paladilhiopsis orientalis* Starobogatov, 1962 *Paladilhiopsis schakuranica* Starobogatov, 1962; *Paladilhopsis aculeus* Starobogatov, 1962; *Caucasopsis letsurtsume* Grego & Mumladze, sp. nov., *Caucasopsis olsavskyi* Grego & Mumladze, sp. nov., *Caucasopsis egrisi* Grego & Mumladze, sp. nov.

##### Diagnosis.

The new genus has a shell shape similar to members of the genus *Imeretiopsis* gen. nov. from more eastern localities of the Imereti region, which have, in contrast, a sinuated labral lateral profile. However, both genera can be clearly distinguished by their penes (Fig. 11A, B): the penis is long, with the filament (lacking in *Imeretiopsis*) and, below the filament, delicately marked outgrowth on the left side (in *Imeretiopsis* there are two broad outgrowths).

##### Etymology.

The name derived from the prefix *Caucas*- referring to the distribution range in the Caucasus Mountains and suffix –*opsis* reminiscent of the previously applied genus *Paladilhiopsis Pavlović*, 1913, adopted by Starobogatov (1962) for the similar shelled species from Abkhazia and from the Sochi region (Russia). Its gender is feminine.

##### Distribution.

The new genus *Caucasopsis* is known from the Samegrelo region, and likely from the Abkhazia and Sochi regions in the Russian Federation (Fig. 10).

#### Caucasopsis letsurtsume

Taxon classificationAnimaliaLittorinimorphaHydrobiidae

Grego & Mumladze sp. nov.

A0611C73-4178-55B1-B421-BABB742B6350

http://zoobank.org/06134CEA-7B68-4E06-939E-D47A2B991109

**Figure 12.**
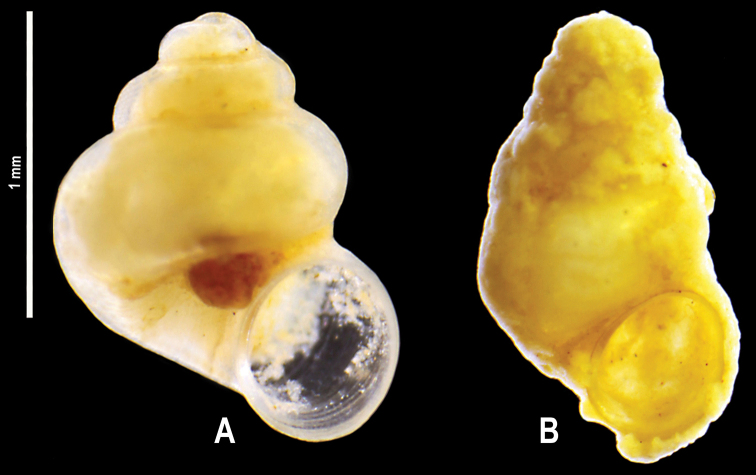
**A, B***Caucasopsis letsurtsume* sp. nov., Samegrelo, Chkhorotsku, Letsurtsume, Letsurtsume Cave, specimens used for molecular studies. Photograph A. Falniowski.

Plates 9 (2, 3, 10); 10(1, 2, 4); 11(1–4); Figs 11A, B , 12A, B

##### Type locality.

Georgia • Samegrelo, Chkhorotsku, Letsurtsume, Letsurtsume Cave (ლეწურწუმეს მღვიმე); 42°32'21"N, 42°06'48"E; 180 m a.s.l.; sandy sediment in cave stream.

**Plate 10.**
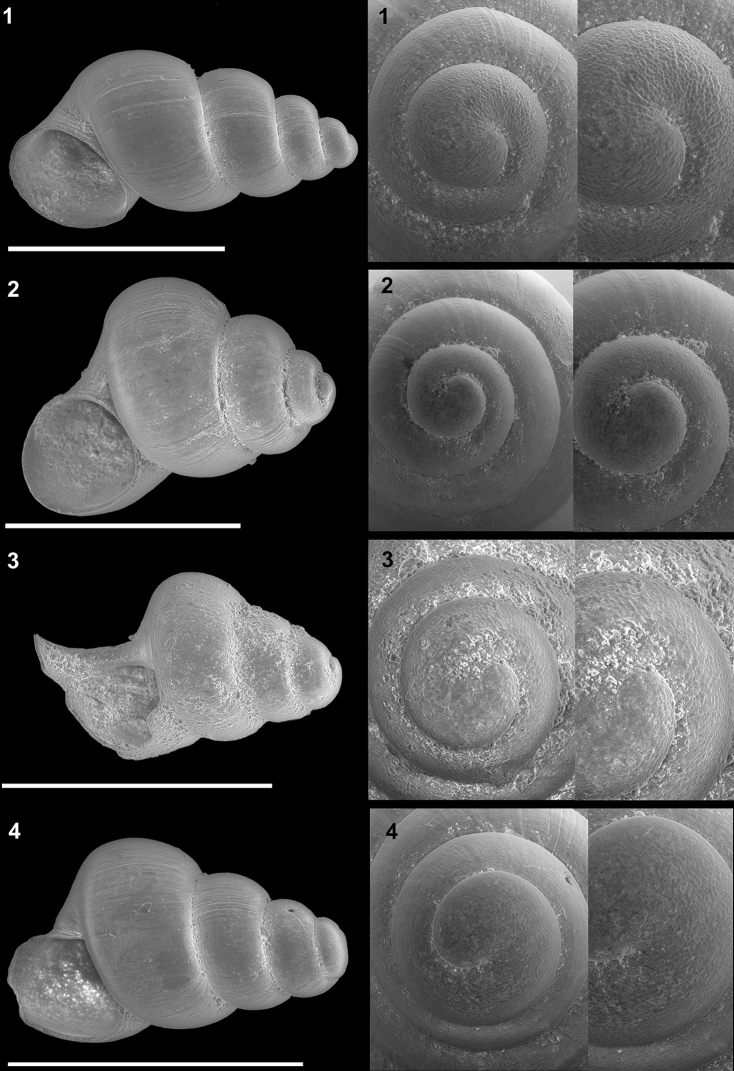
**1***Caucasopsis letsurtsume* sp. nov., Samegrelo, Chkhorotsku, Letsurtsume Cave, paratype SBMNH 633079 **2***C. letsurtsume* sp. nov., Samegrelo, Chkhorotsku, Letsurtsume, Letsurtsume Cave, morphotype SBMNH 633078 **3***C. olsavskyi* sp. nov., Samegrelo, Chkhorotsku, Nazodelavo Cave, paratype SBMNH 633081 **4***C. letsurtsume* sp. nov., Samegrelo, Chkhorotsku, Kachara Cave, SBMNH 633080. Scale bars: 1 mm. SEM SBMNH Vanessa Delnavaz.

**Plate 11.**
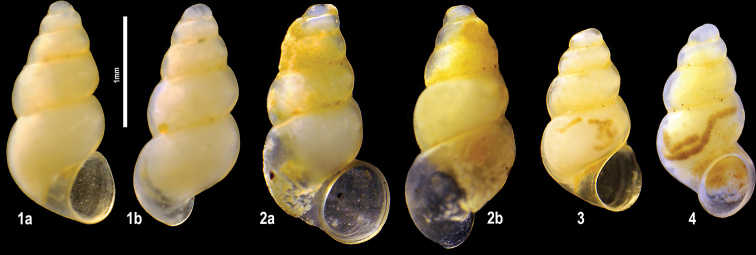
**1–4***Caucasopsis letsurtsume* sp. nov., Samegrelo, Chkhorotsku, Kachara Cave, specimens used for molecular and anatomical study. The numbers correspond to individuals, and the letters represent the different views of the same individual. Photograph A. Falniowski.

##### Material.

***Holotype***: Georgia • 1 adult, dry; type locality; 10 May 2018; J. Grego, L. Mumladze and M. Olšavský leg.; ISU FM-T010-H ***Paratypes***: same as for holotype; ISU FM-T010-P1/80 dry, SBMNH 633077/5 dry, NHMW 113368/2 dry, HNHM 104679/2 dry, NHMUK 0191335/2 dry, NHMB 563966/2 dry, SMF 358926/2 dry, ZMH 140631/2 dry, MNHN-IM-2016-7896, ZIN 1/510-2020/2 dry, coll. JG F1045/80 dry, coll. Glöer/1 dry. ***Other material***: Georgia • Samegrelo, Chkhorotsku, Kachara Cave (ქაჩარას მღვიმე); 42°31'47"N, 42°10'39"E; 205 m a.s.l.; sandy sediment at cave stream; 10 May 2018; J. Grego, L. Mumladze and M. Olšavský leg.; ISU FM/6 dry and 7 wet, SBMNH 633080, coll. JG F1051/6 dry and 8 wet. Georgia • Samegrelo, Chkhorotsku, Garakha, Savekuo Cavern; 42°30'23"N, 42°08'46"E; 240 m a.s.l.; mud sediments in the spring pond; 12 June 2019; H. Reip leg.; coll. F. Walther/58, coll. J. Grego/3 dry.

##### Diagnosis.

*Caucasopsis letsurtsume* sp. nov. differs from its closest relatives by its elongate-oval shell with inflated whorls and open umbilicus with aperture situated more right of the columellar axis (to viewer; shell in apertural pose, apex up). *Caucasopsis letsurtsume* sp. nov. has a more robust shell with proportionally larger body whorl, smaller umbilicus and with different protoconch surface. *Caucasopsis olsavskyi* sp. nov. can be differentiated by its different shell shape, closed umbilicus and proportionally smaller aperture situated adjacent to the columellar axis. The shell of *C. egrisi* sp. nov. is more slender with less inflated whorls and more closed umbilicus. Its shell morphology also resembles *Imeretiopsis nakeralaensis* sp. nov., which has a more elongate shell, more open umbilicus, less inflated whorls and a proportionally smaller rounded aperture situated more left of the columella (to viewer; shell in apertural pose, apex up).

##### Description.

***Shell***: elongate-oval, 1.64 mm high with blunt apex, inflated, 4½ whorls and deep suture. Shell surface smooth, glossy with very faint growth lines. Umbilicus narrow, slit-like. Aperture ovoid in shape, attached to the body whorl only shortly by an indistinct groove. Lateral and columellar profiles of the aperture straight. Lateral profile of the body whorl slightly expanding. Protoconch densely pitted.

***Operculum***: paucispiral yellowish, horny elongate ellipsoid with excentric nucleus.

***Animal body***: not known.

***Holotype measurements***: H-1.64 mm; W-0.94 mm; BH-0.89 mm; BW-0.72 mm; AH-0.60 mm; AW-0.47 mm; CA: 34°.

***Anatomy***: the penis (Fig. 11A, B) simple, straight, proximally and medially broad, distally with a moderately long, narrow filament; below the filament a delicately marked outgrowth on the left side.

##### Etymology.

Name derived from the name of Letsurtsume Cave, the type locality of the species.

##### Habitat.

Stygobiotic species. Empty shells of the new species were found in the sandy sediments of a cave stream penetrating through Miocene conglomerate deposits. Live individuals were found on a blackish microbial slime covered surface of rocks and gravel at the bottom of cave stream.

##### Distribution.

Only known from the type locality.

##### Conservation status.

The number of known locations (3) is no more than 5 and EOO is smaller than 20 km^2^. There is no reason to suppose that AOO, EOO, number of locations, number of subpopulations or the number or mature individuals are declining however due to its extremely small EOO we assessed as Vulnerable (VU) D2.

##### Remarks.

The shell shape of the species varies considerably in the only known locality. A second morphotype occurs in the type locality and differs significantly in shell morphology from the typical form. It is characterised by a more inflated-conical shell with 4½ whorls, by proportionally larger body whorl and open umbilicus (Morphotype B, Plates 9(10); 10(2), Fig. 12A). Shell morphology is similar to the genus *Motsametia* Vinarski, Palatov & Glöer, 2014. However, the DNA sequences (COI and H3) of both morphotypes are almost identical (see 1Z82 and 1Z80 on molecular trees in Figs 5–7); we consider them for the time being as one species with extraordinary morphological variability. The occurrence of the robust morphotype in much lower ratio, and the few available anatomical data do not suggest a sexual dimorphism. No parasites explaining the malformation found.

The population of *C. letsurtsume* sp. nov. from Kachara Cave differs from the type series by less inflated whorls and more closed umbilicus. The molecular distance within Clade B (Fig. 7) is 0.007 for COI, which indicates, that very closely situated hypogean habitats could host typical populations as a result of early allopatric evolution without any recent communication among the two populations.

#### Caucasopsis olsavskyi

Taxon classificationAnimaliaLittorinimorphaHydrobiidae

Mumladze & Grego sp. nov.

204FCF06-5FC8-51CB-BF6D-2424D500690E

http://zoobank.org/F220AA48-63FE-4C96-A2CB-05D7AFEEF821

Plates 9 (1); 10(3)

##### Type locality.

Georgia • Samegrelo, Chkhorotsku, Nazodelavo Cave (ნაზოდელავოს მღვიმე); 42°30'18"N, 42°13'15"E; 275 m a.s.l.; sandy sediment in cave stream.

##### Material.

***Holotype***: Georgia • 1 adult, dry; type locality; 11 May 2018; J. Grego, L. Mumladze and M. Olšavský leg.; ISU FM-T009-H. ***Paratypes***: Georgia • same as for holotype; ISU FM-T009-P1/ dry, NHMW 113369/1 dry, coll. JG F1053/2 dry, coll. Glöer/1 dry.

##### Diagnosis.

The new species differs from its closest relatives by its oval shell shape, proportionally smaller aperture more close-set to the columella and closed umbilicus. There is some similarity to the shell shape of *C. subovata* (Starobogatov, 1962) from Abkhazia, however, the broken subfossil type does not allow more detailed comparison, and the drawing of the author within the description was likely just a reconstruction of the broken holotype.

##### Description.

***Shell***: is 1.50–1.96 mm high, elongate ovate-conical with rounded whorls and blunt apex. Surface smooth, whitish, occasionally with inorganic incrustations. Aperture proportionally small, flat-ovoid shaped, situated below larger body whorl. Lateral profile of labral margin straight, columellar margin very weakly sinuated. Umbilicus closed.

***Operculum***: not known.

***Animal body***: not known.

***Holotype measurements***: H-1.87 mm; W-0.85 mm; BH-0.94 mm; BW-0.77 mm; AH-0.60 mm; AW-0.45 mm; CA: 26°.

***Anatomy***: not known.

##### Etymology.

Named for our friend Mário Olšavský, geologist and speleologist from Banská Bystrica, Slovakia, who actively participated in the field trip to Georgia.

##### Habitat.

Stygobiotic species. Empty shells were found at the sandy bottom of the cave stream inside a conglomerate cave. The empty shells were very scarce, as an undetermined *Tschernomorica* sp. was more abundant in the type locality.

##### Distribution.

Only known from the type locality.

##### Conservation status.

The number of known locations (1) is no more than 5 and EOO is smaller than 20 km^2^. There is no reason to suppose that AOO, EOO, number of locations, number of subpopulations or the number or mature individuals are declining however due to its extremely small EOO we assessed as Vulnerable (VU) D2.

#### Caucasopsis egrisi

Taxon classificationAnimaliaLittorinimorphaHydrobiidae

Grego & Mumladze sp. nov.

9BAD2D70-2C5E-5B47-A977-481EA43A770E

http://zoobank.org/746702F9-8F03-425B-89C4-BFD55834BFC6

Plate 9 (4–6)

##### Type locality.

Georgia • Samegrelo, Pirveli Balda (პირველი ბალდა), spring in village above road; 42°29'2"N, 42°23'53"E; 300 m a.s.l.

##### Material.

***Holotype***: Georgia • 1. Adult, dry; type locality; 09 May 2018; J. Grego, L. Mumladze and M. Olšavský leg.; ISU FM-T007-H. ***Paratypes***: Georgia • same as for holotype; ISU FM-T007-P1/1 dry, coll. JG F1031/2 dry. • same as for holotype; 13 October 2019; J. Grego leg.; ISU FM- T007-P2/2 dry, coll. JG F1436/22 dry SBMNH 639553, HNMB 563965/1 dry, NHMW 113367/1 dry.

#### Caucasopsis cf. egrisi

Taxon classificationAnimaliaLittorinimorphaHydrobiidae

EABB3069-FDEB-53A9-B563-889CED093209

##### Other material.

Georgia • Samegrelo, Pirveli Balda, Motena Cave; 42°28'36"N, 42°23'29"E; 480 m a.s.l.; 09 May 2018; J. Grego, L. Mumladze and M. Olšavský leg.; coll. ISU FM-HYD1/2 dry and JG/3 dry.

##### Diagnosis.

The new species shows some similarity to the geographically isolated *C. olsavskyi* sp. nov. from Nazodelavo Cave near Chkhorotsku, but it differs by its by its more oval, elongate shells shape with proportionally larger body whorl, by larger and differently positioned aperture situated more left of the columella (to viewer; shell in apertural pose, apex up) and by the more closed umbilicus. Measurement comparison of *Caucasopsis* species is given in Table 6.

**Table 6.** Measurement comparison of species from genus *Caucasopsis* gen. nov.

*Genus species*	H	W	BH	BW	AH	AW	CA	H/W	AH / AW	W / BW	H/BH	H/AH	W / AW	H/(W- BW)
	mm	mm	mm	mm	mm	mm	deg.							
*Caucasopsis letsurtsume* sp. nov. Kachara Cave	1.83	1.00	1.11	0.89	0.64	0.53	33	1.83	1.20	1.12	1.65	2.87	1.88	17.20
	1.81	0.92	0.96	0.77	0.62	0.50	30	1.96	1.23	1.20	1.88	2.94	1.85	11.75
	1.96	1.00	1.06	0.81	0.65	0.54	32	1.96	1.21	1.24	1.85	3.00	1.86	10.20
	1.50	0.81	0.96	0.71	0.58	0.42	32	1.86	1.36	1.14	1.56	2.60	1.91	15.60
*Caucasopsis letsurtsume* sp. nov. **Holotype**LT (form A)	1.64	0.94	0.89	0.72	0.60	0.47	34	1.75	1.27	1.29	1.83	2.75	2.00	7.70
*Caucasopsis letsurtsume* sp. nov. **Paratype**LT (form B)	1.72	1.23	1.19	1.00	0.72	0.58	2	1.40	1.24	1.23	1.45	2.39	2.12	7.48
*Caucasopsis olsavskyi* sp. nov. **Holotype**LT	1.87	0.85	0.94	0.77	0.60	0.45	26	2.20	1.33	1.11	2.00	3.14	1.90	22.00
*Caucasopsis egrisi* sp. nov. Motena Cave	1.66	0.87	0.89	0.68	0.57	0.47	30	1.90	1.23	1.28	1.86	2.89	1.86	8.67
*Caucasopsis egrisi* sp. nov. **Holotype**LT	2.00	0.91	1.19	0.81	0.64	0.51	34	2.19	1.25	1.13	1.68	3.13	1.79	18.80
*Caucasopsis egrisi* sp. nov. **Paratype**LT	1.74	0.77	0.98	0.72	0.57	0.43	30	2.28	1.35	1.06	1.78	3.04	1.80	41.00

##### Description.

***Shell***: narrow elongate-oval, 1.66–2.00 mm high with 4½ slightly tumid whorls, blunt protoconch, and weak suture. Shell surface whitish and smooth with faint axial growth lines, covered by milky white periostracum and by inorganic incrustations. Aperture proportionally smaller vs. the body whorl and more close-set to the columellar axis. The peristome attached to the body whorl by a weak sulcus over approximately a quarter of its outline. Lateral and columellar labral profiles smooth-straight with no traces of any sinuation. Umbilicus closed.

***Operculum***: not known.

***Animal body***: not known.

***Holotype measurements***: H-2.00 mm; W-0.91 mm; BH-1.19 mm; BW-0.81 mm; AH-0.64 mm; AW-0.51 mm; CA: 34°.

***Anatomy***: not known.

##### Etymology.

Named after Egrisi (ეგრისი), the historical name of the Colchis Kingdom established in the region from the 13^th^ to the 1^st^ century BC (disestablished in 164 BC).

##### Habitat.

Stygobiotic species. The secondary position where the empty shells of the new species were found is the spring head of small springs in village Pirveli Balda emerging from the stone debris at foot of the limestone plateau. The primary subterranean habitat is inaccessible and unknown.

##### Distribution.

Only known from the type locality; the similar shells can be found in a nearby Motena Cave.

##### Conservation status.

The number of known locations (2) is no more than 5 and EOO is smaller than 20 km^2^. There is no reason to suppose that AOO, EOO, number of locations, number of subpopulations or the number or mature individuals are declining however due to its extremely small EOO we assessed as Vulnerable (VU) D2.

##### Remarks.

The assignment of the new species to the genus *Caucasopsis* gen. nov. is only provisional, based on the sinuated lateral labral profile and on the locality, situated close to the distribution range of *Imeretiopsis* gen. nov. The molecular data will be essential to assign the species to the correct genus. The population in Motena Cave has slightly different shell morphology, and, despite their close localities, both represent different hydrological systems (perched water tables) separated by horizontal impermeable sandstone beds with more than 100 m difference in altitude. It is possible both populations could show separation at the species level; however, we prefer provisionally to treat them as one species until molecular data become available.

#### Genus Caucasogeyeria

Taxon classificationAnimaliaLittorinimorphaHydrobiidae

Grego & Mumladze gen. nov.

901A57E2-0B8D-5DD1-94CC-7BA4E80B9BE9

http://zoobank.org/AC1C48F4-F8E2-455B-AA9D-630CA818AAF5

##### Type species.

*Caucasogeyeria gloeri* Grego & Mumladze, sp. nov.

##### Species assigned to the genus.

“*Geyeria*” *valvataeformis* Starobogatov, 1962, “*Geyeria*” *horatieformis* Starobogatov, 1962, *C. gloeri* Grego & Mumladze, sp. nov., *Caucasogeyeria ignidona* Grego & Palatov, sp. nov., *C. colchis* Grego & Mumladze, sp. nov., *C. pseudocolchis* Grego & Mumladze, sp. nov., *C. chrysomallos* Grego & Mumladze, sp. nov.

##### Diagnosis.

The genus is well-separable from all other genera of the region by its conspicuously and deeply sinuated labral and columellar margins. The genus *Imeretiopsis* gen. nov., has much weaker and morphologically different labral sinuation, and the type species of the genus *Kartvelobia* gen. nov. has a very differently curled labral margin. The penis simple, long and narrow, different than in the genera mentioned above.

##### Etymology.

The prefix of the new species *Caucaso*- is derived from the distribution range of genus in the Caucasus Mountains, and the suffix –*geyeria* indicating the invalid genus “Geyeria”, previously applied for the genus by Starobogatov (1962). The genus name “Geyeria” was originally dedicated to the famous German malacologist David Geyer (6 November 1855–6 November 1932), who contributed greatly to the documentation of the German malacofauna. It was introduced by A. J. Wagner (1914) for the species “*Geyeria*” *plagiostoma* from the Bosna River springs near Sarajevo. However, the genus name proved permanently invalid due to junior homonymy, as it had been previously used by Buchecker in 1876 to name a moth in the family Castniidae Boisduval, 1828, Buckman 1899 for a cephalopod, Carapezzae and Schopen 1899 for a brachiopod, and Fucini 1901 for a cephalopod. Based on the homonymy, Tomlin in 1930 renamed the genus to *Plagigeyeria*. Later Starobogatov (1962) erroneously applied the invalid genus name to two stygobiotic species from the southwestern Caucasus (“*Geyeria*” *valvataeformis* and “*G.*” *horatieformis*). The gender is feminine.

**Plate 12.**
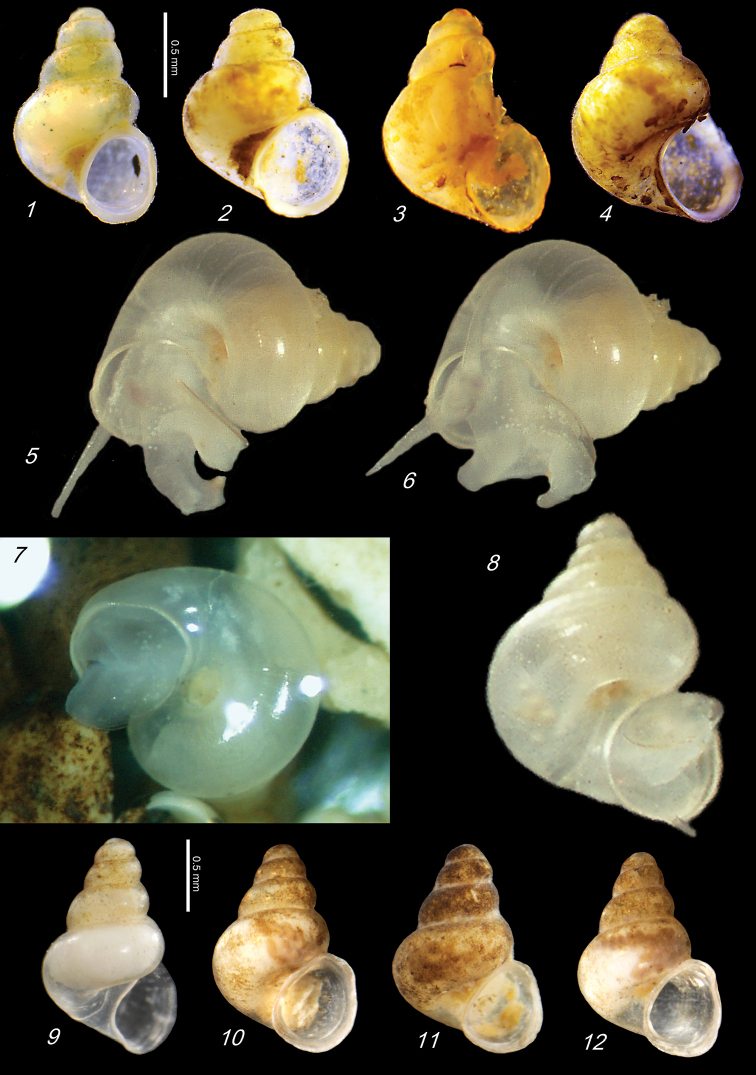
*Caucasogeyeria* specimens used for molecular and anatomical studies **1***C. chrysomallos* sp. nov. **2–4***C. ignidona* sp. nov. **5–8***C. colchis* sp. nov., live specimens Pirveli Balda, spring in the village **9–12***C. chrysomallos* sp. nov. The numbers correspond to individuals. Photograph A. Falniowski, J. Grego, A. Rysiewska.

##### Distribution.

**Figure 13.**
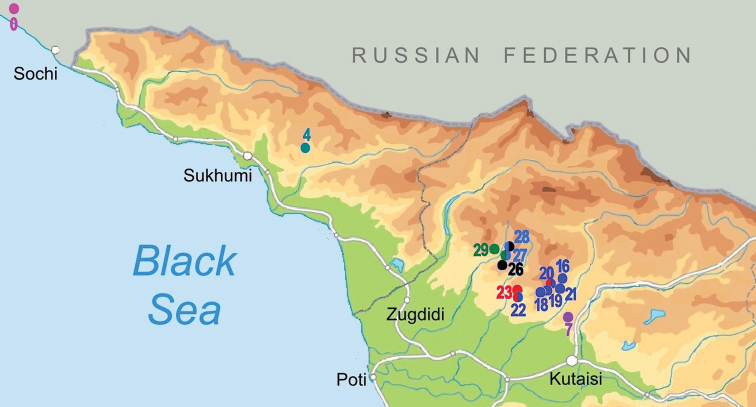
Distribution map of *Caucasogeyeria* gen. nov. **0***C. valvataeformis* (Starobogatov, 1962) (magenta dot) **4.***C. horatiaeformis* (Starobogatov, 1962) (turquoise dot) **16–22***C. gloeri* sp. nov. (blue dots) **27, 28**C. cf. gloeri (light blue dots) **7***C. ignidona* sp. nov. (purple dot) **20, 22, 23***C. colchis* sp. nov. (red dots) **26, 28***C. pseudocolchis* sp. nov. (black dots) **27, 29***C. chrysomallos* sp. nov. (green dots).

The genus is distributed on the Pakhe Plateau (situated S of Askhi Plateau) and in springs emerging around its slopes as well as at spring emerging from limestone massif north of Mukhuri settlement (Fig. 13).

#### Caucasogeyeria gloeri

Taxon classificationAnimaliaLittorinimorphaHydrobiidae

Grego & Mumladze sp. nov.

A902B762-A52C-56AD-92E2-262D1D0542A8

http://zoobank.org/2899DC4B-27E1-4ABF-AECD-74A7048A53C5

**Plate 13.**
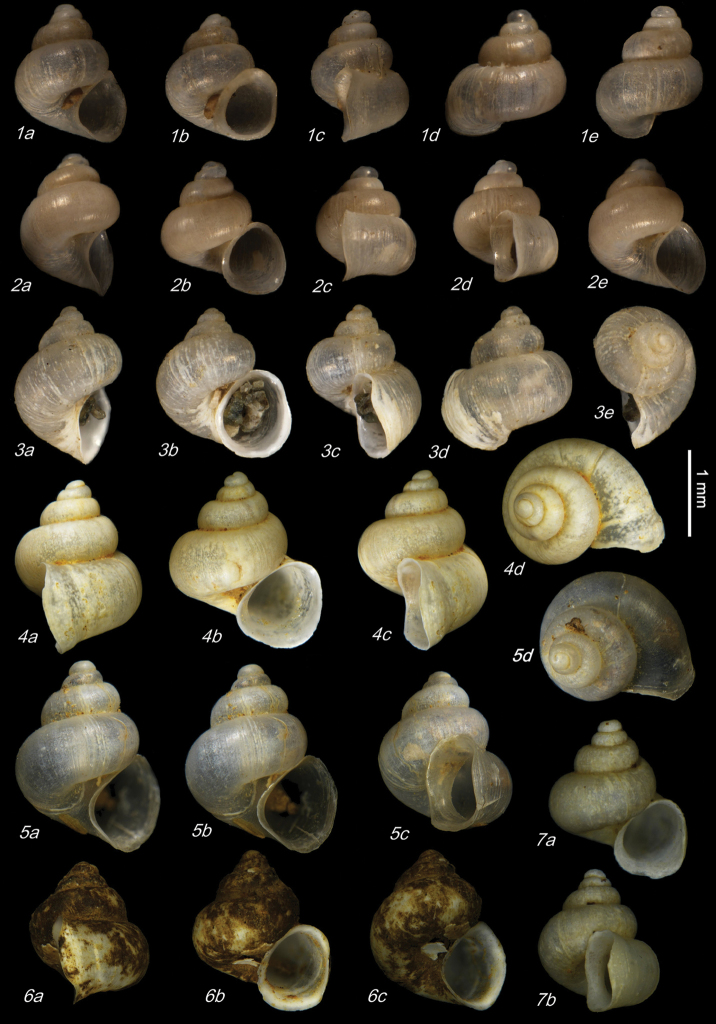
**1–7***Caucasogeyeria gloeri* sp. nov. **1, 2** Imereti, Satsiskvilo, Turchusmtha **1** holotype **2** paratype **4** Imereti, Nakhriduri, Spring at Turchu Gamosadivari Basin **5** Imereti, Turchusmtha, spring of Okatse and cave above Kinchkha waterfall **6** Imereti, Nakhriduri, Turchu Gamosadivari Cave Spring **7** Imereti, Nakhriduri, left tributary spring in Turchu Gamosadivari Basin **3**Caucasogeyeria cf. gloeri 2, Samegrelo, Shurubumu, spring on left bank of Khobistskali River. The numbers correspond to individuals, and the letters represent the different views of the same individual. Photograph J. Grego.

Plates 13 (1–7); 14(1–4); 16(9); 18(3)

##### Type locality.

Georgia • Imereti, Satsiskvilo, Turchusmtha (სოფელი საწისქვილო), small spring on the left side of path ascending the plateau; 42°29'25"N, 42°32'50"E; 980 m a.s.l.

**Plate 14.**
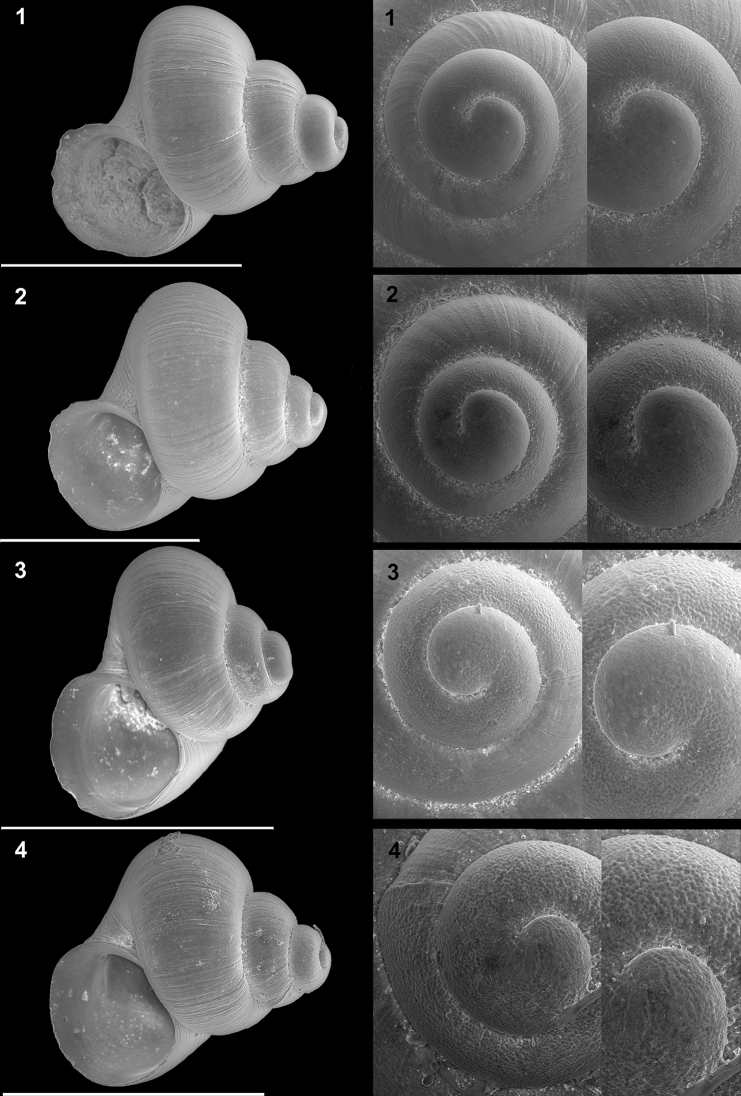
*Caucasogeyeria gloeri* sp. nov. **1** Imereti, Satsiskvilo, Turchusmtha, paratype SBMNH 633095 **2** Imereti, Nakhriduri, 1 paratype SBMNH 633102 **3** Imereti, Nakhriduri, spring at Turchu Gamosadivari Basin paratype SBMNH 633095 **4**Caucasogeyeria cf. gloeri, Samegrelo, Mukhuri, Shurubumu spring, paratype SBMNH 633085. Scale bars: 1 mm (SEM SBMNH Vanessa Delnavaz).

##### Material.

***Holotype***: Georgia • 1 adult, dry; type locality; 02 May 2018; J. Grego, L. Mumladze and M. Olšavský leg.; ISU FM-T003-H. ***Paratypes***: Georgia • same as for holotype; ISU FM-T003-P1/170 dry, SBMNH 633095/11 dry, NHMW 113363/2 dry, HNHM 104677/2 dry, NHMUK 20191333/2 dry, NHMB 563963 /2 dry, SMF 358924/2 dry, ZMH 140629/2 dry, MNHN-IM-2016-7894, ZIN 1/506-2020/2 dry, coll. JG F0990/170 dry, coll. Glöer/1 dry. ***Other material***: Georgia • Imereti, Nakhriduri 2 left tributary spring at Turchu Gamosadivari Basin; 42°28'39"N, 42°30'43"E; 870 m a.s.l.; 03 May 2018; J. Grego, L. Mumladze and M. Olšavský leg.; coll. JG/12 dry. • Imereti, Turchusmtha, Okatse Spring above Kinchkha waterfall; 42°29'49"N, 42°32'49"E; 1050 m a.s.l.; 02 May 2018; J. Grego, L. Mumladze and M. Olšavský leg.; coll. JG/19 dry. • Imereti, Nakhriduri, Turchu Gamosadivari Cave Spring; 42°29'13"N, 42°31'20"E; 954 m a.s.l.; 03 May 2018; E J. Grego, L. Mumladze and M. Olšavský leg.; coll. JG/4 dry. • Imereti, Nakhriduri left side tributary spring at Turchu Gamosadivari Basin above small ford; 42°28'39"N, 42°30'43"E; 875 m a.s.l.; 03 May 2018; J. Grego, L. Mumladze and M. Olšavský leg.; coll. JG/27 dry. • Imereti, Upskhero (უფსკერო) Spring Lake at Turchu Gamosadivari Basin; 42°27'47"N, 42°30'3"E; 960 m a.s.l.; 03 May 2018; J. Grego, L. Mumladze and M. Olšavský leg.; coll. JG/3 dry. • Imereti, Nakhriduri 1 at bottom of Turchu Gamosadivari Basin near farm house; 42°28'27"N, 42°30'13"E; 860 m a.s.l.; 03 May 2018; J. Grego, L. Mumladze and M. Olšavský leg.; coll. JG/2 dry. • Imereti, Nakhriduri 3 spring at Turchu Gamosadivari Basin left tributary spring; 42°28'40"N, 42°30'46"E; 875 m a.s.l.; 03 May 2018; J. Grego, L. Mumladze and M. Olšavský leg.; coll. JG/23 dry.• Imereti, Nakhiduri 2 left side spring above small ford; 42°28'39"N, 42°30'43"E; 870 m a.s.l.; 03 May 2018; J. Grego, L. Mumladze and M. Olšavský leg.; coll. JG/ dry. Georgia • Samegrelo, Pirveli Balda (პირველი ბალდა), spring in village above road; 42°29'2"N, 42°23'53"E; 300 m a.s.l. • Samegrelo, Pirveli Balda, Motena Cave; 42°28'36"N, 42°23'29"E; 480 m a.s.l.; 09 May 2018; J. Grego, L. Mumladze and M. Olšavský leg.; coll. ISU FM-HYD2/2 dry and JG/3 dry.

#### Caucasogeyeria cf. gloeri

Taxon classificationAnimaliaLittorinimorphaHydrobiidae

9261A6B3-F3C5-5112-8E97-5D055C59F644

##### Other material.

Georgia • Samegrelo, Shurubumu Spring (შურუბუმუს წყარო) on the left bank of Khobistskali River; 42°39'0"N, 42°12'21"E; 310 m a.s.l.; 10 May 2018; J. Grego, L. Mumladze and M. Olšavský leg.; ISU FM-HYD2/5 dry, SBMNH 633085/1, NHMW 113364/1 dry, coll. JG F0988/4 dry. • Samegrelo, Mapeli Cave (მაპელის მღვიმე), Mukhuri, water catchment (above cemetery); 42°38'22"N, 42°11'39"E; 325 m a.s.l.; 12 October 2019; J. Grego, L. Mumladze and M. Olšavský leg.; ISU FM-HYD3/4 dry, coll. JG F1411/5 dry.

##### Diagnosis.

The new species differs from the other representatives of the genus by the aperture with a characteristic positive labral and negative columellar sinuations and pyramidal-triangular shell shape. From *C. ignidona* sp. nov. it can be distinguished by the different form of the aperture and its larger, more robust shell shape. *Caucasogeyeria colchis* sp. nov. has a more deeply cut labral sinuation at its junction with the body whorl (posterior canal), more inward reflexed mid-labral section and more elevated conical spire. *Caucasogeyeria chrysomallos* sp. nov. has a similar lateral labral profile, but the shell is significantly smaller with a more narrow-elongate conical shape with a sharper apex. The two species from Abkhazia (*C. valvataeformis* and *C. horatiaeformis*) differ in shell shape and lack sinuated labral and columellar margins.

##### Description.

***Shell***: conically shaped with 3½ inflated whorls and blunt apex, height 1.40–2.08 mm. The body whorl proportionally large and expands slightly towards the aperture. The shell surface milky whitish with dense faint regular axial growth lines, frequently covered by rusty-brown inorganic incrustations. The expanding irregular shaped aperture with a characteristic pronounced sinuation at its labral margin best seen in lateral profile. The sinuation slightly curved inward the aperture. The columellar margin with an inward sinuation. Umbilicus widely open. Protoconch surface smooth with almost invisible smooth pitting.

***Operculum***: translucent glass-whitish, circular, paucispiral with excentric nucleus.

***Animal body***: not pigmented, white coloured, eyeless with proportionally long tentacles.

***Holotype measurements***: H-1.40 mm; W-1.29 mm; BH-1.06 mm; BW-1.00 mm; AH-0.80 mm; AW-0.70 mm; CA: 26°.

***Anatomy***: not known.

##### Etymology.

Named after the renowned German malacologist Peter Glöer from Hetlingen (Germany), who contributed much to the study of Eurasian freshwater Mollusca as well as the knowledge of Ponto-Caspian freshwater biodiversity.

##### Habitat.

Stygobiotic species. Most of the empty shells of the new species were found in the sandy sediments of karst springs of all types, from large spring lakes down to very small water outlets emerging from tiny fissures among limestone slabs. The great number of empty shells in some localities with no live individuals suggest its habitat is deeper in underground fissures and caves with very limited survival at epigean habitats. The few live shells were obtained from a spring emerging from stone debris, after removing the larger stones from the spring head and digging ca. 60–80 cm inside the spring head.

##### Distribution.

*Caucasogeyeria gloeri* sp. nov. is known from the eastern range of limestone Pakhe Plateau from Kinchkhaperdi to Satsiskvilo and in all springs of the Turchu Gamosadivari Basin in Imereti region. The isolated population from Shurubumu Spring and Mapeli Cave at Mukhuri (C. cf. gloeri), Samegrelo region (Plates 13(3), 14(4) and 16(9)) could represent a geographical subspecies or a distinct species.

##### Conservation status.

The number of known locations is 13 and EOO is ca. 70 km^2^. The AOO is represented by only several underground karst conduits with much smaller total area compared to EOO. Each karst conduit is supplied by surface water through swallow holes, where stochastic events, as human driven pollution or habitat destruction, could lead to rapid species decline or extinction. Therefore, it is assessed as Vulnerable (VU) D2.

##### Remarks.

The shell shape of the new species is quite variable over its range, but the typical features, such as the apertural sinuation seem to be more-or-less constant. A more intensive search in areas between the two main distribution points would be necessary to understand the phylogenetic relations of different populations. The population from Shurubumu Spring and Mapeli Cave is conchiologically very similar, however differs significantly by more coarsely pitted protoconch surface, molecular data are needed to confirm its specific or sub-specific status. Measurement comparison of different *C. gloeri* populations is given in Table 7.

**Table 7.** Measurement comparison of *Caucasogeyeria gloeri* sp. nov. from different localities.

*Genus species*	H	W	BH	BW	AH	AW	CA	H/W	AH / AW	W / BW	H/BH	H/AH	W / AW	H/(W- BW)
	mm	mm	mm	mm	mm	mm	deg.							
*Caucasogeyeria gloeri* sp. nov. **Holotype**LT	1.40	1.29	1.06	1.00	0.80	0.70	26	1.09	1.14	1.29	1.32	1.75	1.84	4.83
*Caucasogeyeria gloeri* sp. nov. **Paratype**LT	1.46	1.21	1.03	1.00	0.80	0.71	24	1.21	1.13	1.21	1.42	1.83	1.70	6.95
*Caucasogeyeria gloeri* sp. nov. Nakriduri 1 spring	1.97	1.71	1.37	1.31	0.97	0.94	45	1.15	1.03	1.31	1.44	2.03	1.82	4.93
*Caucasogeyeria gloeri* sp. nov. Okatse Spring	2.08	1.69	1.34	1.37	1.09	0.88	45	1.23	1.24	1.23	1.55	1.91	1.92	6.50
*Caucasogeyeria gloeri* sp. nov. Turchu Gamosadivari	1.67	1.54	1.31	1.20	0.97	0.80	45	1.08	1.21	1.28	1.27	1.72	1.93	4.91
*Caucasogeyeria gloeri* sp. nov. Nakriduri 4	1.81	1.54	1.31	1.20	0.97	0.80	46	1.18	1.21	1.28	1.38	1.87	1.93	5.32
*Caucasogeyeria gloeri* sp. nov. Shurubumu	1.69	1.48	1.37	1.11	1.00	0.86	45	1.14	1.16	1.33	1.23	1.69	1.72	4.57
*Caucasogeyeria gloeri* sp. nov. Mapeli Cave	1.81	1.53	1.30	1.21	0.93	0.84	22	1.18	1.11	1.27	1.39	1.95	1.83	5.57

#### Caucasogeyeria ignidona

Taxon classificationAnimaliaLittorinimorphaHydrobiidae

Grego & Palatov sp. nov.

16F422A8-1C67-5FFF-9B36-1C753D667574

http://zoobank.org/6DE56E85-4B3D-431D-AE7F-2CB087B421CD

**Plate 15.**
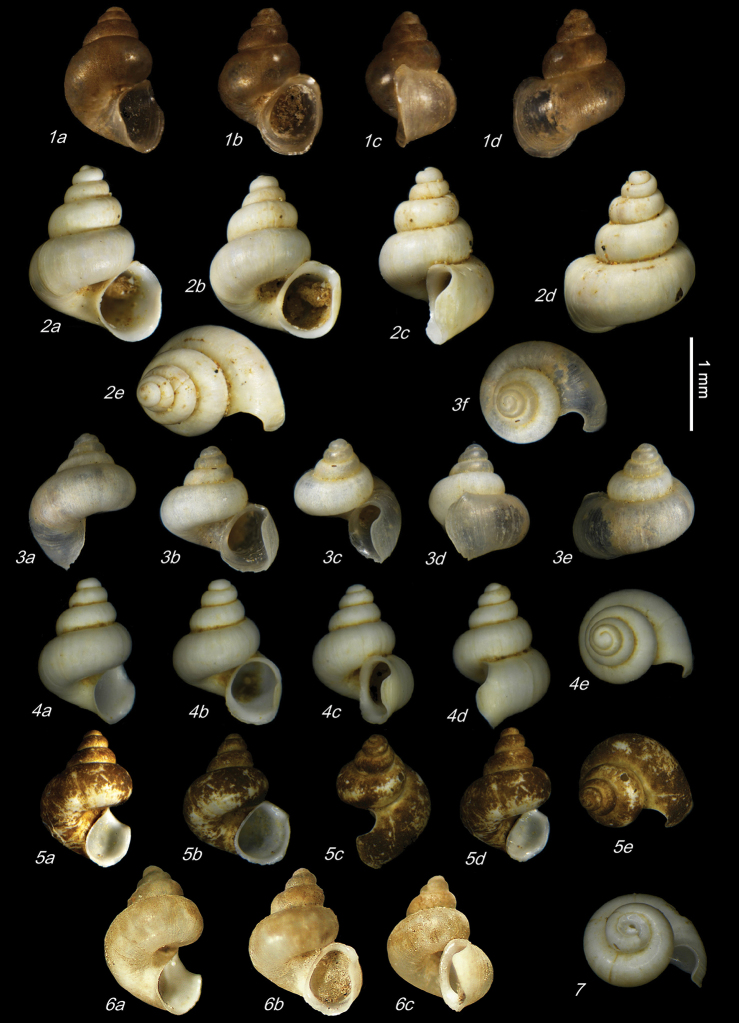
**1***Caucasogeyeria ignidona* sp. nov., Imereti, Kumistavi, Prometheus Cave, holotype **2***Caucasogeyeria colchis* sp. nov., Samegrelo, Pirveli Balda, Motena Cave, holotype **3***Caucasogeyeria colchis* sp. nov., Samegrelo, Pirveli Balda, spring in village, holotype **4***Caucasogeyeria colchis* sp. nov., Imereti, Nakhriduri 2 left side spring in Turchu Gamosadivari Basin above small ford, paratype **5–8***Caucasogeyeria pseudocolchis* sp. nov., Samegrelo, Mukhuri, Shisha Spring **5** holotype **6–8** paratypes. The numbers correspond to individuals, and the letters represent the different views of the same individual. Photograph J. Grego.

**Figure 14.**
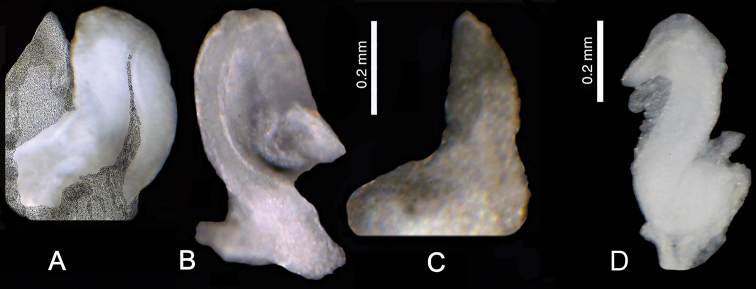
**A–C***Caucasogeyeria ignidona* sp. nov., Imereti, Kumistavi, Prometheus Cave **D***C. chrysomallos* sp. nov., Kanti, Mapeli Spring, morphology of the penis. Photograph A. Falniowski and A. Rysiewska.

Plate 15 (1); Fig. 14A–C

##### Type locality.

Georgia • Imereti, Kumistavi, Prometheus Cave (პრომეთეს მღვიმე); 42°22'33"N, 42°36'2"E; 175 m a.s.l.; bottom of cave stream.

##### Material.

***Holotype***: Georgia • adult, dry; type locality; 01 May 2018; J. Grego, L. Mumladze and M. Olšavský leg.; ISU FM-T005-H. ***Paratypes***: Georgia • same as for holotype; ISU FM-T005-P1/1 dry and 1 wet, coll. JG F0969/1 dry and 2 wet.

##### Diagnosis.

The new species can be distinguished from other members of the genus by the typical shell aperture. *Caucasogeyeria gloeri* sp. nov. has a larger, more robust shell with different aperture, *C. colchis* sp. nov. has more sinuated and more deeply cut labral margin at its columellar side, and *C. chrysomallos* sp. nov. has smaller, more conical and elongate shell with a greater number of whorls and proportionally smaller, differently shaped aperture.

##### Description.

***Shell***: conical with blunt protoconch and with 3½ inflated whorls separated by semi-deep suture. Height 1.4–1.7 mm. Shell surface milky white, glossy with occasional rusty brown incrustations. Aperture expanded, proportionally larger, rhomboidal with a weak negative sinuation at labral junction with the body whorl and a weak positive sinuation at columellar margin. Umbilicus slit-like.

***Operculum***: paucispiral, glass-like translucent.

***Animal body***: white, without eye spots.

***Holotype measurements***: H-1.60 mm; W-1.10 mm; BH-1.15 mm; BW-0.9 mm; AH-0.85 mm; AW-0.70 mm; CA: 28°.

***Anatomy***: the penis (Fig. 14A–C) bent, simple, narrow, gradually narrowing towards its distal end, vas deferens inside running straight.

##### Etymology.

Name derived from Latin word *ignidona* meaning of “donating fire”, referring to the gift of Prometheus to the mankind, indirectly indicating the name of type locality in the Prometheus Cave near Kutaisi.

##### Habitat.

Stygobiotic species. Live individuals of the new species were found in the cave stream on submerged stones and gravel, covered by a layer of dark brown-black layer of bacterial mats. Empty shells were found in sandy sediment of the cave stream.

##### Distribution.

Only known from the type locality.

##### Conservation status.

The number of known locations (1) is no more than 5 and EOO is smaller than 20 km^2^. There is no reason to suppose that AOO, EOO, number of locations, number of subpopulations or the number or mature individuals are declining however due to its extremely small EOO we assessed as Vulnerable (VU) D2.

##### Remarks.

The sympatric *Imeretiopsis prometheus* sp. nov. has more numerous populations throughout the cave stream. It is not clear whether both species share the micro-habitats within the same cave stream.

**Plate 16.**
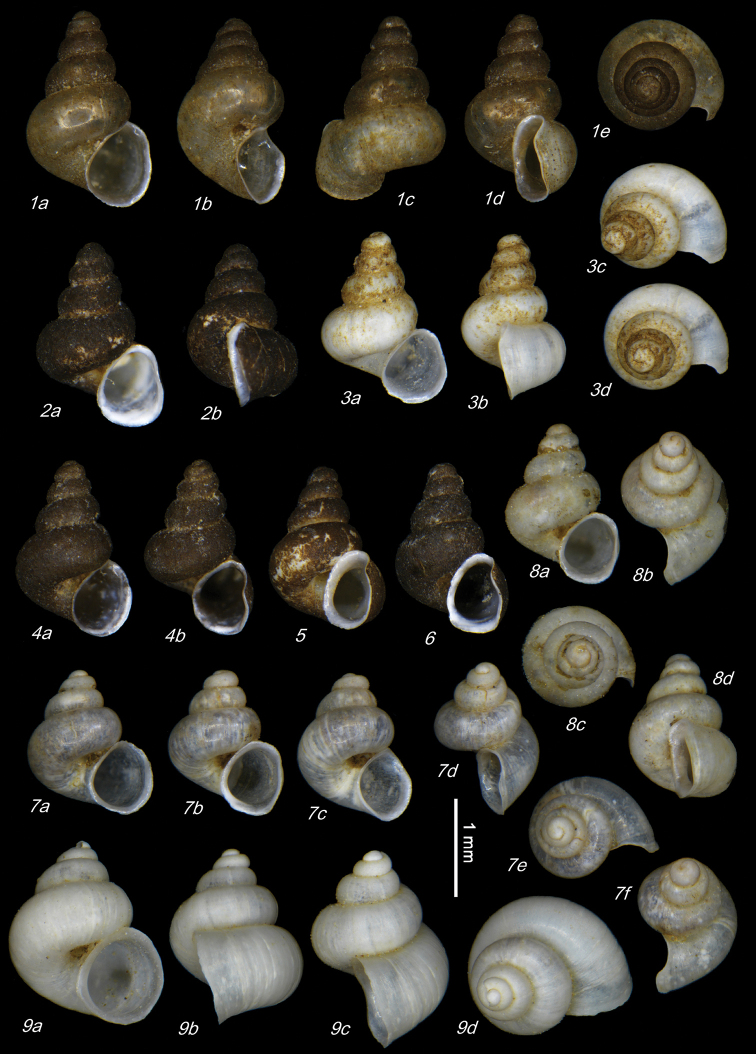
**1–6***Caucasogeyeria chrysomallos* sp. nov., Samegrelo, Mukhuri, Kanti, Mapeli Spring **1** holotype **2–6** paratypes **7, 8***Caucasogeyeria chrysomallos* sp. nov., Samegrelo, Mukhuri, Kanti, Mapeli Spring **9**Caucasogeyeria cf. gloeri, Samegrelo, Mukhuri, Mapeli Cave, paratype. The numbers correspond to individuals, and the letters represent the different views of the same individual. Photograph J. Grego.

#### Caucasogeyeria colchis

Taxon classificationAnimaliaLittorinimorphaHydrobiidae

Grego & Mumladze sp. nov.

1F98E046-6026-5385-A465-B8ED3A38FFBF

http://zoobank.org/740D6F25-3482-4F15-B21B-784B7C0319BE

Plates 15 (2–4); 17(1, 2)

##### Type locality.

Georgia • Samegrelo, Pirveli Balda, Motena Cave; 42°28'36"N, 42°23'29"E; 480 m a.s.l.; sediment in terminal lake.

**Plate 17.**
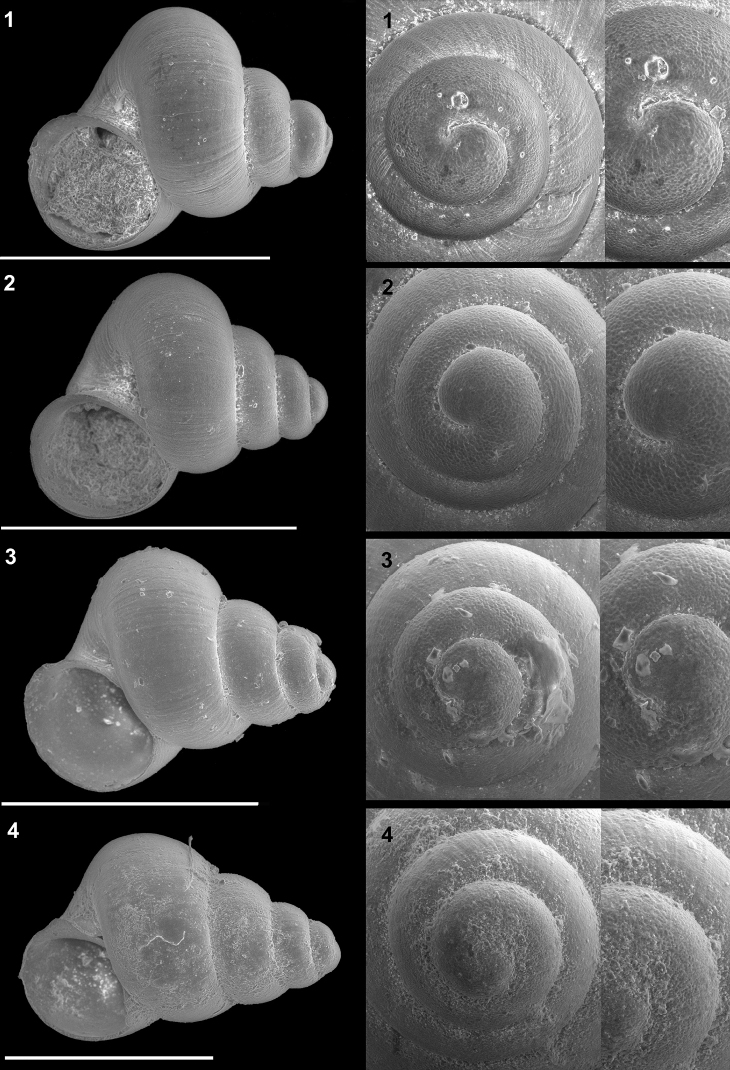
**1***Caucasogeyeria colchis* sp. nov., Samegrelo, Pirveli Balda, Motena Cave, paratype SBMNH 633072 **2***Caucasogeyeria colchis* sp. nov., Samegrelo, Pirveli Balda, spring in village, paratype SBMNH 633068 **3***Caucasogeyeria chrysomallos* sp. nov., Samegrelo, Mukhuri, Kanti, Mapeli Spring, paratype SBMNH 633099 **4***Caucasogeyeria pseudocolchis* sp. nov., Samegrelo, Mukhuri, Shisha Spring, paratype SBMNH 633082. Scale bars: 1 mm. SEM SBMNH Vanessa Delnavaz.

##### Material.

***Holotype***: Georgia • 1 adult dry; Type locality; 09 May 2018; J. Grego, L. Mumladze and M. Olšavský leg.; ISU FM-T002-H. ***Paratypes***: Georgia • same as for holotype; coll. JG T1036/1 dry; • Samegrelo, Pirveli Balda (პირველი ბალდა), spring in village above road; 42°29'2"N, 42°23'53"E; 295 m a.s.l.; 09 May 2018; J. Grego, L. Mumladze and M. Olšavský leg.; ISU FM-T002-P1/12 dry, SBMNH 633068/4 dry, NHMW 113362/1 dry, coll. JG F1034/12 dry. • same as preceding; same as preceding; 12 October 2019; J. Grego leg.; ISU FM-T002 P2/5 dry, HNHM 104676/1 dry, NHMB 563962 /1 dry, SMF 358923/1 dry, coll JG F1434/37 dry. ***Other material***: Georgia • Imereti, Nakhriduri 6, left tributary rivulet under travertine waterfall; 42°29'7"N, 42°31'22"E; 960 m a.s.l.; 03 May 2018; J. Grego, L. Mumladze and M. Olšavský leg.; coll. JG/4 dry.

##### Diagnosis.

The *C. colchis* sp. nov. differs from all the members of the genus by its more deeply cut sinuation at the junction of the labral margin with the body whorl. The sinulus-like deep grove and the characteristically inward bent labral fold clearly distinguish the species from its congeners. From *C. pseudocolchis* sp. nov. it can be distinguished mainly by shallower and narrower sinulus-like cut at the posterior canal, by the differently curved columellar peristome, different sinuation of the labral margin and by proportionally larger body whorl.

##### Description.

***Shell***: conical, elevated 1.35–1.80 mm high shell with 4½ inflated whorls and a deeply cut suture. Shell colour milky white with frequent reddish-brown inorganic encrustations. Umbilicus widely open. The expanded, rhomboidal aperture with a characteristic deep and broad sinus-like cut at the adapical labral junction with the body whorl. The protruded labral fold characteristically curved inward, continuing to a negative sinuation at the lower extremity of the aperture. Columellar margin just slightly positively sinuated. Protoconch surface regularly pitted.

***Operculum***: white, glassy translucent, circular and paucispiral with excentric nucleus.

***Animal body***: entirely white, without eyes and bears, very long tiny tentacles (Plate 12(5–8)).

***Holotype measurements***: H-1.80 mm; W-1.40 mm; BH-1.15 mm; BW-1.10 mm; AH-0.85 mm; AW-0.65 mm; CA: 37°.

***Anatomy***: not known.

##### Etymology.

Named after the ancient kingdom Colchis (კოლხეთი) established in the territory of the southwestern Caucasus and the Colchis lowland from the 13^th^ century BC to 164 BC.

##### Habitat.

Stygobiotic species. The scarce empty shells were found in the terminal sump lake of Motena Cave, and a few live individuals with some empty shells in the head of Pirveli Balda spring as it emerges from stone debris.

##### Distribution.

Except the type locality and the Motena Cave, the species is known from one locality in the Turchu Gamosadivari basin.

##### Conservation status.

The number of known locations (3) is no more than 5 and EOO is smaller than 20 km^2^. There is no reason to suppose that AOO, EOO, number of locations, number of subpopulations or the number or mature individuals are declining however due to its extremely small EOO we assessed as Vulnerable (VU) D2.

##### Remarks.

The species is sympatric with the *C. gloeri* sp. nov. in Nakhriduri 2 spring in the Turchu Gamosadivari Basin, Imereti and in Motena Cave and Piveli Balda spring in Samegrelo. Both species can be clearly separated by shell morphology without intermediates, indicating their separate specific position. Separation is confirmed by a p-distance = 0.034 in the H3 gene.

#### Caucasogeyeria pseudocolchis

Taxon classificationAnimaliaLittorinimorphaHydrobiidae

Grego & Mumladze sp. nov.

29398128-18C6-5456-B104-5F97E563E178

http://zoobank.org/36879DBA-9FA7-47B7-8AFC-9590900986DA

Plates 15 (5–8); 17(4); 18(2)

##### Type locality.

Georgia • Samegrelo, Mukhuri, Shisha Spring (სოფელი მუხური, შიშა წყარო); 42°37'47"N, 42°11'26"E; 255 m a.s.l.

##### Material.

***Holotype***: Georgia • 1 adult, dry; type locality; 10 May 2018; J. Grego, L. Mumladze and M. Olšavský leg.; ISU FM-T006-H. ***Paratypes***: Georgia • same as for holotype; coll. JG F1057/2 dry. • same as preceding; 10 October 2019; J. Grego, L. Mumladze and G. Bananashvili leg.; ISU FM-T006-P1/5 dry, SBMNH 635902/1 dry, coll. JG F1420/9 dry. • Samegrelo, Mukhuri, Shurubumu 1 Spring on left bank of Khobistskali River; 42°39'0"N, 42°12'21"E; 310 m a.s.l.; sediment at outlet; 10 May 2018; J. Grego, L. Mumladze and M. Olšavský leg.; coll. JG F1057/2 dry.

##### Diagnosis.

*Caucasogeyeria pseudocolchis* sp. nov. differs from all the members of the genus by its more deeply cut and broader sinuation at the posterior canal, at the junction of the labral margin with the body whorl. The larger sinulus-like deep grove and the characteristically unbent labral fold with a different aperture shape clearly distinguish the species from the closely related *C. colchis* sp. nov.

##### Description.

***Shell***: pyramidal with four inflated whorls, deeply cut suture and proportionally larger body whorl. Height 1.32–1.55 mm. The milky white shell with occasionally reddish brown inorganic encrustation. Umbilicus widely open. The expanded, rhomboidal aperture framed by a very deep and very broad cut at the posterior canal. The protruded labral fold straight, not curved inward. Labrum continues smoothly toward the lower extremity. Columellar margin is more or less straight. Protoconch surface with large regular deep pits.

***Operculum***: not known.

***Animal body***: not known.

***Holotype measurements***: H-1.45 mm; W-1.15 mm; BH-1.25 mm; BW-0.95 mm; AH-0.70 mm; AW-0.60 mm; CA: 28°.

***Anatomy***: not known.

##### Etymology.

Named after the very similar shell shape to the *C. colchis* sp. nov. known from the Pakhe Plateau near Pirveli Balda and Nakhriduri.

##### Habitat.

Stygobiotic species. Very worn and fragmented empty shells with only a few intact specimens were found in Shisha Spring, and a single live individual was found in a spring Shurubumu near Mukhuri. The condition of the material indicates a deep stygobiotic habitat far from the springhead with its accumulated recent thanatocoenoses.

##### Distribution.

Known only from the type locality at Shurubumu Spring and from Shisha Spring in the vicinity of Mukhuri.

##### Remarks.

The new species is sympatric with the C. cf. gloeri at Shurubumu Spring.

##### Conservation status.

The number of known locations (2) is no more than 5 and EOO is smaller than 20 km^2^. There is no reason to suppose that AOO, EOO, number of locations, number of subpopulations or the number or mature individuals are declining however due to its extremely small EOO we assessed as Vulnerable (VU) D2.

#### Caucasogeyeria chrysomallos

Taxon classificationAnimaliaLittorinimorphaHydrobiidae

Grego & Mumladze sp. nov.

6870E740-5A56-5100-9774-107EC7CA6B44

http://zoobank.org/5837CAA1-020A-46D1-9168-E1881C232D9F

Plates 16 (1–8); 17(3); 18(1); Fig. 14D

##### Type locality.

Georgia • Samegrelo, Kanti Village near Mukhuri, Mapeli Spring (სოფელი კანტი, მაპელის წყარო); 42°38'23"N, 42°10'08"E; 290 m a.s.l.

**Plate 18.**
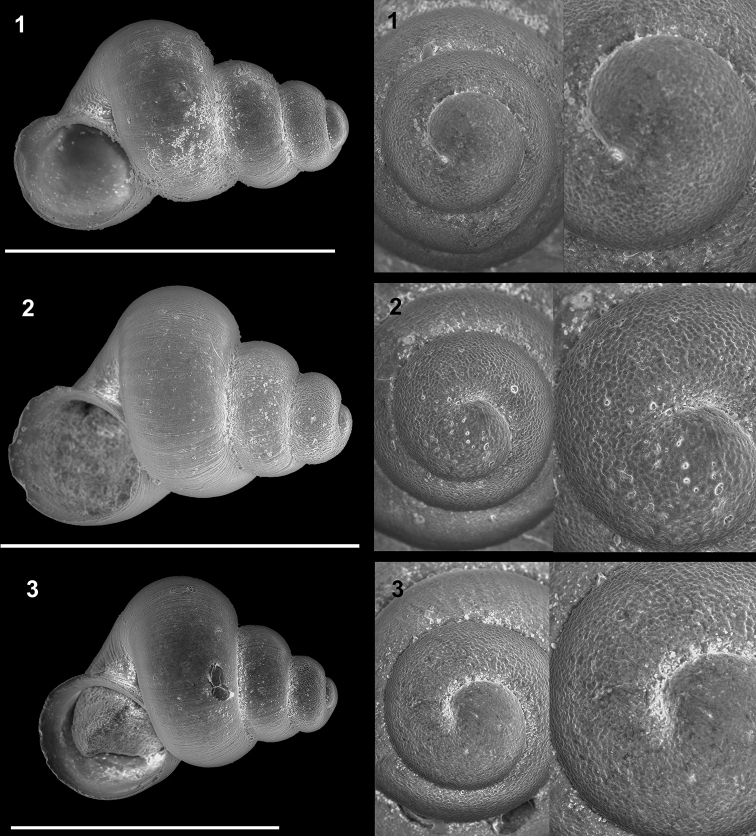
**1***Caucasogeyeria chrysomallos* sp. nov., Samegrelo, Mukhuri, Kanti, Mapeli Spring, paratype SBMNH 635903 **2***Caucasogeyeria pseudocolchis* sp. nov., Samegrelo, Mukhuri, Shisha Spring, paratype SBMNH 635902 **3**Caucasogeyeria cf. gloeri, Samegrelo, Mukhuri, Mapeli Cave, paratype SBMNH 635901. Scale bars: 1 mm. SEM SBMNH Vanessa Delnavaz.

##### Material.

***Holotype***: Georgia • 1 adult, dry; type locality; 12 October 2019; J. Grego, L. Mumladze and G. Bananashvili leg.; ISU FM-T001-H. ***Paratypes***: Georgia • same as for holotype; ISU FM-T001-P1/12 dry and 24 wet, SBMNH 633099/2 dry, NHMW 113365/1 dry, HNHM 104678/1 dry, NHMUK 20191334/2 dry, NHMB 563964/1 dry, SMF 358925/1 dry, NHMW 113365, ZMH 140630/1 dry, MNHN-IM-2016-7895, ZIN 1/507-2020/1 dry, coll. JG F1414/15 dry and 24 wet, coll. Glöer/1 dry. • same as for holotype; 10 May 2018; J. Grego, L. Mumladze and M. Olšavský leg.; coll. JG F1041/2 dry. ***Other material***: Georgia • Samegrelo, Mapeli Cave, Mukhuri, water catchment (above cemetery); 42°38'22"N, 42°11'39"E; 325 m a.s.l.; 10 May 2018; J. Grego, L. Mumladze and M. Olšavský leg.; coll. JG/3 dry.

##### Diagnosis.

The new species differs from all congeners by its smaller more elongate-conical shell with more numerous whorls combined with a smaller umbilicus. The aperture sinuation shows similarity with *C. gloeri* sp. nov., however, the latter has a larger, more robust and less elevated shell shape with the columellar sinuation. *Caucasogeyeria colchis* sp. nov. differs from new species by its larger size and more deeply sinuated labral margin. Measurement comparison of *Caucasogeyeria* species is given in Table 8.

**Table 8.** Measurement comparison of species from genus *Caucasogeyeria* gen. nov.

*Genus species*	H	W	BH	BW	AH	AW	CA	H/W	AH / AW	W / BW	H/BH	H/AH	W / AW	H/(W- BW)
	mm	mm	mm	mm	mm	mm	deg.							
*Caucasogeyeria ignidona* sp. nov. **Holotype**LT	1.60	1.10	1.15	0.90	0.85	0.70	28	1.45	1.21	1.22	1.39	1.88	1.57	8.00
*Caucasogeyeria colchis* sp. nov. **Holotype**LT	1.80	1.40	1.15	1.10	0.85	0.65	37	1.29	1.31	1.27	1.57	2.12	2.15	6.00
*Caucasogeyeria colchis* sp. nov. **Paratype** Priveli Balda	1.35	1.30	0.95	0.90	0.75	0.55	31	1.04	1.36	1.44	1.42	1.80	2.36	3.38
*Caucasogeyeria colchis* sp. nov. **Paratype** Nakriduri 2 spring	1.60	1.20	1.05	0.90	0.75	0.60	30	1.33	1.25	1.33	1.52	2.13	2.00	5.33
*Caucasogeyeria pseudocolchis* sp. nov. **Holotype** Shisha Spring	1.45	1.15	1.25	0.95	0.70	0.60	28	1.26	1.17	1.21	1.16	2.07	1.92	7.25
*Caucasogeyeria pseudocolchis* sp. nov. **Paratype** Shisha Spring	1.55	1.20	1.10	0.95	0.76	0.57	24	1.29	1.33	1.26	1.41	2.04	2.11	6.20
*Caucasogeyeria chrysomallos* sp. nov. **Holotype**LT	1.93	1.21	1.21	1.07	0.84	0.65	30	1.60	1.29	1.13	1.60	2.31	1.86	13.83
*Caucasogeyeria chrysomallos* sp. nov. **Paratype**LT	1.86	1.21	1.16	0.98	0.79	0.65	28	1.54	1.21	1.24	1.60	2.35	1.86	8.00
	1.77	1.23	0.98	0.95	0.74	0.63	33	1.43	1.19	1.29	1.81	2.38	1.96	6.33
	1.77	1.16	1.07	0.93	0.74	0.60	29	1.52	1.23	1.25	1.65	2.38	1.92	7.60
	1.67	1.16	1.05	0.93	0.72	0.60	33	1.44	1.19	1.25	1.60	2.32	1.92	7.20
	1.77	1.14	0.98	0.88	0.79	0.60	28	1.55	1.31	1.29	1.81	2.24	1.88	6.91
	1.40	1.23	0.98	0.98	0.70	0.65	30	1.13	1.07	1.26	1.43	2.00	1.89	5.45
	1.58	1.14	1.02	0.93	0.74	0.56	28	1.39	1.33	1.23	1.55	2.13	2.04	7.56

##### Description.

***Shell***: elongate conical with five tumid whorls, a semi-deep suture, a blunt apex and a narrow umbilicus. Height 1.40–1.93 mm. Shell surface smooth, covered by a milky white periostracum, frequently overlaid by thick dark brown-black inorganic precipitate. The expanded aperture irregularly pear shaped. Labral margin with a weak but broad negative sinuation near the body whorl junction, followed by a characteristic inward curved but shallow labral fold. Columellar margin is straight, not sinuated. Protoconch surface regularly pitted, pitting fading out at the nucleus.

***Operculum***: light yellow, paucispiral with central nucleus.

***Animal body***: without eye spots, not pigmented, whitish translucent.

***Holotype measurements***: H-1.93 mm; W-1.21 mm; BH-1.21 mm; BW-1.07 mm; AH-0.84 mm; AW-0.65 mm; CA: 30°.

***Anatomy***: penis (Fig. 14D) straight, simple, without any outgrowth.

##### Etymology.

Name derived from the Greek name *Chrysomallos*, meaning Golden Fleece (symbol of authority and monarchy), which, according to Greek mythology, was held in Colchis. Jason and his crew of Argonauts were sent out on a quest for the Golden Fleece by order of King Pelias.

##### Habitat.

Stygobiotic species. Live individuals as well as empty shells were washed out from its subterranean habitat through a small spring in Mapeli emerging near the road in village Kanti. The dense brown-black deposits on most of individuals indicates a subterranean habitat with chemolithotrophic bacteria. The second known population was found in the sediments of a subterranean cave stream inside Mapeli Cave, ca. 30 m from its entrance

##### Distribution.

Only known from the type locality and from Mapeli Cave.

##### Conservation status.

The number of known locations (2) is no more than 5 and EOO is smaller than 20 km^2^. There is no reason to suppose that AOO, EOO, number of locations, number of subpopulations or the number or mature individuals are declining however due to its extremely small EOO we assessed as Vulnerable (VU) D2.

##### Remarks.

The population in Mapeli Cave is typical but has a lower spire and fewer whorls. Its taxonomic position will be clarified after the collection of live individuals.

#### Genus Pontohoratia

Taxon classificationAnimaliaLittorinimorphaHydrobiidae

Vinarski, Palatov & Glöer, 2014

FDA37D44-903B-56A5-BAE4-25C982C06ADE

##### Type species.

*Horatia birsteini* Starobogatov, 1962

##### Species assigned to the genus.

*Pontohoratia birsteini* (Starobogatov, 1962), *P. smyri* Vinarski, Palatov & Glöer, 2014, *P. vinarskii* Grego & Mumladze, sp. nov., *P. pichkhaiai* Grego & Mumladze, sp. nov., *P. mapeli* Grego & Mumladze, sp. nov.

##### Distribution.

**Figure 15.**
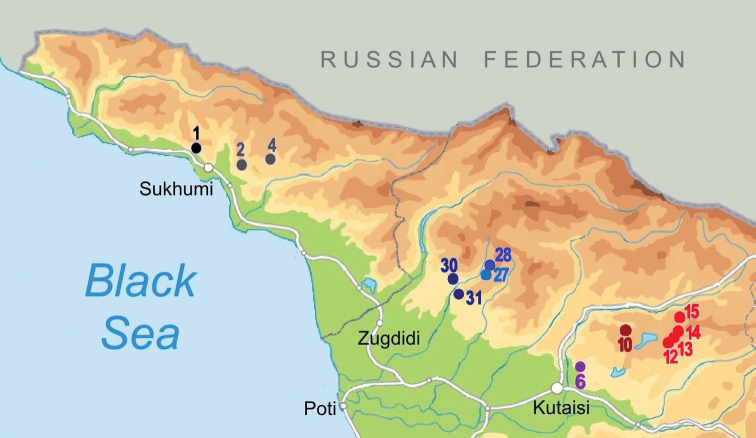
Distribution map of genera *Pontohoratia* Vinarski, Palatov & Glöer, 2014; *Motsametia* Vinarski, Palatov & Glöer, 2014 and *Hausdorfenia* gen. nov. **1***P. smyri* Vinarski, Palatov & Glöer, 2014 (black dot) **2–4***P. birsteini* (Starobogatov, 1962) (grey dots) **6***M. borutzkii* (Shadin, 1932) (purple dot) **10***H. shareula* sp. nov. (dark red dot) **12–15***H. hauffeniaformis* sp. nov. (red dots) **27***P. pichkhaiai* sp. nov. (light blue dot) **28***P. mapeli* sp. nov. (medium blue dot) **30–31***P. vinarskii* sp. nov. (dark blue dot).

The genus is known from Samegrelo region around Mukhuri and from Abkhazia in the vicinity of Sukhumi (Fig. 15).

#### Pontohoratia vinarskii

Taxon classificationAnimaliaLittorinimorphaHydrobiidae

Grego & Mumladze sp. nov.

5F0459E1-4BC6-5E6A-ACA4-D9890AC9584A

http://zoobank.org/785C454A-73B0-4C37-AAB4-A9989B268236

**Plate 19.**
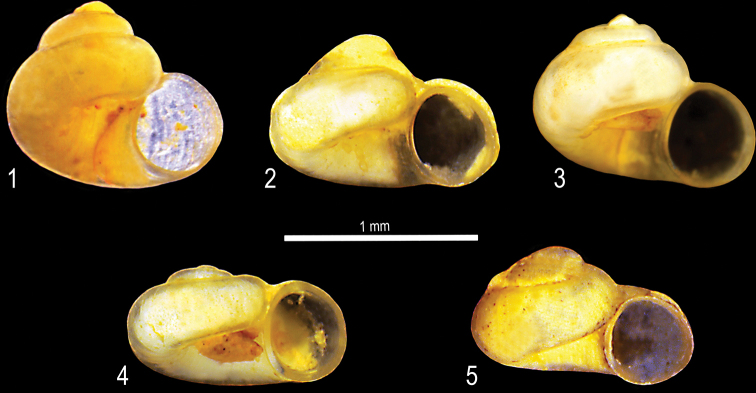
**1–5***Pontohoratia vinarskii* sp. nov.: Samegrelo, Chkhorotsku, Letsurtsume Cave, specimens used for molecular and anatomical study. The numbers correspond to individuals. Photograph A. Falniowski.

**Figure 16.**
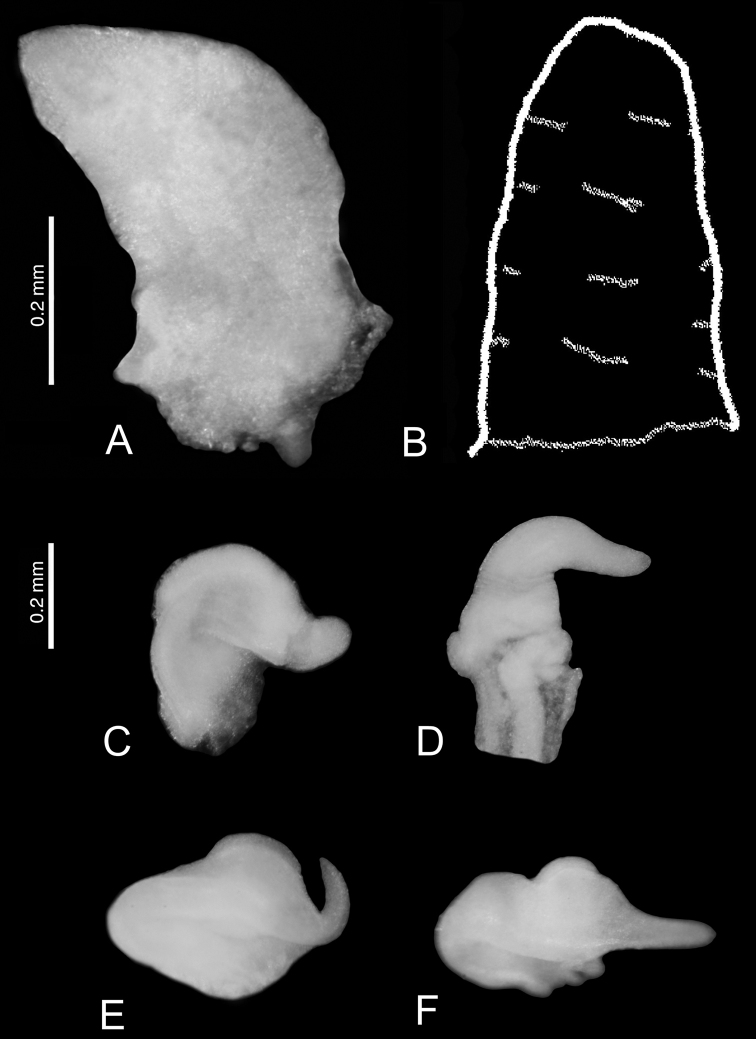
**A, B***Pontohoratia vinarskii* sp. nov.: Samegrelo, Chkhorotsku, Nazodelavo Cave, morphology of penis **C, D***P. pichkhaiai* sp. nov.: Samegrelo, Chkhorotsku, Shisha Spring, morphology of penis **E, F***P. mapeli*. sp.: Samegrelo, Kanti, Mapeli Spring, morphology of penis. Photograph A. Falniowski and A. Rysiewska; drawing A. Falniowski.

Plates 19 (1–5); 20(1, 2, 8, 9); Fig. 16A, B

##### Type locality.

Georgia • Samegrelo, Chkhorotsku, Letsurtsume, Letsurtsume Cave (ლეწურწუმეს მღვიმე); 42°32'21"N, 42°06'48"E; 180 m a.s.l; sandy sediment in the cave stream bottom.

##### Material.

***Holotype***: Georgia • 1 adult, dry; type locality; 02 May 2018; J. Grego, L. Mumladze and M. Olšavský leg.; ISU FM-T023-H. ***Paratypes***: Georgia • same as for holotype; ISU FM-T023-P1/350 dry and 18 wet, P2/10 dry, SBMNH 633077/5 dry, NHMW 113377/3 dry, HNHM 104686/3 dry, NHMUK 20191341/3 dry, NHMB 563974/3 dry, SMF 358934/3 dry, ZMH 140638/3 dry, MNHN-IM-2016-7902, ZIN 1/505-2020/3 dry, coll. JG F1046/350 dry and 18 wet, JG F1047/10 dry, coll. Glöer/1 dry. ***Other material***: Georgia • Samegrelo, Chkhorotsku, Garakha, Savekuo Cavern, mud sediments in the spring pond; 42°30'23"N, 42°08'46"E; 240 m a.s.l.; 12 June 2019; H. Reip leg.; coll. F. Walther/243 dry, coll. JG/20 dry.

**Plate 20.**
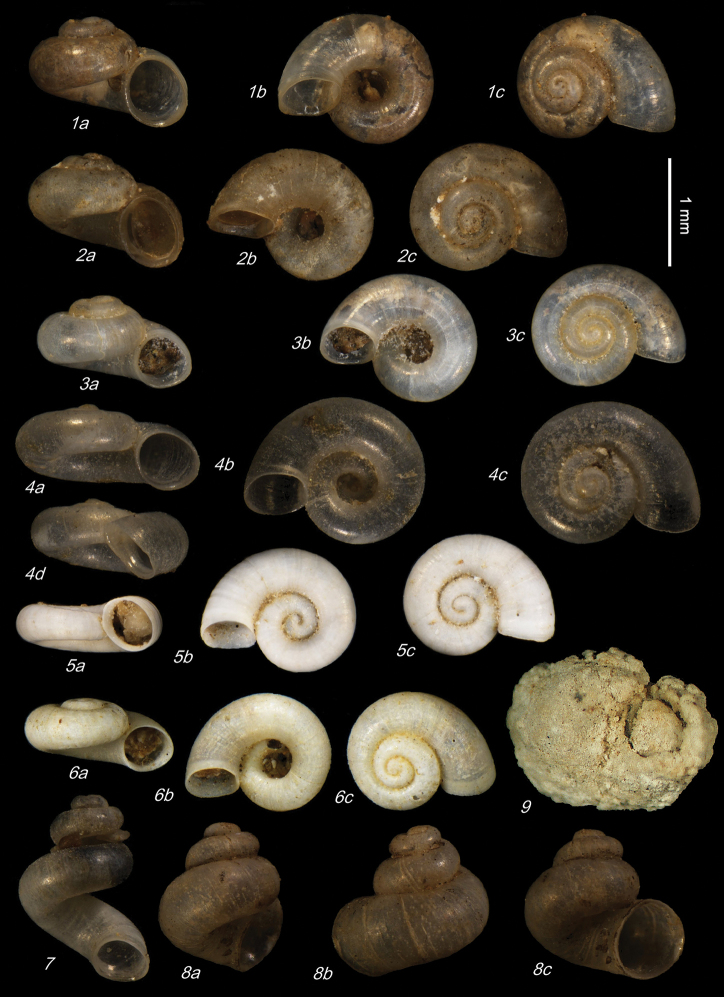
**1, 2***Pontohoratia vinarskii* sp. nov., Samegrelo, Chkhorotsku, Letsurtsume, Letsurtsume Cave: **1** holotype **2** paratype **3***P. pichkhaiai* sp. nov., Samegrelo, Mukhuri, Shisha Spring, holotype **4***Hausdorfenia pseudohauffenia* sp. nov., Racha, Zemo Krikhi, Krikhula Spring, holotype **5***H. shareula* sp. nov., Racha, Nikortsmintha, Tsivtskala 2 Spring near power station in the Shareula valley, holotype **6***P. mapeli* sp. nov., Samegrelo, Mukhuri, Kanti, Mapeli Spring, holotype **7***H. pseudohauffenia* sp. nov., Racha, Zemo Krikhi, Krikhula Spring; aberrant specimen **8, 9***P. vinarskii* sp. nov., Samegrelo, Chkhorotsku, Letsurtsume, Letsurtsume Cave: **8** morphotype **9** live specimen with marked carbonate incrustations. The numbers correspond to individuals, and the letters represent the different views of the same individual. Photograph J. Grego.

##### Diagnosis.

The new species differs from *P. smyri* Vinarski, Palatov & Glöer, 2014 by its more elevated spire and by a proportionally smaller and more ovate aperture. The geographically close *C. pichkhaiai* sp. nov. and *C. mapeli* sp. nov. have similar shells, but both are flatter and have much smaller rounded apertures.

##### Description.

***Shell***: flat, discoid with elevated spire and flat apex. Diameter 1.31–1.58 mm. Umbilicus widely opened. The 2¾ whorls are separated by a deeply cut sulcus. Shell transparent whitish colour with smooth surface and almost invisible growth lines. Oval aperture with axis declined towards columella. Peristome smooth without any folds. Lateral profile of the labrum is slightly angled towards the body whorl at its upper side, where attached by a narrow furrow. Protoconch surface regularly weakly pitted on the nuclear portion and abapically smooth.

***Operculum***: orange coloured circular, translucent, with central nucleus, thickened at its centre, but without peg on its inner side.

***Animal body***: whitish, not pigmented, eyeless.

***Holotype measurements***: H-1.08 mm; W-1.47 mm; BH-0.87 mm; BW-1.00 mm; AH-0.63 mm; AW-0.55 mm; CA: –20°.

***Anatomy***: the penis (Fig. 16A, B) simple, broad and blunt, without any outgrowth.

##### Etymology.

Named after renowned Russian malacologist Maxim V. Vinarski, Saint-Petersburg State University, Russia, who contributed significantly to Eurasian freshwater Mollusca studies as well as to the study of southwestern Caucasus freshwater Mollusca.

##### Habitat.

Stygobiotic species. See habitat of *Caucasopsis letsurtsume* sp. nov.

##### Distribution.

Only known from the type locality.

##### Conservation status.

The number of known locations (2) is no more than 5 and EOO is smaller than 20 km^2^. There is no reason to suppose that AOO, EOO, number of locations, number of subpopulations or the number or mature individuals are declining however due to its extremely small EOO we assessed as Vulnerable (VU) D2.

##### Remarks.

The shell morphology of the new species within the type locality varies considerably from almost flat shells to specimens with elevated spired and a more conical shell shape. Similar variability in the shell shape had been observed in the sympatric *Caucasopsis letsurtsume* sp. nov. It is curious whether both extreme variabilities could have the same environmental driver in the locality or if it could be a result of a parasitism. Many individuals are densely covered by calcareous inorganic precipitates, and some of them resemble a grain of sand without a recognisable shell shape. The operculum may also be densely covered by inorganic incrustations (Plate 20(9)).

#### Pontohoratia pichkhaiai

Taxon classificationAnimaliaLittorinimorphaHydrobiidae

Grego & Mumladze sp. nov.

26D3ABD3-65C0-57FE-97F6-49AD0323134E

http://zoobank.org/CC9EB289-9412-41F5-A761-EE76E6AD276D

Plates 20 (3); 21(2); 22(5–8); Fig. 16C, D

##### Type locality.

Georgia • Samegrelo, Mukhuri, Shisha Spring (შიშა წყარო, სოფელი მუხური); 42°37'47"N, 42°11'26"E; 255 m a.s.l.; sediment at bottom of spring zone.

**Plate 21.**
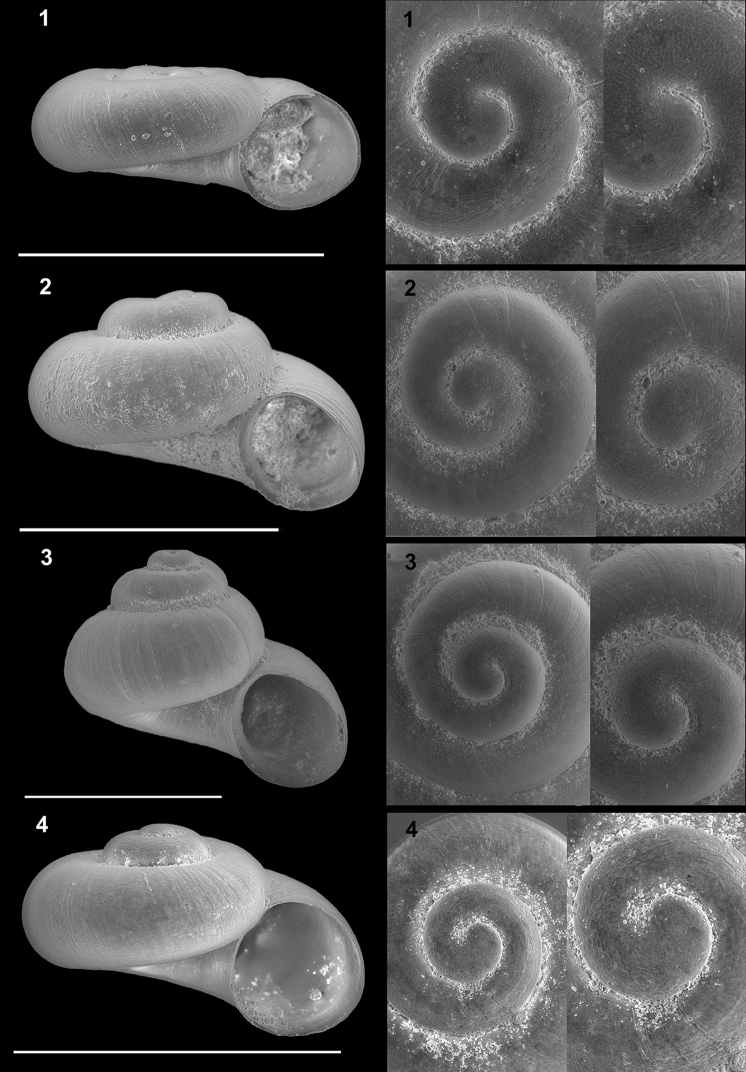
**1***Hausdorfenia pseudohauffenia* sp. nov., Racha, Shua Skhvava, Krikhula Spring, paratype SBMNH 633086 **2***Pontohoratia pichkhaiai* sp. nov., Samegrelo, Mukhuri, Shisha Spring, paratype SBMNH 633083 **3***P. vinarskii* sp. nov., Samegrelo, Chkhorotsku, Letsurtsume, Letsurtsume Cave, paratype SBMNH 633077 **4***P. mapeli* sp. nov., Samegrelo. Mukhuri, Kanti, Mapeli Spring, paratype SBMNH 635896. Scale bars: 1 mm. SEM SBMNH Vanessa Delnavaz.

##### Material.

***Holotype***: Georgia • 1 adult, dry; type locality; 10 May 2018; J. Grego, L. Mumladze and M. Olšavský leg.; ISU FM-T022-H. ***Paratypes***: Georgia • same as for holotype; ISU FM-T022-P1/9 dry, coll. JG F1044/9 dry; • same as for holotype; 11 October 2019; J. Grego, L. Mumladze and G. Bananashvili leg.; ISU FM-T022-P2/13 dry SBMNH 633083/1 dry, NHMW 113376/1 dry, HNHM 104685/1 dry, NHMB 563973/1 dry, SMF 358933/1 dry, ZMH 140637/1 dry, MNHN-IM-2016-7901, ZIN 1/504-2020/1 dry, coll. JG F1419/14 fry, coll. Glöer/1 dry.

**Plate 22.**
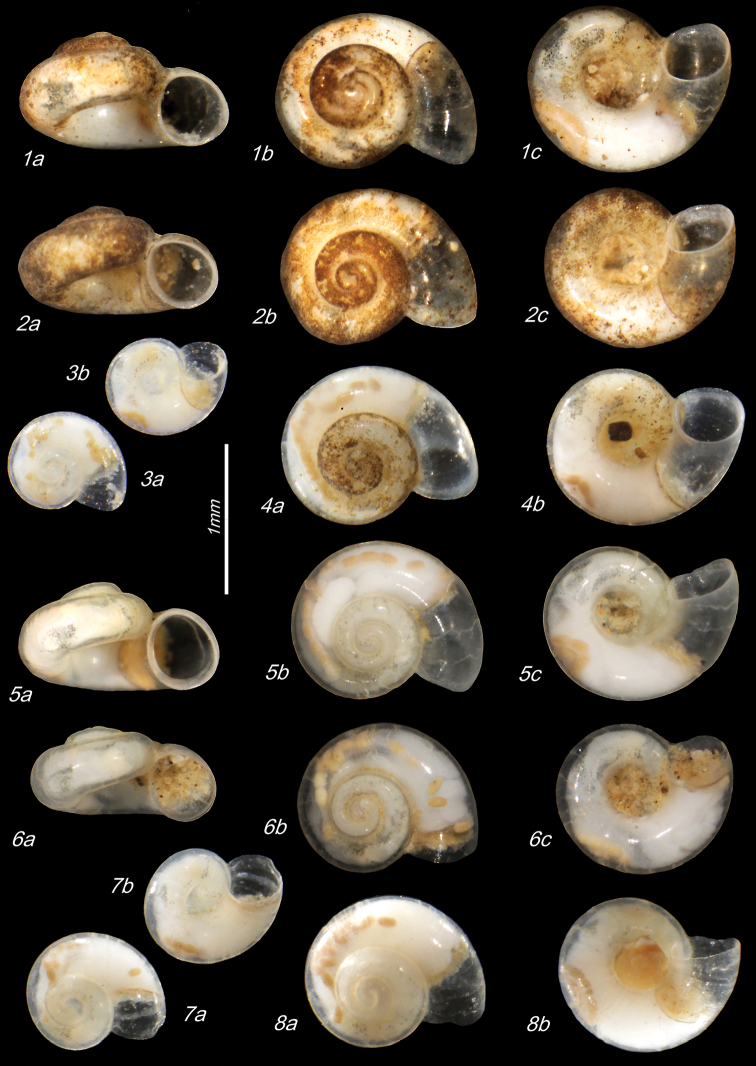
Specimens used for molecular and anatomical study **1–4***Pontohoratia mapeli* sp. nov., Samegrelo, Mukhuri, Mapeli Cave **5–8***P. pichkhaiai* sp. nov., Samegrelo, Mukhuri, Sisha Spring. The numbers correspond to individuals, and the letters represent the different views of the same individual. Photograph A. Rysiewska.

##### Diagnosis.

The new species differs from the geographically close *P. vinarskii* sp. nov. by its flatter shell and smaller, more rounded aperture. *P. mapeli* has a flatter shell with smaller, more rounded aperture.

##### Description.

***Shell***: planispiral small, discoid, the spire only a slightly pronounced and early whorls flat, umbilicus widely opened and protoconch surface pitted. Diameter 1.36–1.68 mm. The descending whorls separated by a deep suture. The shell wall is translucent, the surface whitish and smooth. The aperture proportionally small and circular with the labral peristome angled vs. the columellar axis. The aperture in a short distance joining the body whorl. Protoconch surface weakly pitted in its nuclear portion and abapically gradually changing into a smooth slightly malleated surface.

***Operculum***: reddish, circular, paucispiral, with central nucleus and smooth central callosity without forming a peg at its attachment.

***Holotype measurements***: H-0.87 mm; W-1.42 mm; BH-0.66 mm; BW-1.00 mm; AH-0.50 mm; AW-0.5 mm; CA: –45°.

***Anatomy***: the penis (Fig. 16C, D) simple, without any outgrowths, broad, slowly narrowing to its distal end.

##### Etymology.

Named after the avid speleologist Igor Pichkhaia (იგორ ფიჩხაია) from Chkhorotsku, who supported our research in the region of Samegrelo (Mingrelia).

##### Habitat.

Stygobiotic species. Empty shells and a few live individuals were found washed out from primary habitat at the bottom sediments of the spring lake of Shisha spring near Mukhuri. See the habitat of *Kartvelobia shishaensis* sp. nov.

##### Distribution.

Only known from the type locality.

##### Conservation status.

The number of known locations (1) is no more than 5 and EOO is smaller than 20 km^2^. There is no reason to suppose that AOO, EOO, number of locations, number of subpopulations or the number or mature individuals are declining however due to its extremely small EOO we assessed as Vulnerable (VU) D2.

#### Pontohoratia mapeli

Taxon classificationAnimaliaLittorinimorphaHydrobiidae

Grego & Mumladze sp. nov.

6F29FFEA-9D67-5B2C-AAA7-38CE7D741348

http://zoobank.org/B4E27364-8825-4C7F-8E4F-B60648230AF3

Fig. 16E, F ; Plates 20 (6); 21(4); 22(1–4)

##### Type locality.

Georgia • Samegrelo, Kanti Village near Mukhuri, Mapeli Spring (მაპელის წყარო, სოფელი კანტი); 42°38'23"N, 42°10'08"E; 290 m a.s.l.

##### Material.

***Holotype***: Georgia • 1 adult, dry; type locality; 06 May 2018; J. Grego, L. Mumladze and M. Olšavský leg.; ISU FM-T021-H. ***Paratypes***: Georgia • same as for holotype; col. JG F1060/5 dry; • same as preceding; 12 October 2019; J. Grego, L. Mumladze and G. Bananashvili leg.; ISU FM-T021-P1/ 40 dry and 69 wet, SBMNH 635896/6 dry, NHMW 113375/2 dry, HNHM 104684/2 dry, NHMUK 20191340/2 dry, NHMB 563972/2 dry, SMF 358931/2 dry, ZMH 140636/2 dry, MNHN-IM-2016-7900, ZIN 1/503-2020/2 dry, coll. JG F1413/40 dry and 69 wet, coll Glöer/1 dry.

##### Diagnosis.

The shell of the new species is more flat-discoid with a more open umbilicus, more rounded and proportionally smaller aperture vs. the geographically closest relatives: *P. vinarskii* sp. nov. and *P. pichkhaiai* sp. nov. The shell shape is somewhat similar to *H. pseudohauffenia*, but it can be differentiated by less pronounced protoconch, lower shell height to width ratio the proportionally smaller, more rounded aperture as well by its operculum lacking the knobby sculpture.

##### Description.

***Shell***: small, paucispiral, discoid with flat, only slightly pronounced spire and widely opened umbilicus. Diameter 1.37–1.51 mm. The inflated whorls are separated by a deeply cut sulcus. Protoconch surface covered by dense shallow pits. The shell surface whitish and translucent with smooth surface. The aperture round with labral peristome oblique to the columellar axis. The aperture barely attached at its upper columellar side to the body whorl. Protoconch surface covered by raised malleations gradually changing to a regular pitting towards the nucleus.

***Operculum***: reddish, circular, paucispiral with central nucleus, centrally thickened and elevated inward without peg.

***Holotype measurements***: H-0.57 mm; W-1.38 mm; BH-0.55 mm; BW-0.95 mm; AH-0.42 mm; AW-0.45 mm; CA: –40°.

***Anatomy***: the penis (Fig. 16E, F) proximally broad, with a broad, slightly marked outgrowth on its left side in the median part, and thin filament distally.

##### Etymology.

Name derived from the name of Mapeli (მაპელი) Spring in Kanti (კანტი) Village, the type locality of the species.

##### Habitat.

Stygobiotic species. See the habitat of *Caucasogeyeria chrysomallos* sp. nov.

##### Distribution.

Only known from the type locality.

##### Conservation status.

The number of known locations (1) is no more than 5 and EOO is smaller than 20 km^2^. There is no reason to suppose that AOO, EOO, number of locations, number of subpopulations or the number or mature individuals are declining however due to its extremely small EOO we assessed as Vulnerable (VU) D2.

#### Genus Hausdorfenia

Taxon classificationAnimaliaLittorinimorphaHydrobiidae

Grego & Mumladze gen. nov.

B16AB4C7-1738-5FC2-84D2-8FBC76A2DDC8

http://zoobank.org/45FEE38A-2C15-465F-A411-F21DDE5A522A

##### Type species.

*Hausdorfenia pseudohauffenia* Grego & Mumladze, sp. nov.

##### Species assigned to the genus.

*Hausdorfenia shareula* Grego & Mumladze, sp. nov.

##### Diagnosis.

The new genus differs from *Pontohoratia* Vinarski, Palatov & Glöer, 2014 by its flatter shell shape, more coarsely pitted protoconch and by its operculum with a distinct peg on its inner side. The molecular data support the closest relationship is to the genus *Kartvelobia* gen. nov.; however, its valviform shell shape is substantially different from the elongate oval shape and aperture morphology of its relative.

##### Etymology.

Name derived from Bernhard Hausdorf, Hamburg University (Germany), who contributed much to the study of Mollusca from whole Caucasus region.

##### Distribution.

Known from the karstic plateau of Shaori (შაორის კარსტული პლატო) and adjacent stygobiotic habitats (Fig. 15).

#### Hausdorfenia pseudohauffenia

Taxon classificationAnimaliaLittorinimorphaHydrobiidae

Grego & Mumladze sp. nov.

2871C1CA-50B8-5997-AACB-C79D9DB809F6

http://zoobank.org/B6B96400-015F-4FFA-8B11-79A1DD25AE38

Plates 20 (4, 7); 21(1)

##### Type locality.

Georgia, • Racha, Shua Skhvava, Zemo Krikhi, Krikhula Spring (მდინარე კრიხულა); 42°30'04"N, 43°12'27"E; 707 m a.s.l.

##### Material.

***Holotype***: Georgia • 1 adult, dry; type locality. 07 May 2018; J. Grego, L. Mumladze and M. Olšavský leg.; ISU FM-T011-H. ***Paratypes***: Georgia • same as for holotype; ISU FM-T011-P1/41 dry, SBMNH 633086/5 dry, NHMW 113370/1 dry, HNHM 104680/1 dry, NHMUK 20191336/1 dry, NHMB 563967/2 dry, SMF 358927/1 dry, ZMH140632/1 dry, MNHN-IM-2016-7897, ZIN 1/512-2020/1 dry, coll. JG F1025/41 dry, Glöer/1 dry; • same as preceding; 13 October 2019; J. Grego, L. Mumladze and G. Bananashvili leg.; ISU FM-T011-P2/19 dry, coll. JG F1426/19 dry. ***Other material***: Georgia • Racha, Kveda Tlughi, Kidobana Cave (კიდობანას მღვიმე); 42°26'1"N, 43°8'45"E; 1190 m a.s.l.; 07 May 2018; J. Grego, L. Mumladze and M. Olšavský leg. • Racha, Kveda Tlughi, Cholaburi karst spring; 42°26'8"N, 43°08'58"E; 1175 m a.s.l.; 07 May 2018; J. Grego, L. Mumladze and M. Olšavský leg. • Racha, Skhartali, Sakishore Cave (საკიშორეს მღვიმე); 42°26'32"N, 43°09'30"E; 1160 m a.s.l.; 07 May 2018; J. Grego, L. Mumladze and M. Olšavský leg. • Racha, Velevi, Dolabistavi Cave (დოლაბისთავის მღვიმე); 42°27'05"N, 43°10'39"E; 1170 m a.s.l.; 07 May 2018; J. Grego, L. Mumladze and M. Olšavský leg.

##### Diagnosis.

*Hausdorfenia pseudohauffenia* sp. nov. differs from most of the congeners by its flatter shell with elevated embryonal whorls and more backward protruding lower aperture vs. the columellar axis. Only *P. shareula* sp. nov. has a flatter shell, but its spire is sunken. The reddish operculum with an elevated peg-like structure differentiates the species from all relatives.

##### Description.

***Shell***: very flat paucispiral, 1.46–1.73 mm in diameter, discoid with flat or only very slightly elevated apex and widely expanded umbilicus. Descending 3¼ whorls separated by deeply depressed sulcus. Shell pale translucent, whitish surface, smooth with very faint axial growth lines. Aperture ovoid and in basal view declined left towards the body whorl, from which separated by a narrow gap. Lateral profile of the aperture is strongly sloped towards the apex. Protoconch with coarsely pitted surface converting adapically into a raised malleated surface.

**Figure 17.**
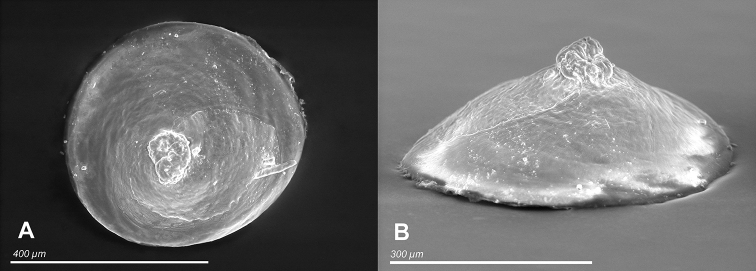
*Hausdorfenia pseudohauffenia* sp. nov. **A, B** operculum, SBMNH 633086. SEM SBMNH Vanessa Delnavaz.

***Operculum***: circular, with central nucleus, thickening at its central part. Inner side smooth centrally raising to a distinct internal peg at point of attachment to the retractor muscle (Fig. 17).

***Animal body***: without eye spots, milky white coloured with irregular small dispersed dark grey blotches visible through translucent shell.

***Holotype measurements***: H-0.82 mm; W-1.68 mm; BH-0.74 mm; BW-1.11 mm; AH-0.53 mm; AW-0.58 mm; CA: –48°.

***Anatomy***: not known.

##### Etymology.

Name derived from the shell morphology resemblance of the new taxon to the Middle European stygobiotic genus *Hauffenia* Pollonera, 1899.

##### Habitat.

Stygobiotic species. The studied material was found directly at the spring outlet among the larger debris. A few live individuals washed out from its stygobiotic habitat were attached to the undersides of boulders in the spring zone.

##### Distribution.

Aside from the type locality similar shells or fragments, likely belonging to the same species, were found in the following localities: Kidobana Cave, Cholaba Spring, Shakishore Cave and Dolabistavi Cave in the Shaori Basin.

##### Conservation status.

The number of known locations is no more than 5 and EOO is smaller than 20 km^2^. There is no reason to suppose that AOO, EOO, number of locations, number of subpopulations or the number or mature individuals are declining however due to its extremely small EOO we assessed as Vulnerable (VU) D2.

##### Remarks.

The sample from type locality yielded a few aberrant solute shells (scalarity) (Plate 20(7)).

#### Hausdorfenia shareula

Taxon classificationAnimaliaLittorinimorphaHydrobiidae

Grego & Mumladze sp. nov.

1C425869-E88C-5CBF-AF75-137953446DBA

http://zoobank.org/29BDD264-C578-49D8-945E-F4835070D996

Plate 20 (5)

##### Type locality.

Georgia • Racha, Nikortsminda, Tsivtskala 2 Spring on left bank of the Shareula River near the power station; 42°28'18"N, 43°03'54"E; 1084 m a.s.l.

##### Material.

***Holotype***: Georgia • 1 adult, dry; type locality; 06 May 2018; J. Grego, L. Mumladze and M. Olšavský leg.; ISU FM-T012-H. ***Other material***: Georgia • Fragmented shells; Racha, Nikorsminda, Shareula River Head (Shareula Cave); 42°28'12"N, 43°04'4"E; 1105 m a.s.l.; date; 20.08.2017, J. Grego leg.

##### Diagnosis.

The new taxon significantly differs from all congeners by its flat shape with spire hidden in apertural profile and its planorboid coiling, a unique feature within the southwestern Caucasus stygobiotic Gastropoda. Measurement comparison of *Pontohoratia* and *Hausdorfenia* species is given in Table 9.

**Table 9.** Measurement comparison of species in genera *Pontohoratia* Vinarski, Palatov & Glöer, 2014 and *Hausdorfenia* gen. nov.

*Genus species*	H	W	BH	BW	AH	AW	CA	H/W	AH / AW	W / BW	H/BH	H/AH	W / AW	H/(W- BW)
	mm	mm	mm	mm	mm	mm	deg.							
*Pontohoratia vinarskii* sp. nov. **Holotype**LT	1.08	1.47	0.87	1.00	0.63	0.55	-20	0.73	1.15	1.47	1.24	1.71	2.67	2.30
*Pontohoratia vinarskii* sp. nov. **Paratype**LT	1.11	1.42	0.95	1.00	0.66	0.53	-24	0.78	1.25	1.42	1.17	1.68	2.68	2.64
	1.46	1.50	1.13	1.08	0.75	0.63	30	0.97	1.19	1.39	1.29	1.95	2.38	3.48
*Pontohoratia mapeli* sp. nov. **Holotype**LT	0.57	1.38	0.55	0.95	0.42	0.45	-40	0.41	0.93	1.45	1.04	1.36	3.07	1.33
*Pontohoratia pichkhaiai* sp. nov. **Holotype**LT	0.87	1.42	0.66	1.00	0.50	0.50	-45	0.61	1.00	1.42	1.32	1.74	2.84	2.07
*Hausdorfenia pseudohauffenia* sp. nov. **Holotype**LT	0.82	1.68	0.74	1.11	0.53	0.58	-48	0.49	0.91	1.51	1.11	1.55	2.90	1.44
*Husdorfenia shareula* sp. nov. **Paratype**LT	0.53	1.34	0.50	0.82	0.48	0.42	-57	0.40	1.14	1.63	1.06	1.10	3.19	1.02

##### Description.

***Shell***: planispiral, discoid with planorboid (slightly hyperstrophic) coiling and 1.34 mm in diameter. Descending 2¼ whorls separated by a deep suture. Umbilicus very widely expanding. Shell colour milky white, surface smooth with very faint axial growth lines. Aperture circular, and its labral periphery is oblique to the columellar axis. It attached to the whole length of the adjacent body whorl by a narrow suture. Protoconch pitted over whole surface.

***Operculum***: not known.

***Animal body***: not known.

***Holotype measurements***: H-0.53 mm; W-1.34 mm; BH-0.50 mm; BW-0.82 mm; AH-0.48 mm; AW-0.42 mm; CA: –57°.

***Anatomy***: not known.

##### Etymology.

Name derived from the name of the Shareula River (მდინარე შარეულა), left tributary of the Rioni River, in which valley and a nearby tributary the new taxon was found.

##### Habitat.

The intact empty shell was found in sandy sediment at the spring head in a small cave. The supposed habitat is stygobiotic.

##### Distribution.

Except the type locality few similar fragments were found at the Shareula River Head (entrance of Shareula Cave).

##### Conservation status.

The number of known locations (2) is no more than 5 and EOO is smaller than 20 km^2^. There is no reason to suppose that AOO, EOO, number of locations, number of subpopulations or the number or mature individuals are declining however due to its extremely small EOO we assessed as Vulnerable (VU) D2.

##### Remarks.

*Hausdorfenia pseudohauffenia* sp. nov. and *P. shareula* sp. nov. display shell features different from other members of the genus, as well as a characteristic operculum with a peg (at least in the former taxon). Both represent a new genus different from *Pontohoratia*.

### Genus *Motsametia* Vinarski, Palatov & Glöer, 2014

#### Motsametia borutzkii

Taxon classificationAnimaliaLittorinimorphaHydrobiidae

(Shadin, 1932)

2AA6C99D-3622-515B-AEC9-77C931047E85

Fig. 18

Motsametia borutzkii M. V. Vinarski, D. M. Palatov & P. Glöer, 2014 – J. Nat. Hist., 48: 2241 fig. 2B, 2244 fig. 5A, and 2249 fig. 7E
Horatia borutzkii A. V. Shadin, 1932 – Arch. Molluskenkd. 64: tab. 1, fig. 1.

##### Conservation status.

The species is known from a single location and AOO is smaller than 10 km^2^. There is also indication of stochastic human driven habitat pollution and introduction of possibly competing invasive species (*Ferrissia californica*) (Vinarski and Palatov 2018) leading to severe population decline since 2009 with scarce occurrence of live individuals. Therefore, it is assessed as Critically endangered (EN) B2.

##### Remarks.

Since the field work of Dimitry Palatov in 2009–2012 (Vinarski et al. 2014), we have recorded a continuous population decrease of *M. borutzkii* at the only known locality, with live individuals becoming scarce. It is possible that pollution of groundwater from settlements just above the cave could influence the groundwater quality. The pollution of the cave stream can be traced by increased micro-plastic particles present in the cave sediments. The population of Ferrissia cf. californica (Rowell, 1863) in the cave does not seem to have an invasive character, but could lead to a food competition with *M. borutzkii* (Vinarski and Palatov 2018). The presence of *Ferrissia* seem to be an incidental migrant via sinking surface water.

**Figure 18.**
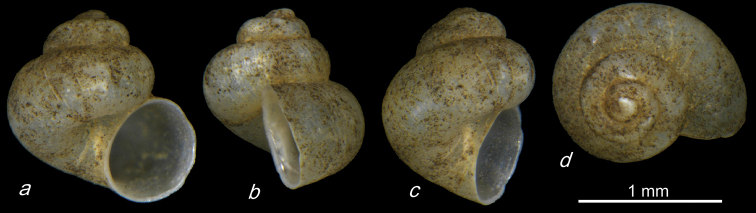
*Motsametia borutzkii* (Shadin, 1932) **A–D** Imereti, Kutaisi, Iazoni (Tskal-Tsiteli) Cave. Photograph J. Grego.

**Figure 19.**
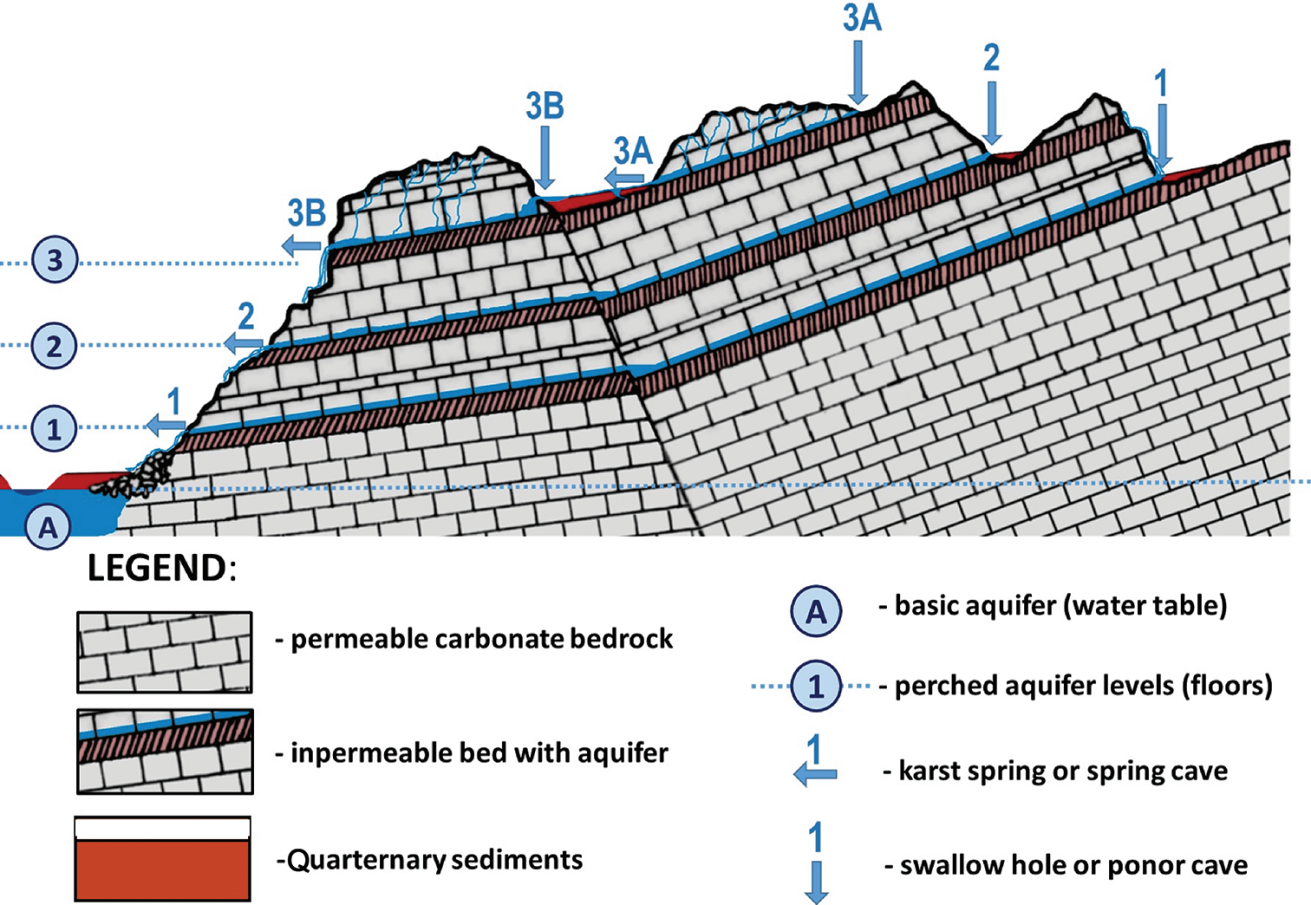
Multiple Level Episaturation: Formation of multiple levels (floors) of perched aquifers on permeable carbonates with subhorizontal beds and with periodical inclusion of impermeable beds. The levels of episaturation can have no or just minimal water communication, which can lead in a development of isolated stygobiont ecosystems in each level. Similar geology can be found in carbonates framing the southwestern Caucasus. Springs are frequently emerging high on hillslopes or near the middle of the cliff through caves and waterfalls.

### Discussion

The molecular data confirmed the presence of the representatives of the subfamily Sadlerianinae Szarowska, 2006 in hypogean habitats of the southwestern Caucasus. The extraordinary high diversity suggests a longer isolation of populations presumably in isolated cave systems and their allopatric development. Some of the species (e.g. within the genera *Caucasopsis* gen. nov. or *Imeretiopsis* gen. nov.) in relatively close but isolated cave streams show molecular differences, while others (e.g., *Kartvelobia sinuata* sp. nov. or *Caucasogeyeria gloeri* sp. nov.) have a distribution pattern over a larger aquifer under a single isolated limestone plateau Pakhe. The isolated aquifers of the Samegelo, Imereti and Racha regions have typical features of episaturation or *Perched water table* (Vepraskas and Lindbo 2012), which means isolated water tables (aquifers) elevated above the aquifers of the lowlands. Bedded Mesozoic limestones in the studied Georgian regions are characterised by mostly thick subhorizontal beds occasionally separated by less permeable or impermeable sandstones or dolomite beds. Such a hydrological separation of beds over a large area results in a large number of karst springs situated high on the hillslopes or near the middle of cliffs, emerging frequently as waterfalls directly from a cave spring or spring zone (e.g., waterfalls at Toba, Oniore, Kinchkha and caves in Motena, Mapeli, Dolabistavi). The perched water tables at a highly elevated places allowed a development of another perched water table (or sometimes tables) situated under the limestone beds below the impermeable rocks of the higher water table comprises a system of *Multiple Levels Episaturation*. This can result in several floors (or altitude levels) of perched water tables, where the aquifers are situated one above the other, separated by impermeable beds. Such a vertical isolation (Fig. 19) could also lead to isolated development of their stygobiont fauna, and could explain the high diversity over the relatively small area. As an example, we can use the distribution pattern of *K. sinuata* sp. nov. found in the springs of the highest level of perched aquifer of the Pakhe Plateau emerging in its slopes or at the middle of its cliffs. In contrast, the springs emerging from the lower-positioned perched aquifer of the same plateau around its base host different species or maybe subspecies of the same genus (e.g., morphologically different minute inflated population of K. cf. sinuata and *K. kinchkha* sp. nov. at N foots of the plateau and a K. cf. kinchkha from southern foot of the same plateau). More molecular data from both perched aquifers could confirm their phylogenetic separation. Most of the sampled localities host approximately three or four sympatric stygobiotic species of different genera, usually *Caucasogeyeria*, *Kartvelobia* and *Pontohoratia* or *Hausdorfenia*, with one species of the following three genera by region: *Caucasopsis* in Samegrelo, *Imeretiopsis* in Imereti and a new belgrandinellinid genus in Racha. Additionally, many of the springs host a *Tschernomorica* species which occasionally also inhabited the stygobiont habitats including caves (e.g., Nazodelavo, or Sataplia Caves). In majority of localities we found only one representative of each genus, only very seldom two species of the genus *Caucasogeyeria* could be found as sympatric (e.g., *C. colchis* and *C. gloeri* in Nakhriduri Spring, *C. pseudocolchis* and C. cf. gloeri. in Shurubumu or *C. chrysomallos* and C. cf. gloeri in Mapeli Cave.

The finding of two new species with many individuals living on slime covering tree roots inside a cave pond confirmed the phreatic rhizospere (Jasinska et al. 1996) as a preferred habitat also for gastropods (Grego et al. 2017). The tree roots secret a variety of reach nutrients by a process called *Root Exudation* (Canarini et al. 2019) and support the growth of symbiotic bacteria and fungi. The secretion can be massive, representing 20–40% of the carbon fixed by photosynthesis (Badri and Vivanco 2009). It seems that some of the hypogean gastropods found in the phreatic rhizosphere feed on microbial mats associated with the plant roots, or maybe directly on the root material, as we had seen in a small cave spring at the middle Shareula River valley.

Knowing the hydrogeological preconditions and rich geological history of the area, we believe a much larger stygobiont diversity exists than presented in this study. During our studies we sampled only a very small portion of suitable habitats (springs and caves) over the studied area, and large karstic expanses of the southwestern Caucasus remain unexplored.

The new genera we established in consideration of the shell morphology supported by the anatomical and molecular data we got from the type species. However, due to lack of live collected material, some species had to be described solely based on shell morphology characters and placed into provisional genera until the molecular data can be obtained. Many of the species are scarce and live specimens were never found. We believe that it is important to bring attention to such species and, due to the absence of live material, treat their description as in the case fossil taxa and use only available shell characters for species characterisation. Especially considering the rapidly changing environment and increasing pollution, the recognition of stygobiont species has ecological importance. While waiting decades for molecular data to be generated, some species could become extinct. It seems more expedient to treat them as provisional genera now and to correct their generic position in the case a live specimen is ever found. Additionally, the species established by their shell morphology can inspire and provide a taxonomic framework for future researchers to perform more extensive field work needed to recover complementary living material and new taxa in the future.

### Conclusions

With the present study we confirm the extraordinarily high stygobiotic gastropod diversity of the southwestern Caucasus. The high diversity on the generic level was supported by molecular and anatomical data. The taxonomic position of the genera “*Geyeria*” and “*Paladilhiopsis*” sensu Starobogatov, 1962 and *Pontohoratia* Vinarski, Palatov & Glöer, 2014 were solved, as well the assignment of five new genera in the subfamily Sadlerianinae Szarowska, 2006. The stygobiotic gastropod species radiation of Caucasus was more than doubled from previously known 16 species-level taxa (placed in five genera) to up to 40 taxa within eight genera. This further corroborates the “biodiversity hotspot” status of the western Great Caucasus karst region. It is very likely that future intensive field research could reveal even higher hypogean biodiversity not only in the class Gastropoda, but also for other subterranean freshwater invertebrates. The results of the study of Belgrandiellinae Radoman, 1983 from the region will be subject of the next report, which is in preparation.

## Supplementary Material

XML Treatment for Kartvelobia

XML Treatment for Kartvelobia sinuata

XML Treatment for K. cf. sinuata

XML Treatment for Kartvelobia kinchkha

XML Treatment for Kartvelobia shishaensis

XML Treatment for Imeretiopsis

XML Treatment for Imeretiopsis prometheus

XML Treatment for Imeretiopsis gorgoleti

XML Treatment for Imeretiopsis nakeralaensis

XML Treatment for Imeretiopsis cameroni

XML Treatment for Imeretiopsis iazoni

XML Treatment for Caucasopsis

XML Treatment for Caucasopsis letsurtsume

XML Treatment for Caucasopsis olsavskyi

XML Treatment for Caucasopsis egrisi

XML Treatment for Caucasopsis cf. egrisi

XML Treatment for Caucasogeyeria

XML Treatment for Caucasogeyeria gloeri

XML Treatment for Caucasogeyeria cf. gloeri

XML Treatment for Caucasogeyeria ignidona

XML Treatment for Caucasogeyeria colchis

XML Treatment for Caucasogeyeria pseudocolchis

XML Treatment for Caucasogeyeria chrysomallos

XML Treatment for Pontohoratia

XML Treatment for Pontohoratia vinarskii

XML Treatment for Pontohoratia pichkhaiai

XML Treatment for Pontohoratia mapeli

XML Treatment for Hausdorfenia

XML Treatment for Hausdorfenia pseudohauffenia

XML Treatment for Hausdorfenia shareula

XML Treatment for Motsametia borutzkii
